# CD37 is a safe chimeric antigen receptor target to treat acute myeloid leukemia

**DOI:** 10.1016/j.xcrm.2024.101572

**Published:** 2024-05-15

**Authors:** Benjamin Caulier, Sandy Joaquina, Pascal Gelebart, Tara Helén Dowling, Fatemeh Kaveh, Moritz Thomas, Luka Tandaric, Patrik Wernhoff, Niveditha Umesh Katyayini, Cara Wogsland, May Eriksen Gjerstad, Yngvar Fløisand, Gunnar Kvalheim, Carsten Marr, Sebastian Kobold, Jorrit M. Enserink, Bjørn Tore Gjertsen, Emmet McCormack, Else Marit Inderberg, Sébastien Wälchli

**Affiliations:** 1 Translational Research Unit, Section for Cellular Therapy, Department of Oncology, Oslo University Hospital, Oslo, Norway; 2 Institute for Cancer Research, Department of Molecular Cell Biology, Oslo University Hospital, Oslo, Norway; 3 Center for Cancer Cell Reprogramming (CanCell), Institute for Clinical Medicine, Faculty of Medicine, University of Oslo, Oslo, Norway; 4 Department of Clinical Science, Precision Oncology Research Group, University of Bergen, 5021 Bergen, Norway; 5 Centre for Pharmacy, Department of Clinical Science, University of Bergen, Bergen, Norway; 6 Centre for Cancer Biomarkers (CCBIO), University of Bergen, Bergen, Norway; 7 Institue of AI for Health, Helmholtz Munich, 85764 Neuherberg, Germany; 8 School of Life Sciences Weihenstephan, Technical University of Munich, Freising, Germany; 9 Department of Obstetrics and Gynecology, Haukeland University Hospital, Bergen, Norway; 10 Division of Clinical Pharmacology, Department of Medicine IV, University Hospital, Ludwig-Maximilians-Universität München, Munich, Germany; 11 German Center for Translational Cancer Research (DKTK), Partner Site Munich, Munich, Germany; 12 Einheit für Klinische Pharmakologie (EKLiP), Helmholtz Zentrum München, Research Center for Environmental Health (HMGU), Neuherberg, Germany; 13 Section for Biochemistry and Molecular Biology, Faculty of Mathematics and Natural Sciences, University of Oslo, Oslo, Norway; 14 Department of Medicine, Hematology Section, Haukeland University Hospital, Bergen, Norway

**Keywords:** chimeric antigen receptor, CAR T cell, AML, acute myeloid leukemia, immunotherapy, CD37, hematopoietic stem cell, patient-derived xenograft

## Abstract

Acute myeloid leukemia (AML) is characterized by the accumulation of immature myeloid cells in the bone marrow and the peripheral blood. Nearly half of the AML patients relapse after standard induction therapy, and new forms of therapy are urgently needed. Chimeric antigen receptor (CAR) T therapy has so far not been successful in AML due to lack of efficacy and safety. Indeed, the most attractive antigen targets are stem cell markers such as CD33 or CD123. We demonstrate that CD37, a mature B cell marker, is expressed in AML samples, and its presence correlates with the European LeukemiaNet (ELN) 2017 risk stratification. We repurpose the anti-lymphoma CD37CAR for the treatment of AML and show that CD37CAR T cells specifically kill AML cells, secrete proinflammatory cytokines, and control cancer progression *in vivo*. Importantly, CD37CAR T cells display no toxicity toward hematopoietic stem cells. Thus, CD37 is a promising and safe CAR T cell AML target.

## Highlights

• CD37 is expressed on AML blasts
• CD37 expression correlates with ELN 2017 risk stratification
• CD37CAR T cells are efficient against AML *in vitro* and *in vivo*
• CD37CAR T cells do not deplete myeloid progenitors

## Graphical abstract

**Figure:**
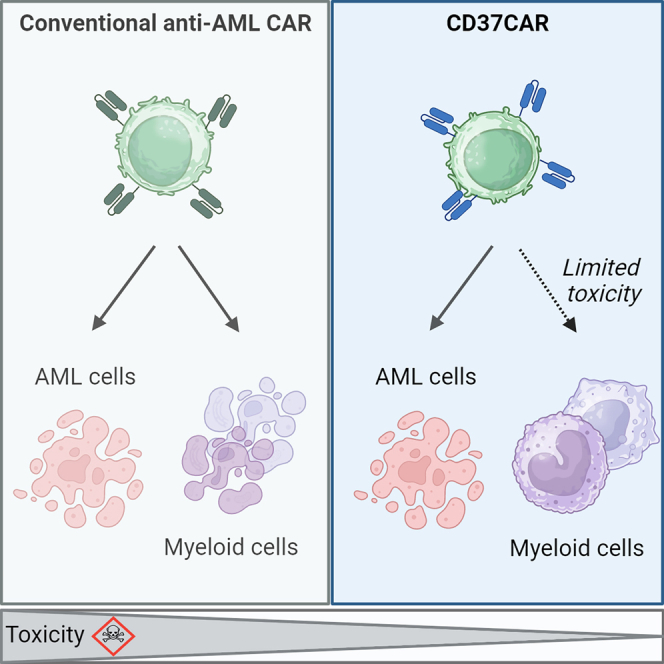

## Teaser

Caulier et al. demonstrate that CD37, a B cell marker, is expressed on the cell surface of the majority of primary AML samples. A CAR construct directed against CD37 controls AML progression, suggesting that CD37CAR T cells represent a promising solution for AML treatment.

## Introduction

Acute myeloid leukemia (AML) is an aggressive blood cancer that remains difficult to treat.^1^ The first line of treatment against AML relies on repeated high-dose chemotherapy frequently consolidated with allogeneic hematopoietic stem cell transplantation (HSCT). Improved knowledge of leukemia genetics has led to a recent update of the European LeukemiaNet (ELN) risk classifications, response criteria, and therapy guidelines.^2^^,^^3^ First-line and relapse therapy are constantly developed for AML and include antibody-drug conjugate (ADC) gemtuzumab ozogamicin targeting CD33; monoclonal antibodies (mAbs) targeting CD44; and CD123, bi-specific T cell engagers (BiTEs) or immune checkpoint inhibitors.^4^^,^^5^^,^^6^^,^^7^ Unfortunately, 50% of AML patients relapse after initial remission.^8^ This situation is presumably due to the presence of a persisting population of leukemic stem cells (LSCs), which are known to initiate and maintain the disease by exhibiting properties of self-renewal, cell cycle quiescence, and chemo-resistance.^9^ The outcome of patients with relapsed/refractory (R/R) AML is particularly dismal, with no more than 10% of overall survival (OS) at 3 years.^2^^,^^10^ Recently, adoptive cellular therapies (ACTs) with T cells expressing chimeric antigen receptors (CARs) have been investigated in R/R AML. Some of these CARs showed promising pre-clinical results by targeting the myeloid cell-restricted CD33^11^^,^^12^ and stem cell marker interleukin (IL)-3 receptor (CD123)^13^ as well as other leukemia-associated antigens. CD33 and CD123 are currently the main targets under evaluation in clinical trials.^7^^,^^14^ Combinatorial approaches to prevent disease relapses associated with antigen loss, as observed in B cell malignancies,^15^^,^^16^ are also being tested. However, the main challenge of AML CAR T therapy is that myeloblasts frequently share target expression with healthy hematopoietic stem cells (HSCs), or targets are also broadly expressed outside the hematopoietic system,^7^ contributing to the off-tumor toxicity of the CAR T cell therapy. There is a strong need to develop safer CAR designs against R/R AML to achieve objective long-term remission.

CD37 is a cell-surface glycoprotein that belongs to the four-transmembrane tetraspanin superfamily,^17^ known to interact with a range of adhesion molecules, growth and signaling receptors, as well as other tetraspanins, all of which are involved in membrane organization, signal transduction, survival, and apoptosis.^17^^,^^18^^,^^19^ Unlike other broadly distributed tetraspanins (e.g., CD9, CD81, CD151), CD37 expression appears restricted to B cells, although it has been reported at far lower levels in other healthy hematopoietic cells.^20^^,^^21^^,^^22^^,^^23^^,^^24^^,^^25^ In contrast, CD37 expression in cancer has been demonstrated in B cell non-Hodgkin lymphoma (B-NHL),^26^ but it was recently observed that CD37 expression is not a hallmark of all B cell malignancies; some recent studies demonstrated that only 60% of follicular lymphoma (FL)^27^ and only 40% of diffuse large B cell lymphoma (DLBCL)^28^ tested positive. In addition, CD37 was also detected in T cell lymphoma,^24^^,^^29^ whereas one report detected it in AML at the protein level^24^ and another showed increased mRNA level in AML patient samples.^30^ Finally, CD37 is druggable and several antibody-based molecules have been exploited,^20^^,^^24^^,^^31^^,^^32^^,^^33^^,^^34^^,^^35^ mainly to treat B-NHL.

In the present work, we undertook a deeper study of CD37 expression in AML samples covering the full spectrum of the disease and detected CD37 in the majority of AML patients. We observed that, in contrast to CD33, CD37 protein expression showed an excellent correlation with the ELN 2017 patient prognostic stratification. The HH1 anti-CD37 antibody-based CAR (CD37CAR), previously tested in B-NHL,^32^ was tested against AML and shown to be of comparable efficiency to the CD33CAR, with the advantage of being less toxic against healthy cells. The present data pre-clinically validate CD37 as a safe and efficient target for CAR T cell therapy in AML.

## Results

### CD37 is expressed in AML patient samples

**Figure 1:**
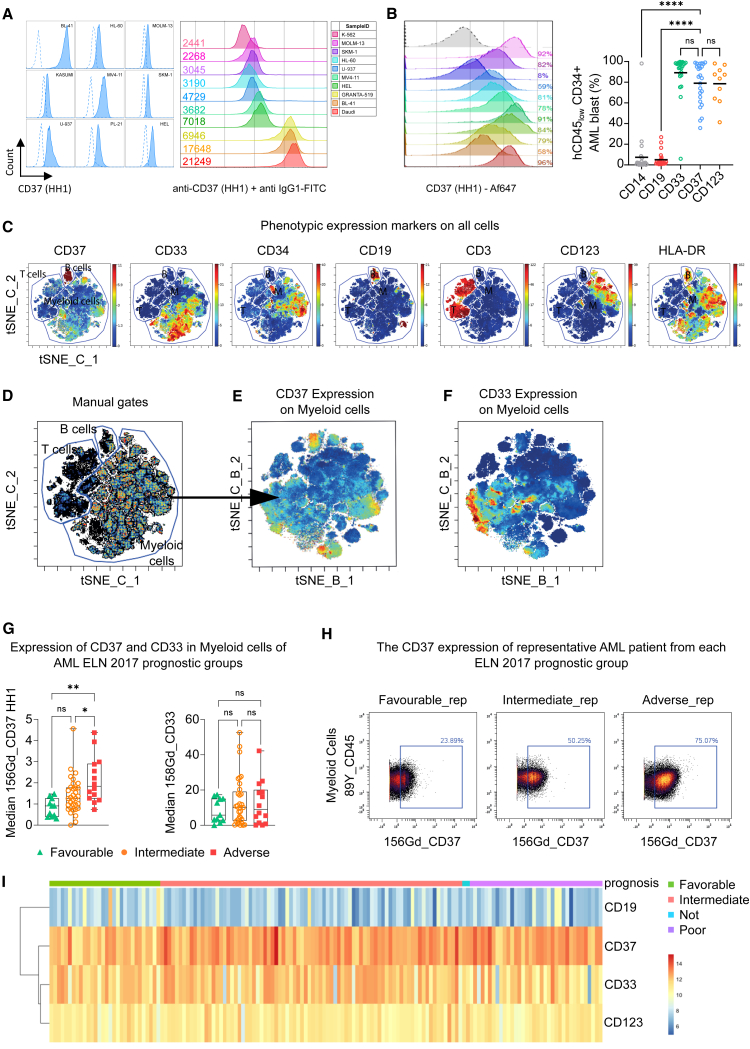
CD37 expression on AML (A) CD37 staining of different AML cell lines and BL-41 (B cell lymphoma) HH1 antibody. The dotted line represents the corresponding murine IgG1 isotype control (left). Surface CD37 protein quantification of K-562 (CML), U-937, MV4-11, MOLM-13, HEL (AML), and BL-41 (B cell lymphoma) (right). The murine IgG1 anti-CD37 mAb clone HH1 was used for the detection. A murine IgG1 isotype was used to set the background. The numbers indicate the amount of CD37 molecules per cell. (B) CD37 staining in primary samples. (Left) Percentage of hCD45^+^ CD37^+^ cells in primary AML samples used as PDX models (*n* = 11). The anti-CD37 HH1 was used, and the dotted line represents the isotype control. (Right) Percentage of CD14, CD19, CD33, CD37, and CD123 surface proteins on hCD45_low_ CD34^+^ AML blast population from primary BM samples (*n* = 25). Black bar represents the mean. One-way ANOVA followed by Dunnett’s multiple comparison tests is displayed, ∗∗∗∗*p* < 0.0001, ns = not significant. (C) Expression of CD37, CD33, CD34, CD19, CD3, CD123, and HLA-DR are shown as heat dot plot on tSNE-Cuda (tSNE_C) of concatenated AML patients (*n* = 59). The markers shown are indicative of cell subset , B cells (CD19), T cells (CD3), and AML (CD33, CD34, CD123, and HLA-DR). Manual gates are annotated. (D) The tSNE-Cuda here with manual gates drawn guided by CD3 and CD19. Further analysis was made with the myeloid cell gate. (E) The expression of CD37 in myeloid cells here shown as heat on the tSNE-Cuda of the myeloid cell gate from (B). (F) The expression of CD33 in myeloid cells here shown as heat on the tSNE-Cuda of the myeloid cell gate from (D). (G) The raw median expression intensity of CD37 and CD33 in myeloid cells grouped according to ELN 2017 risk stratification (*n* = 59). One-way ANOVA multiple comparison test identified that the expression of CD37 was found to be significantly increased for the adverse patient group in comparison to the good (*p* = 0.0011) and intermediate (*p* = 0.0247) patient group. No significant correlation of CD33. One-way ANOVA found no significance when investigating the expression of CD33 between ELN 2017 risk groups. (H) Bi-axial plots of CD45 vs. CD37 expression on myeloid cells of a patient representative from each ELN 2017 risk group showing a 25% stepwise increase between the risk groups. The tSNE-Cuda of the concatenated AML patients (*n* = 59) using only myeloid cells colored by population annotated as MC. (I) *CD19*, *CD37*, *CD33*, and *CD123* (*IL3RA*) gene expression analysis from RNA-seq dataset (TCGA-LAML, *n* = 150). The heatmap shows the normalized RNA-seq counts (DESeq2) for the four genes and each column represents a patient. Patient clustering was performed according to the prognosis (favorable, intermediate, poor).

We tested the presence of CD37 at the cell surface using two different anti-CD37 antibodies (Figure S1A). When the anti-CD37 antibody M-B371 was used on the pro-monocytic myeloid leukemia cell line, U-937, a negative-to-weak signal was observed. However, when the cells were stained with the anti-CD37 HH1 antibody,^36^ a clear binding was detected. The difference between the antibodies was much less marked when staining B cell lymphoma. In order to test the possibility that HH1 recognized another protein at the surface of AML cells, we generated a U-937 knockout for CD37, U-937^*CD37KO*^, and observed no binding of HH1 (Figures S1A and S1B). These data suggest that the anti-CD37 HH1 antibody was much more sensitive to the CD37 protein expressed on AML cells. We then tested whether a difference in the three main isoforms of CD37 could affect the recognition. First, we studied their expression in AML, and although the distribution of mRNA isoforms was different between normal and AML bone marrow (BM), all isoforms were expressed (Figure S1C). Nevertheless, when we overexpressed these isoforms in HEK cells, both HH1 and M-B371 antibodies’ recognition was restricted to the isoform 1 (Figures S1D–S1E). Second, we tested whether the glycosylation status of CD37 might interfere with antibody recognition, but treatment of target cells with neuraminidase altered neither its recognition nor CAR activity (Figures S1F and S1G). We confirmed the ability of HH1 to detect CD37 on different AML cell lines (Figure 1A left) and quantified CD37 antigen density (Figure 1A right). CD37^high^ B cell lymphoma BL-41 and CD37^null^ chronic myelogenous leukemia K-562 cell lines were used as references (Figure 1A right). AML cell lines displayed generally lower levels of CD37 than B cell lymphomas and required staining with a specific antibody, elucidating the previous conflicting reports on CD37 positivity. We also investigated CD37 expression in several patient-derived xenograft (PDX) models of AML and observed broad expression with an antigen density equivalent to AML cell lines (Figure 1B left and S1H). In a series of patient biopsies (*n* = 25; Figure S2A), we compared the level of CD37 positivity to the validated CAR targets CD33 and CD123 in AML^7^ (Figure 1B right; Figures S2B and S2C) and detected similar positivity on the bulk population. Furthermore, the presence of CD37 was detected in AML LSC populations (Figures S2D and S2E). This prompted us to further evaluate CD37 cell-surface expression in a larger and more extensive AML patient cohort, and we examined the CD37 expression using the HH1 antibody in diagnostic peripheral blood samples from 59 AML patients and five healthy donors. These patients were stratified according to ELN 2017 risk classification at diagnosis and complete remission after standard induction therapy was used as an indicator of therapy response/resistance (Table S1). An antibody panel of 40 markers was designed to identify phenotypic cell subsets of the samples including leukemic blasts and healthy populations. The 11 intracellular markers allow for investigation of signaling pathways regulating the differentiation, proliferation, and survival of myeloid cell subsets.^37^ From the panel, we used 23 extracellular markers for unsupervised clustering (FlowSOM) and dimensionality reduction (GPU-accelerated implementation of t-distributed stochastic neighbor embedding, tSNE-Cuda) (Figure 1C). As expected, high expression of CD37 was observed in the CD19-expressing B cell population, whereas CD3-expressing T cell populations had no detectable expression of CD37. In contrast, CD37 expression was observed on myeloid cells, and we went on to further investigate the distribution of CD37 in myeloid subsets by FlowSOM and tSNE-Cuda (Figures 1D–1F). The 10 myeloid meta-clusters (MCs) were annotated by application of marker expression and marker enrichment modeling (Figure S3A). They were further manually annotated based on their expression profile and healthy abundance, and relative MC abundance was visualized in a stacked bar plot (Figure S3B) in which healthy donors and patients were ordered based on similar MC distribution patterns (Figure S3C). The MC b_02 was the major MC prevalent in most of the patients and includes the most cells. We investigated the patients’ individual CD37 expression levels and discovered significant association of median CD37 expression with the ELN 2017 risk classification (Figures S4A and S4B). Tukey’s multiple comparisons test found a significant difference between good and adverse risk groups (*p* = 0.0011), and between intermediate and adverse risk groups (*p* = 0.0247) (Figures 1G and 1H). Furthermore, CD33 protein expression showed no correlation to ELN 2017 risk groups (Figure 1G). Previous data-mining studies have shown a correlation between *CD37* mRNA expression and the French American British (FAB) phenotypic classification system, with the FAB subgroup M5 showing higher expression than the other subgroups.^30^ However, in our AML dataset, we found no significant correlation between FAB classification and CD37 median expression (Figure S3D), which could be due to the comparatively small number of samples or a discrepancy between mRNA presence and protein expression.

We used the TCGA-LAML RNA sequencing (RNA-seq) dataset and grouped patients according to the clinical prognostic factors to identify differentially expressed genes (*n* = 150; Figure 1I). To assess the overall similarity between samples, we performed unsupervised clustering and evaluated the expression of *CD19*, *CD33*, *CD37*, and *CD123*. Extracting significantly differentially expressed genes revealed that *CD37* was overexpressed compared to *CD33* and *CD123* (*p* = 0.00188), while, as expected, *CD19* expression levels were much lower (*p* = 0.0066). We further investigated microarray data from the Microarray Innovations in Leukemia (MILE) study^38^ (*n* = 254; Figure S5A) and a pool of AML samples^39^^,^^40^^,^^41^ (high risk, *n* = 242, Figure S5B; and favorable/intermediate risk *n* = 1832, Figures S5C–S5E) and compared CD37 expression to that of hematopoietic stem and progenitor cells (HSPCs) and differentiated myeloid cells from adult healthy BM.^42^ Next, we evaluated *CD37* expression using a single-cell RNA-seq dataset consisting of 28,404 healthy and malignant cells from 15 patients diagnosed with AML^43^ (Figures S6A and S6B). On a transcriptomic level, *CD37* expression was predominantly restricted to populations of malignant cells (Figure S6C), showing consistent expression among all AML patients exhibiting malignant cellular populations (Figure S6D).

Together, these data confirm that *CD37* mRNA is translated and expressed at the surface of AML cells, but its detection efficiency might only be restricted to some antibodies, thus making CD37 a potential HH1 antibody-based CAR target.

### CD37-targeting CAR T cells demonstrate potent activity against AML cell lines

**Figure 2:**
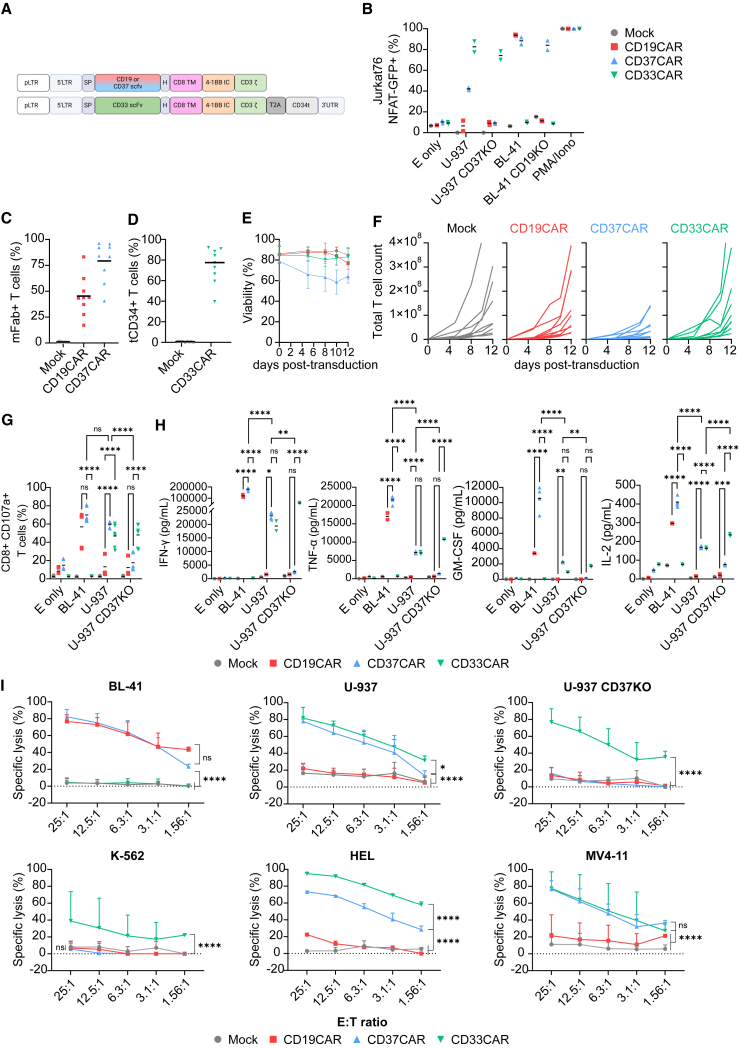
CD37CAR T cells against AML (A) Design of retrovirus vectors encoding second-generation CARs comprising a murine anti-human single-chain variable fragment (scFv), the CD8a hinge and transmembrane domains, the cytoplasmic domain of 4-1BB costimulatory molecule, and the CD3ζ subunit of the TCR. Sequences are provided in Figure S6. (B) Percentage of activation of J76^NFAT-GFP^ cells transduced with either mock, CD19^−^, CD37^−^, or CD33CAR and co-cultured for 24 h with the indicated cell lines or left alone (E only). E:T = 1:2 (*n* = 2 independent experiments, mean). (C–F) Percentage of CAR expression (C) and (D), viability (E), and expansion in total T cell count (F) of T cell donors bearing the CAR constructs (*n* = 9) for 12 days post transduction. The CAR expression was detected using an anti-murine fragment antigen-binding (Fab) antibody for CD19^−^ and CD37CAR and an anti-CD34 mAb for CD33CAR, at day 4 post-transduction. The black bar represents the mean. (G) Percentage of CD8^+^ CD107a^+^ T cells upon 6 h of co-culture with the indicated cell lines or left alone (E only). E:T = 1:2 (*n* = 3 donors in duplicates, mean). One-way ANOVA followed by Dunnett’s multiple comparison tests is displayed, ∗∗∗∗*p* < 0.0001, ns = not significant. (H) Secretion (pg/mL) of IFN-γ, TNF-α, IL-2, and GM-CSF in the supernatant of T cell co-culture with the indicated cell lines or left alone (E only) after 24 h. E:T = 1:2 (*n* = 2 donors except CD37CAR *n* = 4, mean), ∗∗*p* < 0.01, ∗∗∗*p* < 0.001, ∗∗∗∗*p* < 0.0001, ns = not significant. (I) Specific cytotoxicity of T cells incubated for 4 h with BL-41; 6 h with U-937, U-937 CD37KO, K-562, and HEL; or 7 h with MV4-11. Different E:T ratios (*n* = 4 donors except HEL = 2, mean ± SD). Two-way ANOVA followed by Tukey’s (G) and (H) or Dunnett’s (I) multiple comparisons tests. Comparisons versus CD37CAR are displayed, ∗∗∗∗*p* < 0.0001, ns = not significant.

Having established that CD37 is broadly expressed in AML blast cells, we evaluate the potency of CD37CAR T cells against AML, comparing it to anti-CD19 fmc63-based CAR (CD19CAR) as a negative control and to anti-CD33 gemtuzumab ozogamicin-based CAR,^11^ CD33CAR, as a validated anti-AML CAR (Figure 2A). Given that the CD33CAR is humanized, we added a truncated CD34 tag to enable its detection (construct sequences provided in Figure S7). We confirmed CD37CAR specificity toward CD37 using a reporter system in which single clones of Jurkat76^44^ expressing GFP under the control of NFAT (J76^NFAT-GFP^)^45^ were transduced with CD19^−^, CD37^−^, or CD33CAR. The CAR-J76^NFAT-GFP^ cells were co-cultured with BL-41 (CD19^+^, CD37^high^, CD33^−^) and U-937 (CD19^−^, CD37^low^, CD33^+^) as well as cell lines in which either CD19 or CD37 was knocked out (Figures S8A–S8C). We observed a strong GFP-positive specific signal from CAR-J76^NFAT-GFP^ cells in the presence of their cognate target (Figures 2B and S8D). Importantly, even the CD37^low^ U-937cell line could induce a specific CD37CAR response, suggesting a strong functional avidity of the construct. Encouraged by these results, we transduced primary T cells from nine healthy donors with the indicated CAR construct (Figures 2C and 2D). In agreement with Okuno et al.,^20^ we observed that the viability of CD37CAR T cells decreased by 30%–40% 12 days after transduction (Figure 2E), affecting the expansion of the T cells (Figure 2F). Nonetheless, CAR T cell specificity and functionality were evaluated against target cell lines. Co-cultured CAR T cells displayed a specific CD107a degranulation marker upon target cell encounter. Importantly, CD37CAR reacted against CD37^high^ BL-41 and CD37^low^ U-937 but not against the U-937^*CD37KO*^. The controls, CD19CAR, and CD33CAR T cells were as potent as CD37CAR T cells against relevant targets, and all constructs activated both CD8 and CD4 T cell populations (Figures 2G and S8E). Activated CAR T cells produced inflammatory cytokines such as interferon (IFN)-γ, tumor necrosis factor (TNF)-α IL-2, and granulocyte-macrophage colony-stimulating factor (GM-CSF) following the same pattern of antigen specificity (Figures 2H and S9). Interestingly, some cytokines (G-CSF, IL-12, IL-15, IL-2, and IL-17) seemed to be produced by CD37CAR T cells in the presence of U-937^*CD37KO*^ or without target cells (E only), which probably reflects the tonicity of the construct (Figure S9). Finally, CAR T cell killing capacity was evaluated using bioluminescence (BLI)-based killing assays against different AML cell lines (Figure 2I, and see Figure S8C for target expression). Together, these assays demonstrate the potency and specificity of CD37CAR T cells against AML.

### CD37CAR T cells efficiently kill AML cells *ex vivo* and spare normal HSCs

**Figure 3:**
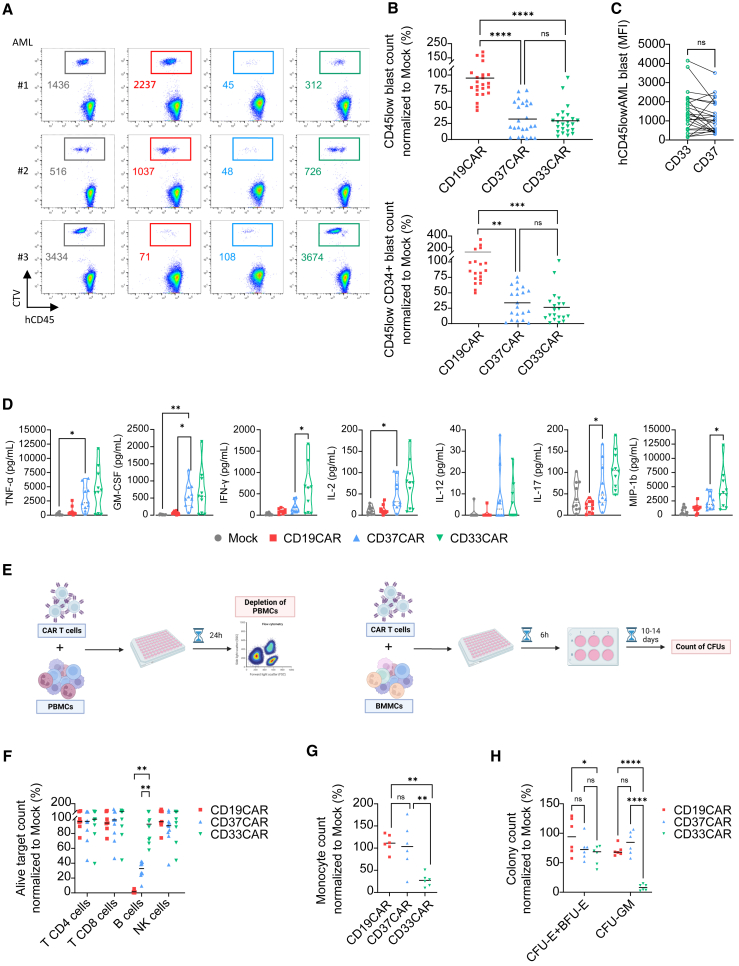
CD37CAR is as efficient but safer than CD33CAR (A) Flow cytometry-based, depletion killing assay of two AML(#1 and #2) and one B-ALL (#3) patients’ BMMCs labeled with CTV and co-cultured with T cells for 24 h at E:T = 5:1. Remining live cells are gated using color code corresponding to the construct expressed by T cells, mock = black, CD19CAR = red, CD37CAR = blue, and CD33CAR = green. Numbers are event count. (B) Same as in (A), counts of live AML patients’ CD45_low_ blasts (*n* = 24) and CD45_low_ CD34^+^ blasts (*n* = 20) normalized to mock (%) after 24 h of co-culture. One-way ANOVA followed by Tukey’s multiple comparisons tests, bars are mean, ∗∗*p* < 0.01, ∗∗∗*p* < 0.001, ∗∗∗∗*p* < 0.0001, ns = not significant. (C) Geometric median fluorescent intensity of CD33 and CD37 staining on AML patients (*n* = 24). Paired t test was used for statistical analysis, ns = not significant. (D) Cytokine secretion (pg/mL) of TNF-α, IFN-γ, GM-CSF, G-CSF, IL-2, IL-12, IL-15, IL-17, and MIP-1b in the supernatant of (A) and (B) after 24 h of co-culture. E:T = 1:2 (*n* = 2 T cell donors co-cultured with five AML samples each; mean). One-way ANOVA followed by Dunnett’s multiple comparisons tests. Only significant comparisons to CD37CAR are displayed, ∗*p* < 0.05, ∗∗ = *p* < 0.01. (E) Evaluation of CAR T cell toxicity toward healthy blood and hematopoiesis. Healthy donors’ PBMCs (labeled CTV) (right) and BMMCs (containing CD34^+^) (left) were co-incubated with autologous T cells. After 24 h of co-culture, PBMCs were then analyzed by flow cytometry for depletion killing. After 6 h of co-culture, BMMCs were further cultured for 10 days in a colony-forming unit (CFU) assay. (F) Counts of CD45^+^ CD3^+^ CD4^+^ T cells alive, CD45^+^ CD3^+^ CD8^+^ T cells, CD45^+^ CD19^+^ B cells, CD45^+^ CD3^−^ CD19^−^ CD56^+^ natural killer (NK) cells, and CD45^+^ CD14^+^ monocytes normalized to mock (%) after 24 h of co-culture (*n* = 7 donors for CD19CAR and *n* = 10 donors for CD37^−^and CD33CAR; mean). Bars are mean, two-way ANOVA followed by Tukey’s multiple comparisons tests. Only significant comparisons are displayed, ∗∗*p* < 0.01. (G) Counts of CD45^+^ CD11b^+^ monocytes alive normalized to mock (%) after 24 h of co-culture with CD19CAR, CD37CAR, and CD33CAR (*n* = 6 donors). Bars represent means, one-way ANOVA followed by Tukey’s multiple comparisons tests, ∗∗*p* < 0.01, ns = not significant. (H) CFU counts of erythroid colonies (CFU-erythroid and burst-forming unit-erythroid) and myeloid colonies (CFU-granulocyte macrophage) normalized to mock (%) (*n* = 2 donors, triplicates, mean). Two-way ANOVA followed by Tukey’s multiple comparisons tests, ∗*p* < 0.05, ∗∗∗∗*p* < 0.0001, ns = not significant.

We next tested the ability of CD37CAR T cells to kill primary AML samples. AML bone marrow mononuclear cells (BMMCs) were co-cultured with CAR T cells for 24 h. We observed that CD37^−^ and CD33CAR T cells, but not CD19CAR T cells, specifically killed AML blasts (Figures 3A and 3B). Of note, CD33 and CD37 expression was comparable in the challenged blast population (Figure 3C). We also evaluated the cytokines secreted in the co-culture supernatants. Since the BM might contain B cells, we cannot exclude that they contributed to the stimulation of CD19^−^ and CD37CAR T cells. Likewise, CD33 CAR T cells could be stimulated by myeloid cells (Figure 3D). Although less potent than CD33CAR, CD37CAR T cells secreted cytokines corresponding to a T helper 1 (Th1)-like profile with IFN-γ, TNF-α, and IL-2. Taken together, we showed that CD37CAR T cells depleted primary patient AML cells *ex vivo* and produced inflammatory cytokines.

Next, we evaluated CD37CAR toxicity toward terminally differentiated blood cell populations and HSPCs.^12^^,^^13^ Different autologous CAR T cells were generated and co-cultured with peripheral blood mononuclear cells (PBMCs) for 24 h or BMMCs for 6 h (Figures 3E–3G). BMMCs were further cultivated for 10 days in a hematopoietic colony-forming unit (CFU) assay. CD19^−^ and CD37CAR T cells depleted B cells (Figure 3F). In this assay, the effect on the monocyte population was difficult to capture (not shown); we thus run a similar co-culture assay where a larger number of monocytes was isolated and co-cultured with CAR T cells (Figure 3G). Here, CD33CAR T cells clearly reduced the monocyte population, whereas CD37CAR T cells had little or no effect and were significantly less toxic than CD33CAR T cells. We next evaluated the toxicity of the different CAR T against normal hematopoiesis and noticed that CD33CAR T cells displayed slight toxicity toward progenitors of erythroid origin (*p* < 0.05 vs. CD19CAR) while being highly toxic toward myeloid progenitors (Figure 3H; *p* < 0.001 vs. both CD19^−^ and CD37CAR). In comparison, CD37CAR T cells did not react toward either erythroid or myeloid progenitors (Figure 3H, not significant). We concluded that CD37CAR T cells are less myelotoxic than CD33CAR T cells.

### CD37CAR T cells control different AML models *in vivo*

**Figure 4:**
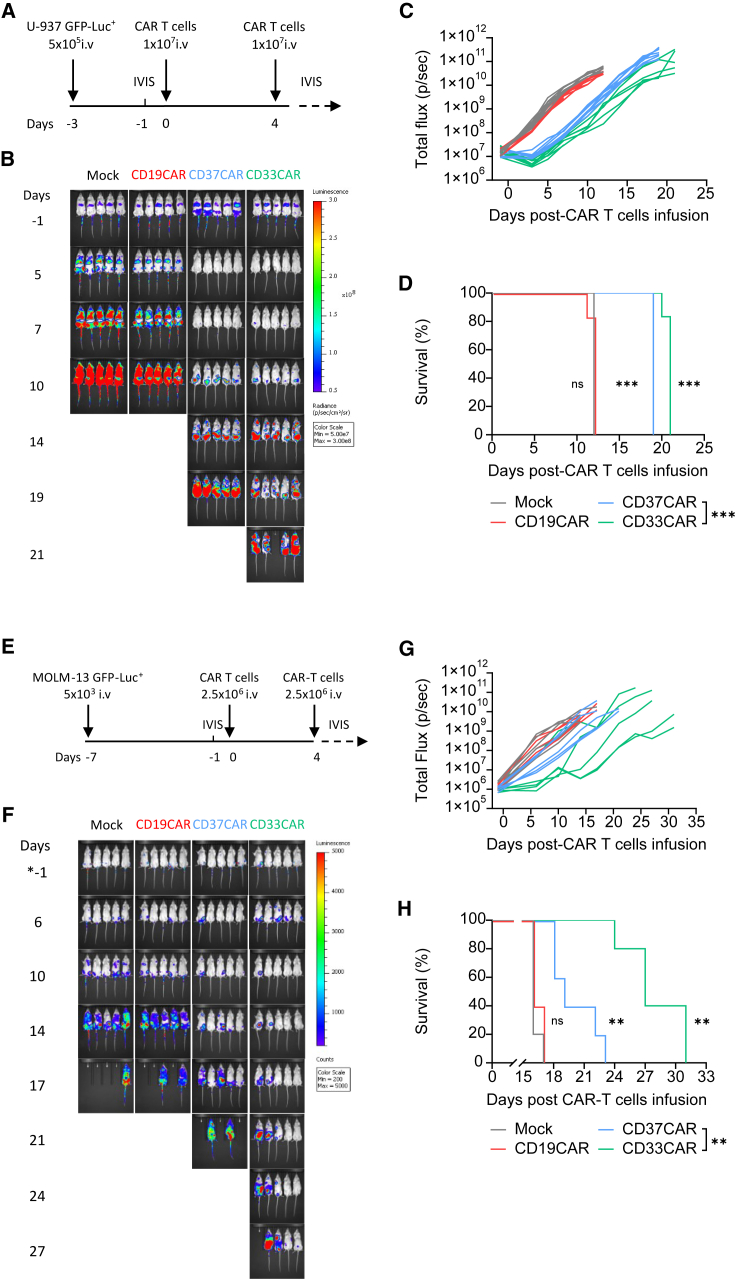
CD37CAR T cells have potent anti-AML activity *in vivo* (A) Schematic of the U-937-based *in vivo* experimental design. Three days before T cell injection, 5 × 10^5^ U-937 GFP-Luc^+^ cells were inoculated intravenously (i.v.) in NXG mice. *In vivo* imaging system (IVIS) was performed 1 day before T cell injection to confirm tumor establishment and randomize the mice. On day 0 and day 4, 1 × 10^7^ mock, CD19^−^, CD37^−^, or CD33CAR T cells were injected i.v. The percentage of CAR-expressing population was adjusted between the groups to 50% using mock cells. Tumor growth was tracked two times a week using IVIS. (B) Representative bioluminescence images. (C) Bioluminescence kinetics of U-937 GFP-Luc^+^ cells growth in NXG mice treated with CAR T cells (*n* = 6 mice per group). (D) Kaplan-Meier survival curves of NXG mice bearing U-937 GFP-Luc^+^ cells and treated with CAR T cells (*n* = 6 mice per group). Comparisons of survival curves were determined by log rank test. (E) Schematic of the MOLM-13-based *in vivo* experimental design. Seven days before T cell injection, 5 × 10^3^ MOLM-13 GFP-Luc^+^ cells were inoculated i.v. in NXG mice. IVIS was performed 1 day before T cell injection to confirm tumor establishment and randomize the mice. On day 0 and day 4, 2.5 × 10^6^ mock, CD19^−^, CD37^−^, or CD33CAR T cells were injected i.v. The percentage of the CAR-expressing population was adjusted between the groups to 40% using mock cells. Tumor growth was tracked two times a week using IVIS. (F) Representative bioluminescence images. (G) Bioluminescence kinetics of MOLM-13 GFP-Luc^+^ cells growth in NXG mice treated with CAR T cells (*n* = 5 mice per group). (H) Kaplan-Meier survival curves of NXG mice bearing MOLM-13 GFP-Luc^+^ cells and treated with CAR T cells (*n* = 5 mice per group). Comparisons of survival curves were determined by log rank test, ∗∗∗*p* < 0.001, ns = not significant.

To study the efficacy of the CD37 CAR *in vivo*, we injected intravenously (i.v.) a high-dose of CAR T cells (1 × 10^7^) into NOD xenograft gamma (NXG) mice that were challenged with U-937 cells expressing GFP-Luciferase^+^ (GFP-Luc^+^) (Figure 4A). While mock and CD19CAR T cells failed to control tumor burden, CD37^−^ and CD33CAR T cells showed anti-AML activity with prolonged survival (Figures 4B–4D, median survival 19–21 days versus 12 in control; *p* < 0.001). The sensitivity of CD37CAR T cells to antigen density was tested using a CD37^very low^ AML model, MOLM-13 (Figures S2C and S5C), *in vivo* (Figure 4E). Importantly, we observed with this aggressive model that CD37CAR T cells were still able to slow tumor progression (Figures 4E–4H; *p* < 0.01). In addition, in these two models, we observed that the CARs did not trigger any cytokine release syndrome based on the absence of weight loss in the animals (Figures S10A and S10B). Thus, CD37CAR is reactive against AML cell lines with a variable range of CD37 antigen density with no major side effects.

**Figure 5:**
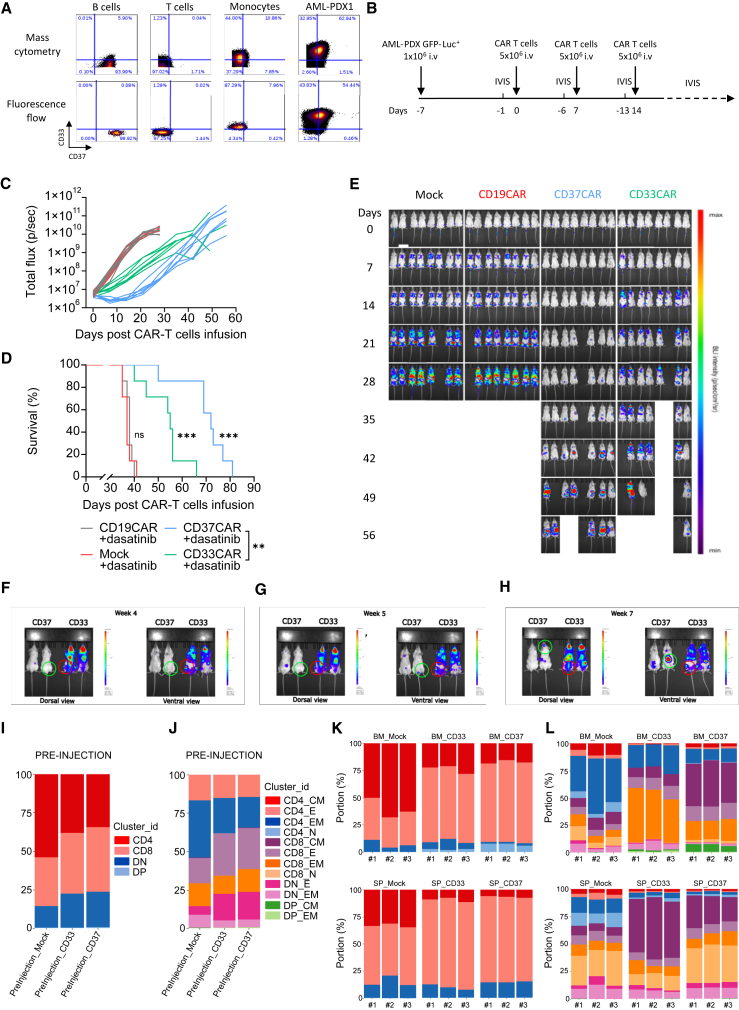
CD37CAR T cells control AML-PDX model (A) Detection of CD37 and CD33 in AML-PDX1 cells and healthy donor cells by mass cytometry and flow cytometry. (B) Schematic representation of the PDX *in vivo* experimental design. Seven days before the first T cell injection, 1 × 10^6^ AML-PDX GFP-Luc^+^ cells (F2) were inoculated i.v. in NOD scid gamma (NSG) mice. IVIS was performed 1 day before T cell injection to confirm tumor establishment and randomize the mice, and 5 × 10^6^ mock, CD19^−^, CD37^−^, or CD33CAR T cells expanded with dasatinib were injected i.v. on day 0, 7, and 14. The percentage of CAR-expressing population was adjusted between the groups to 50% using mock cells. Tumor growth was tracked weekly using IVIS for 6 weeks after T cell injection. (C) Bioluminescence kinetics of the AML-PDX GFP-Luc^+^ cells growth in NSG mice treated with CAR T cells (*n* = 7 mice per group). (D) Kaplan-Meier survival curves of NSG mice bearing AML-PDX GFP-Luc^+^ cells and treated with CAR T cells (*n* = 7 mice per group). Comparisons of survival curves were determined by log rank test, ∗∗∗*p* < 0.001, ns = not significant. (E) Representative bioluminescence images. (F) Representative bioluminescence images of close-up BM area of mice treated with CD33CAR or CD37CAR. Dorsal and ventral view comparing the bioluminescence at week 4. The green circle (CD37CAR-treated mice) and the red circle (CD33CAR-treated mice) emphasize the re-growth of AML cancer cells in the mice. (G) Same as in (F) at week 5. (H) Same as in (F) at week 7. (I) MC of group CAR T cell at pre-injection using level 1 depth characterization. Pool of three mice per group. (J) MC of group CAR T cell at pre-injection using level 2 depth characterization. Pool of three mice per group. (K) MC of CAR T cells from three mice per group (#1, #2, #3) at day 6 after CAR T injection, grouped by anatomical site using level 1 depth characterization. (L) MC of CAR T cells from three mice per group (#1, #2, #3) at day 6 after CAR T injection, grouped by anatomical site using level 2 depth characterization.

Our first attempt to evaluate CD37CAR T cells in a PDX model revealed that CD37CAR T cell fitness was probably not sufficient to control tumor growth in more complex models (Figures S11A–S11E). As previously shown, CD37CAR impeded T cell growth (Figure 2F). It was recently reported that the kinase inhibitor dasatinib could improve CAR T cell manufacturing,^46^^,^^47^ which we also observed with CD37CAR T cells (Figures S12A and S12B) as well as the killing of CD37 and CD33CAR T cells (Figure S12C). Remarkably, dasatinib seemed to dampen CD37CAR tonicity as reflected by the decrease of CD107^+^ CD8^+^ CD37CAR T cells at steady state (Figure S12D). Importantly, dasatinib did not affect mock or CD33CAR T cell expansion and phenotype (Figure S12). To evaluate the efficacy of CD37CAR T cells in a more relevant pre-clinical model, we engrafted mice with GFP-Luc^+^ AML patient-derived cells. As shown in Figure 5A, the AML patient-derived cells expressed CD33 and CD37 antigen. In comparison to control groups, CD37CAR T cells delayed AML growth (Figures 5B, 5C, and 5E) and extended survival of the animals (Figure 5D; *p* < 0.001). Strikingly, the dasatinib-expanded CD37CAR T cells outperformed dasatinib-expanded CD33CAR T cells and demonstrated a significantly improved median survival of 55 vs. 74 days (Figure 5D). Interestingly, the pattern of disease recurrence was different between CD37CAR and CD33CAR; in contrast to CD33CAR, the recurrence of cancer cells in mice treated with CD37CAR did not originate from expected sites such as spine or femur BM (Figures 5F and 5G, green circle) of the mice, but from skull or liver localized cells (Figure 5H, green circle). In mice treated with CD33CAR, control of disease progression was lost on day 14 post CAR T injection (Figure 5E), and disease relapse could be detected in the femur (Figures 5F, G and H, red circle) and in the spinal cord (Figure 5H, red circle). Notably, for both CD33^−^- and CD37CAR T cell-treated mice, the recurrence of cancer cells in the PDX model was not due to antigen loss (Figure S13). These data suggest that CD37CAR has superior pre-clinical efficacy than the current anti-AML CD33CAR. Importantly, no weight loss in the animals was observed, suggesting that neither CD33 nor CD37CAR triggered cytokine release syndrome (Figures S10C and S10D).

To follow the differentiation of the CAR T cells *in vivo*, we undertook a comprehensive characterization of the CAR T cell population during the treatment. T cell populations were meta-clustered by using two different levels of classifications (level 1 and level 2) based on the selection of antigens described in Table S4. As shown in Figures 5I and 5J, we compared the T cell phenotype of the different CAR T cell groups (i.e., mock, CD33, and CD37) before injection. The distribution of the CD4, CD8, double-negative (DN), and double-positive (DP) T cell populations was similar between the CD33 and CD37CAR T cells (Figure 5I). Interestingly, the distribution was slightly different for the mock group. This could reflect an effect of the CD33 and CD37CAR-T construct expression on the biology of the T cells during the activation and expansion phase of the CAR T product preparation. Using a larger panel of markers (level 2) to identify more in depth the T cell populations, we confirmed that the CD33^−^ and CD37CAR-T products were very similar, in terms of T cell subtype population distribution, at the time of injection.

As shown in Figures 5K and 5L, we explored the differentiation phenotype of T cells during CAR T cell therapy. To align with prior studies on T cell differentiation following infection,^48^ mice were sacrificed on day 6. We observed distinct distributions of the CAR T cell phenotype among the groups (Figures 5K–5L). Notably, there was an increased proportion of CD8 CM T cells in the BM of CD37CAR T-treated mice compared to CD33CAR-T cells and mock-treated mice. Conversely, the spleen of CD33 CAR T-treated mice exhibited a higher proportion of CD8 CM T cells compared to the one of CD37 CAR T-treated group. Overall, our observations highlight CAR T construct and organ dependencies in T cell phenotype and differentiation. Notably, in our experimental setup, the absence of disease recurrence in the BM of CD37 CAR T-treated mice, as opposed to CD33 CAR T-treated mice, may be attributed to the presence of more CD8 CM T cells in their BM.

## Discussion

In the present study, we validated the utilization of CD37 as a target for a safe CAR T cell therapy approach to treat AML. CD37 was detected by flow and mass cytometry on the surface of different AML cell lines and primary samples. This was supported by RNA-seq analysis showing that, although *CD37* mRNA was more broadly detected, it was enriched in AML samples. Because the HH1 anti-CD37 antibody efficiently detected CD37 on AML samples, we tested the efficacy of HH1-based CD37CAR in different pre-clinical experiments. We compared it to the clinically tested CD33CAR and observed a similar anti-AML activity but, unlike CD33CAR, without toxicity against healthy tissues.

Antibody- and CAR-based therapies rely on restricted surface expression of their target. CD37 previously appeared as an ideal therapeutic target: it is a lineage marker of mature B cells also highly expressed in B cell malignancies, yet its presence was detected at a much lower level in monocytes^22^^,^^23^ and activated T cells.^20^ Still, this did not seem to lead to any toxic effects since therapeutic anti-CD37 antibodies were reported to be safe.^49^^,^^50^ Our group and others have recently developed a CD37-targeting CAR T cell-based therapy^20^^,^^31^^,^^51^ mirroring the CD19CAR T cell approach described for the treatment of B cell leukemia, B cell lymphoma,^26^^,^^29^ and T cell lymphoma.^31^ We established that the *CD37* mRNA was overexpressed in a wide range of AML patients’ samples, especially in the adverse risk AML group, such as t(11q23)/MLL AML, indicating that this AML population could potentially benefit from a CAR therapy targeting CD37. Because gene expression profile analysis may not fully predict protein expression, we investigated the presence of CD37 on AML cell lines by flow cytometry and on 59 AML patient samples by mass cytometry. The CD37 protein expression on the AML cells significantly correlated with the ELN 2017 patient risk stratification. A recent study^52^ has linked CD37 protein to fatty acid metabolism to aggressive B cell lymphoma. It is tempting to speculate that AML cells, which are dependent on long-chain fatty acid metabolism for their survival,^53^ might also require CD37 to increase their fitness. Thus, the increased presence of CD37 would follow the resistance to standard treatments by enhancing their metabolic efficiency. Our results are in accordance with previous findings, using immunohistochemistry and flow cytometry-based approaches, showing that AML cells overexpressed CD37 at the protein level compared to healthy CD34^+^ cells and that the density on AML LSCs is equal to, if not higher than, that of AML myeloblasts.^24^ We sought to validate our target by rationalizing its expression in comparison to the pan-myeloid CD33 marker and the CD123 stem cell marker currently evaluated in CAR clinical trials for AML.^7^ We observed an equivalent frequency of expression of around 80% of the primary blasts expressing the three proteins regardless of the prognosis factor and CD34 expression. This provides evidence that CD37 is expressed at the cell surface and represents a rational biomarker in term of expression, equivalent to CD33 and CD123, for the characterization of AML cells. In contrast to CD37, the expression of CD33 did not correlate with the AML subtypes or ELN risk stratification. Hence, unlike CD33 and CD123 showing broader expression outside the hematopoietic system,^54^ CD37 distribution seems to be restricted to terminally differentiated blood cells,^21^^,^^22^ making CD37 an attractive target for cell therapy in AML.

The relapsed and refractory forms of AML are in part presumably due to the presence of a persisting population of LSCs that is resistant to chemotherapy and replenishes the pool of AML blasts.^9^ Therefore, assessing the presence of CD37 in these cells is of great interest to confirm that a CD37-based CAR T therapy can eradicate the disease at its roots. Previous studies have confirmed the expression of CD33 and CD123 in R/R AML, including LSCs.^54^ Here, we described that CD37 expression can be detected on 70%–80% of both GMP- and LMPP-like LSC patient blasts. The observation that these two LSC populations coexist in 80% of patients^9^ and that the primitive LMPP-like LSCs are found enriched in recurrent AML^55^ warrants clinical investigation of CD37 CAR T cells in AML treatment.

Remarkably, the detection of CD37 was not straightforward and, by using different commercial antibodies, we unexpectedly noticed discrepancies in staining of CD37 in AML that were not apparent in B cells. We anticipated that different splice variants could be involved in this cell-type recognition, but we did not identify any differences. AML patients have not been reported to carry CD37 mutations and the patient samples used in our study did not have detectable CD37 mutations. However, despite the fact that AML could be characterized as a disease with low tumor mutational burden, more extensive analysis could be undertaken to exclude the presence of CD37 mutations in AML patients. The sensitive of the anti-CD37 antibody could be affected by the cell-surface antigen density or post-translational modifications such as glycosylation.^17^^,^^23^ In fact, aberrant glycosylation is considered a hallmark of cancer and it has been exploited to develop glycoantigen-specific antibodies and CARs.^56^ However, using an enzyme removing sialic acid groups, a common modification of glycosylated proteins in cancer, did not alter HH1 antibody and CAR recognition of AML cells. Accurately determining the HH1 dependency on glycosylation will require deeper structural analysis. Regardless, the HH1-based CAR construct conserved its ability to recognize CD37 in the context of AML.

Although CD37 levels on AML are low compared to B cell malignancies, we clearly demonstrate that AML can efficiently be targeted using HH1 antibody-derived CD37CAR. We further show that this CD37CAR was as efficient as, or superior to, CD33CAR in several models. The CD37 target carries the advantage of low expression in healthy tissues except in mature B cells. Moreover, it is important to note that the CD37CAR T cells will only eliminate terminally differentiated B cells and leave a naive population. Thus, in a clinical setting, B cell aplasia might be expected but will be manageable.^57^ None of the AML CAR antigens investigated so far are exclusively expressed on AML cells and often share expression with healthy HSPCs or other non-hematopoietic tissues.^7^^,^^54^For example, although successful in pre-clinical studies, CD33^−^ and CD123CAR T cells cause long-term myeloablation, which can lead to serious toxicity issues.^11^^,^^12^ A few other CARs targeting early developmental markers have been shown to spare myeloid cells.^58^^,^^59^ However, one of these targets, CD70, is expressed on activated lymphocytes and subsets of dendritic cells, and another, Siglec-6, is expressed on mast cells and basophils.^60^On the other hand, CD37CAR T cells have shown no toxicity and no inflammatory cytokine production against differentiated cell lineages other than B cells.^20^^,^^31^^,^^32^ We also observed that CD37CAR T cells showed superior ability to control disease recurrence in the BM in comparison to CD33CAR T cells. Interestingly, our phenotypic characterization demonstrated CAR T cell differentiation *in vivo* to be organ dependent. Indeed, we showed that the CD33 and CD37CAR T cell products were nearly identical at the level of T cell subpopulation distribution before injection. However, after 6 days, the distribution of the different T cell populations was clearly distinct between the CD33^−^ and CD37CAR T cells. Interestingly, we could observe a higher proportion of CD8 CM T cells in the BM of animals treated with CD37CAR T cells when compared to mice treated with the CD33CAR and mock T cells. In addition, we observed that disease recurrence never occurred in the BM of the mice treated with CD37CAR T cells, which contrasts with the other groups. This might be linked to the increased presence of CD8 CM T cells in the BM of the CD37CAR T cell group and the ability of these cells to drive a more potent T cell differentiation that will result in improved control of the AML. Previous studies investigating T cell differentiation after infection have connected it to antigen recognition, directly shaped by the functional avidity of the T cell receptor (TCR) for its target.^48^^,^^61^^,^^62^^,^^63^ It is tempting to speculate that the differentiation spectrum of the CAR T cells and the performance of the different CAR T cell products is linked to its functional avidity, which might differ between CD37CAR and CD33CAR.^48^ Even if our experimental setup cannot fully address this question, our study detected a striking modification in the differentiation profile of the CAR groups *in vivo*. Nonetheless, our investigation has highlighted that CAR T cell differentiation *in situ* depends on the single-chain variable fragment (scFv) and/or the antigen density on the targeted organ.

Interestingly, Okuno et al.^20^ reported that a short and transient expression of CD37 during T cell activation can affect T cell expansion. The authors suggested that the lower efficacy in T cell expansion was reminiscent of T cell fratricide killing. However, fratricide was never experimentally demonstrated for CD37CAR T cells and we rather attribute this lack of expansion to toxicity caused by the strong tonic signaling (Forcados, Wälchli, et al., unpublished data). In line with this idea, chronic toxic signaling was also reported with CAR targeting GRP78, which would be induced by the encounter of the CAR molecule and the target within the T cell. Intriguingly, the authors resolved the tonic toxicity issue by inhibiting endogenous CAR activation during CAR T cell manufacturing using the kinase inhibitor dasatinib.^46^ The efficacy of dasatinib at preventing tonic signaling was further confirmed in a study using a CAR targeting CD7.^64^ Our results also show that the presence of dasatinib during CD37CAR T cell manufacturing can restore T cell expansion without affecting their functional capacities.

In summary, CD37 is an attractive CAR target. It is widely expressed at the surface of primary AML cells, including LSCs. Moreover, we uncover that the expression of CD37 in myeloid cells is associated with ELN 2017 risk stratification and thus is related to adverse outcome and poor survival. In addition, CD37 biodistribution predicts lower on-target toxicity over the other AML CAR targets. Our *in vitro* and *in vivo* studies highlight the potent anti-AML activity of HH1-based CD37CAR, which is also able to spare normal myeloid cells and stem cells. This is an important feature and, in several cases, the targets are genetically manipulated by disruption^65^ or base correction^66^ to circumvent myeloid and stem cell toxicity. Thus, our findings strongly warrant clinical investigation of CD37CAR to treat AML patients.

### Limitations of the study

Although we present *in vivo* data from different AML cell lines and PDX, experiments with lower number of CAR T cell injections would provide additional insight on the robustness of CD37CAR T cells to treat AML in more physiological conditions. Furthermore, studying long-term T cell persistence in animals could support prediction of the clinical CAR product efficacy, but human T cells in these model systems often cause xenogeneic graft-versus-host disease (GvHD), hampering long-term monitoring. We focused our study on the presented 4-1BB-containing CD37CAR design; however, alternative signaling tails could be compared to identify the most efficient anti-AML CD37CAR.

## STAR★Methods

### Key resources table

**Table:** 

REAGENT or RESOURCE	SOURCE	IDENTIFIER
**Antibodies**		

CD3-BV421(SK7)	BD Biosciences	Cat. No.563798; RRID:AB_2744383
CD3-BV605 (SK7)	BD Biosciences	Cat. No.563219; RRID:AB_2714001
CD4-BV421(RPA-T4)	BD Biosciences	Cat. No.562842; RRID:AB_2737832
CD4-BV605 (RPA-T4)	BD Biosciences	Cat. No.562658; RRID:AB_2744420
CD8-BV605 (RPA-T8)	BioLegend	Cat. No.301040; RRID:AB_2563185
CD8-PE-Cy7 (RPA-T8)	Thermo Fisher Scientific	Cat. No. 25-0088-42; RRID:AB_1659702
CD11b-PE (ICRF44)	BD Biosciences	Cat. No.555388; RRID:AB_395789
CD14-APC-Cy7 (MφP9)	BD Biosciences	Cat. No.557831; RRID:AB_396889
CD16-FITC (eBioCB16)	Thermo Fisher Scientific	11-0168-42; RRID:AB_10805747
CD19-BV421(HIB19)	BD Biosciences	Cat. No.562440; RRID:AB_11153299
CD19-PE (HIB19)	Thermo Fisher Scientific	Cat. No.12-0199-42; RRID:AB_1834376
CD20-APC	Thermo Fisher Scientific	Cat. No.17-0209-42; RRID:AB_10670628
CD33-BV421 (WM53)	BD Biosciences	Cat. No.562854; RRID:AB_2737405
CD34-PE-Cy7 (581)	BD Biosciences	Cat. No.560710; RRID:AB_1727470
CD34-APC (4H11)	Thermo Fisher Scientific	Cat. No.17-0349-42; RRID: AB_2016672
CD37-Af647 (M-B371)	BD Biosciences	Cat. No.561562; RRID:AB_10895803
CD37-Af647 (HH1)	Santa Cruz Biotechnology	Cat. No.sc-18881 AF647
Unconjugated anti-CD37 (HH1)	Santa-Cruz Biotechnology	Cat. No.sc-18881
CD38-APC-R7 (HIT2)	BD Biosciences	Cat. No.564979; RRID:AB_2744373
CD45-FITC (HI30)	BD Biosciences	Cat. No.555482; RRID:AB_395874
CD45RA-BV510 (HI100)	BD Biosciences	Cat. No.563031; RRID:AB_2722499
CD45RA- BB515 (HI100)	BD Biosciences	Cat. No.564552; RRID:AB_2738841
CD56-APC (NCAM16.2)	Thermo Fisher Scientific	Cat. No.17-0566-42; RRID:AB_2573148
CD69-PE-Cy5 (FN50)	BD Biosciences	Cat. No.555532; RRID: AB_395917
CD107a-PE-Cy5 (H4A3)	BD Biosciences	Cat. No.555802; RRID: AB_396136
CD107 APC (H4A3)	BD Biosciences	Cat. No.560664; RRID:AB_1727417
CCR7 (CD197)-FITC (150503)	BD Biosciences	Cat. No.561271; RRID:AB_10561679
CD90-BV605 (5E10)	BD Biosciences	Cat. No.747750; RRID:AB_2872219
CD123-PE (7G3)	BD Biosciences	Cat. No.554529; RRID:AB_395457
CD123- PerCP-Cy™5.5 (7G3)	BD Biosciences	Cat. No.558714; RRID:AB_1645547
Anti-Human HLA-DR (L243) - 116Cd	BioLegend	Cat. No. 307651
Anti-Human Lineage Cocktail 2 (lin 2) *(CD3, CD14, CD19, CD20, CD56)*	BD Biosciences	Cat. No.643397
Murine IgG1 Isotype – Af647 (MOPC-21)	BioLegend	Cat. No.400136; RRID:AB_2832978
Mouse IgG1 Isotype –FITC (MOPC-21)	BD Biosciences	Cat. No.554679; RRID: AB_395505
Biotin-SP (long spacer) AffiniPure™ F(ab')₂ Fragment Goat Anti-Mouse IgG, F(ab')₂ fragment specific	Jackson Immuno Research Laboratories	Cat. No.115-066-072; RRID: AB_2338583
Streptavidin-PE	BD Biosciences	Cat. No.554061; RRID:AB_10053328
Anti-HA tag antibody (mAb clone 2–2.2.14)	Invitrogen	Cat. No.26183; RRID:AB_10978021
Anti-CD37 antibody (mAb clone E4K2M)	Cell Signaling Technology	Cat. No.46894;
Goat anti-rabbit IgG antibody conjugated to horseradish peroxidase	Invitrogen	Cat. No.31460; RRID:AB_228341
anti-CD3 (OKT3), Biotin	Thermo Fisher Scientific	Cat. No.13-0037-82; RRID:AB_1234955
anti-CD28 (CD28.6), Biotin	Thermo Fisher Scientific	Cat. No.13-0289-82; RRID:AB_466415
Anti Biotin (1D4-C5) - 143ND	Standard BioTools	Cat. No.3143008B
Anti-Human CD3 (UCHT1) – 170Er	Standard BioTools	Cat. No.3170001B
Anti-Human CD3 (UCHT1) – 111Cd	BioLegend	Cat. No.300443
Anti-Human CD4 (RPA-T4) −145ND	BioLegend	Cat. No.300541
Anti-Human CD7 (CD7-687) −114Cd	BioLegend	Cat. No.343102
Anti-Human CD8 (HIT8a) – 139La	BioLegend	Cat. No.300902
Anti-Human CD11b (ICRF44) - 209Bi	Standard BioTools	Cat. No.3209003B
Anti-Human CD11c (L243) – 174Yb	BioLegend	Cat. No.337221
Anti-Human CD14 (M5E2) −160Gd	Fluidigm	Cat. No.3160001B
Anti-Human CD16 (3G8) - 148ND	Fluidigm	Cat. No. 3148004B
Anti-Human CD19 (HIB19) - 165Ho	Standard BioTools	Cat. No.3165025B
Anti-Human CD19 (HIB19) – 141Pr	BioLegend	Cat. No. B318939
Anti-Human CD25 (2A3) - 169Tm	Standard BioTools	Cat. No.3169003B
Anti-Human CD27 (L128) - 162Dy	Standard BioTools	Cat. No.3162009B
Anti-Human CD28 (CD28.2) - 160Gd	Standard BioTools	Cat. No.3160003B
Anti-Human CD33 (WM53) - 158Gd	Standard BioTools	Cat. No.3158001B
Anti-Human CD34 (4H11) – 145ND	eBioscience	Cat. No.15236917
Anti-Human CD34 (581) - 113Cd	BioLegend	Cat. No. 343531
Anti-Human CD38 (HIT2) - 144ND	Standard BioTools	Cat. No.3144014B
Anti-Human CD38 (HIT2) −144ND	Standard BioTools	Cat. No.3144014C
Anti-Human CD44(BJ18) −166Er	Fluidigm	Cat. No.2103505-29
Anti-Human/Mouse CD44 (IM7) - 171Yb	Standard BioTools	Cat. No.3171003B
Anti-Human CD45 (HI30) - 89Y	Standard BioTools	Cat. No.3089003B
Anti-Mouse CD45 (30-F11) - 147Sm	Standard BioTools	Cat. No.3147003B
Anti-Human CD45RA (Hi100)	Fluidigm	Cat. No.3143006B
Anti-Human CD45RO (UCHL1) - 149Sm	Standard BioTools	Cat. No.3149001B
Anti-Human CD45RA-BV510 (HI100)	BD Biosciences	Cat. No.563031
Anti-Human CD45RA-FITC (HI100)	BD Biosciences	Cat. No.561882
Anti-Human CD45RA (HI100) - 153Eu	Standard BioTools	Cat. No.3153001B
Anti-Human CD56-APC (NCAM16.2)	BD Biosciences	Cat. No.341025
Anti-Human CD56 (B159) −155Gd	Standard BioTools	Cat. No.3155008B
Anti-Human CD57 (HNK-1)	BioLegend	Cat. No.359602
Anti-Human CD64 (10,1) – 146ND	Standard BioTools	Cat. No.3146006C
Anti-Human CD66b (8OH3) −152Sm	Fluidigm	Cat. No.3152011B
Anti-Human CD73(AD2) −172Yb	Abcam	Cat. No.ab130451
Anti-Human CD90 (5E10) −159Tb	Fluidigm	Cat. No.3159007C
Anti-Human CD95/Fas (DX2) - 152Sm	Standard BioTools	Cat. No.3152017B
Anti-Human CD105 (43A3) 163Dy	Fluidigm	Cat. No.3163005C
Anti-Human CD117/cKit (YB5B8) −168Er	Invitrogen	Cat. No. 14-1179-82
Anti-Human CD123 (6H6) – 112Cd	BioLegend	Cat. No 306027
Anti-Human CD123 (6H6) - 151Eu	Standard BioTools	Cat. No.3151001B
Anti-Human CD127 (A019D5) - 168Er	Standard BioTools	Cat. No.3168017B
Anti-Human CD134/OX40 (ACT35) - 142ND	Standard BioTools	Cat. No.3142018B
Anti-Human CD137/4-1BB (4B4-1) - 173Yb	Standard BioTools	Cat. No.3173015B
Anti-Human CD152/CTLA-4 (14D3) - 161Dy	Standard BioTools	Cat. No.3161004B
Anti-Human CD184/CXCR4 (12G5) - 175Lu	Standard BioTools	Cat. No.3175001B
Anti-Human CD185/CXCR5 (RF8B2) - 164Dy	Standard BioTools	Cat. No.3164029B
Anti-Human CD196/CCR6 (G034E3) - 141Pr	Standard BioTools	Cat. No.3141003A
Anti-Human CD197/CCR7 (G043H7) - 167Er	Standard BioTools	Cat. No.3167009A
Anti-Human CD223/LAG-3 (11C3C65) - 150ND	Standard BioTools	Cat. No.3150030B
Anti- Human/Mouse/Rat CD278/ICOS (C398.4A) - 148ND	Standard BioTools	Cat. No.3148019B
Anti-Human CD279/PD-1 (EH12.2H7) - 155Gd	Standard BioTools	Cat. No.3155009B
Anti-Human CD300e (233810) −173Yb	R&D	Cat. No.MAB2705
Anti-Human CD366/TIM-3 (F38-2E2) - 154Sm	Standard BioTools	Cat. No.3154010B
Anti-Human FLT3 (S18)-161Dy	BioLegend	Cat. No. 313302
Anti-Human Caspase 3(Cleaved) (D3E9)	Fluidigm	Cat. No. 3142004C
Anti-Human CSF1R (9-4D2-1E4) −170Er	BioLegend	Cat. No. 347302
AntiHuman Cyclin B1 (GNS-1)-164Dy	Fluidigm	Cat. No. 3165011B
Anti-GFP (FM2-64G)	BioLegend	Cat. No.338002
Anti-Human Histone H3 (D1H2) - 176Yb	Standard BioTools	Cat. No.3176016A
Anti-Human HLA-DR (L243) - 174Yb	Standard BioTools	Cat. No.3174001B
Anti-Human Ki-67 (B56) - 172Yb	Standard BioTools	Cat. No.3172024B
Anti-Human NRAS Q61 mut (EPR20278) – 154ND	Abcam	Cat. No.ab242415
Anti-Human pERK ½ [T202/Y204] (D13.14.4) −167Er	Fluidigm	Cat. No.3167005C
Anti-Human pRB(S807/811)(J112-906) – 150ND	Fluidigm	Cat. No.3150013A
Anti-Human pStat5 (Y694)/47) −147 Nd	Fluidigm	Cat. No.3150005A
Anti-Human RUNX1, RUNX2 and RUNX3 (EPR3099) 153Eu	Abcam	Cat. No.ab220117
Anti-Human TIGIT (MBSA43) - 159Tb	Standard BioTools	Cat. No.3159038B

**Bacterial and virus strains**		

NEB® 5-alpha Competent E. coli (High Efficiency) | DH5α	New England Biolab	C2987H

**Biological samples**		

Human peripheral blood mononuclear cells (PBMCs)	Healthy donors	(REK vest 2012/2247)
Peripheral blood samples	AML patients	(REK VEST 2015/1759)
AML biobank	AML patients	(REK 2022/48847), REK VEST (REK III nr. 060.02 and 059.02)) and NDPA 02/118-5
Bone marrow samples	AML patients	(2015/1012/REK sør-øst D)
Bone marrow with matched-blood samples	Healthy donors at bone marrow donation	N/A
AML patient-derived BMMCs	AML patients	N/A
Patient-derived xenografts (PDX)	AML patients	(FOTS ID 29646)

**Chemicals, peptides, and recombinant proteins**		

Fetal bovine serum (FBS)	Gibco	Cat. No.10500-064
Human serum	PAN Biotech	Cat. No.P40-2702HI
Human serum albumin	Octa pharma	N/A
Recombinant human IL-2 (Proleukin)	Clinigen	N/A
Gentamycin	Gibco	Cat. No.15750-037
BD Fc Block	BD Biosciences	Cat. No.564220
Deoxyribonuclease (DNAse) I from bovine pancreas	Sigma Aldrich	Cat. No.DN25
RIPA buffer	Thermo Fisher Scientific	Cat. No.89900
β-mercaptoethanol	Sigma Aldrich	Cat. No.M3148
Retronectin	Takara Bio.	Cat. No.T100B
Dasatinib	LC Laboratories	Cat. No.D-3307
Neuraminidase	Roche	Cat. No. 11585886001
Puromycin	Gibco	Cat. No.A1113802
Accutase	Thermo Fisher Scientific	Cat. No.A1110501
GolgiStop	BD Biosciences	Cat. No.554724
GolgiPlug	BD Biosciences	Cat. No.555029
D-Luciferin potassium salt	Revvity	Cat. No.122799
CFSE	Thermo Fisher Scientific	Cat. No.C34554
Cell Trace Violet	Thermo Fisher Scientific	Cat. No.C34557
Propidium Iodide	Thermo Fisher Scientific	Cat. No.R37169
Count Bright™ Absolute Counting Beads	Thermo Fisher Scientific	Cat. No.C36950
Stable-Lyse V2 buffer	Smart Tube, Inc.	STBLYSE2-250
Stable-Store V2 buffer	Smart Tube, Inc.	STBLSTORE2-1000
FcR blocking reagent, human	Miltenyi Biotec	Cat.No.130-059-901
CD16/CD32 Monoclonal Antibody (93) (FcR block)	eBioscience	Cat.No.15288387
MaxPar Cell Acquisition Solution Plus	Standard Bio-Tools	Cat.No. 201244
MaxPar phosphate-buffered saline (PBS)	Standard BioTools	Cat.No. 201058
MaxPar Cell Staining Buffer (CSB)	Standard BioTools	Cat.No. 201068
Paraformaldehyde (PFA)	Alfa Aesar	Cat.No. 43368
Dimethyl sulfoxide (DMSO)	Sigma Aldrich	Cat.No.D5879
Heparin-Natrium-5000 - Ratiopharm®	Ratiopharmn GmbH	N68743.08
Methanol	Sigma Aldrich	Cat.No. 32213-M
Phytohemagglutinin	Sigma Aldrich	Cat.No. 61764
Cell-ID™ Intercalator-Ir - 500 μM	Standard BioTools	Cat.No. 201192B
EQ™ Six Element Calibration Beads	Standard BioTools	Cat.No. 201245
Maxpar MCP9 Antibody Labeling Kit, 111Cd–4 Rxn	Standard BioTools	Cat.No. 201111A
Maxpar MCP9 Antibody Labeling Kit, 113Cd–4 Rxn	Standard BioTools	Cat.No. 201113A
Maxpar MCP9 Antibody Labeling Kit, 166Cd–4 Rxn	Standard BioTools	Cat.No. 201116A
Maxpar® X8 Antibody Labeling Kit, 145Nd—4 Rxn	Standard BioTools	Cat.No. 201145A
Maxpar® X8 Antibody Labeling Kit, 146Nd—4 Rxn	Standard BioTools	Cat.No. 201146A
Maxpar® X8 Antibody Labeling Kit, 156Gd—4 Rxn	Standard BioTools	Cat.No. 201156A
Maxpar® X8 Antibody Labeling Kit, 156Gd—4 Rxn	Standard BioTools	Cat.No. 201156A

**Critical commercial assays**		

QIFIKIT®, Series of coated beads, Flow Cytometry, 10 calibrations	Agilent DGG Norge AS	Cat. No.K007811-8
Cell-ID 20-Plex Palladium Barcoding Kit	Standard BioTools	Cat. No.201060
Bio-Plex Pro Human Cytokine 17-plex assay	Bio-rad	Cat. No. M5000031YV
Pan Monocyte Isolation Kit human	Miltenyi Biotec	Cat. No. 130-096-537

**Deposited data**		

Raw data “CD37 a safe Chimeric Antigen Receptor target to treat acute myeloid leukemia”	Mendeley Data	https://doi.org/10.17632/rdkg26mfjw.1

**Experimental models: Cell lines**		

HEK-P (Phoenix-AMPHO)	LCG Genomics GMBH	ATCC-CRL-3213
BL-41	Leibniz Institute DSMZ-German Collection	ACC 160
GRANTA-519	J. Myklebust (Oslo University Hospital)	PubMed: 30979721
Daudi	J. Myklebust (Oslo University Hospital)	PubMed: 30979721
K-562	ATCC	CCL-243
MOLM-13	Leibniz Institute DSMZ-German Collection	ACC 554
SKM-1	Leibniz Institute DSMZ-German Collection	ACC 547
HL-60	Leibniz Institute DSMZ-German Collection	ACC 3
U-937	Sigma-Aldrich Norway AS	85011440-1VL
MV4-11	ATCC	CRL-9591
HEL	Leibniz Institute DSMZ-German Collection	ACC 11
Jurkat76 (J76)	M. Heemskerk (Leiden University Medical Center, The Netherlands)	PubMed: 12869497

**Experimental models: Organisms/strains**		

NOD-*Prkdc*^*scid*^*-IL2rg*^*Tm1*^/Rj (NXG) mice	The Jackson Laboratory	SCANBUR AS

**Software and algorithms**		

GraphPad Software 8.0.2	GraphPad software	https://www.graphpad.com
FlowJo 10.7.1	FlowJo, LLC	https://www.flowjo.com/
BioRender	BioRender	https://www.biorender.com/
Python 3		N/A
Cytobank	Beckman Coulter	https://premium.cytobank.org/
R 4.2.0	R Core Team	https://www.r-project.org/
RStudio 2022.07.1 + 554	Posit	https://posit.co/products/open-source/rstudio/
CyTOF XT mass cytometer software 8.1.0 + 18524	Standard BioTools	N/A
IVIS®-200 imaging system	Perkin Elmer	https://www.perkinelmer.com/
MATLAB R2013a	MathWorks	https://www.mathworks.com/products/matlab.html
MATLAB application - single-cell debarcoder	Zunder et al.^67^	https://doi.org/10.1038/nprot.2015.020
R package - cytoBatchNorm 0.0.0.9001	GitHub/i-cyto	https://github.com/i-cyto/cytoBatchNorm
R package - CATALYST 1.22.0	GitHub/CATALYST	https://github.com/HelenaLC/CATALYST
R package - diffcyt 1.18.0	GitHub/lmweber^68^	https://github.com/lmweber/diffcyt
R package - flowCore 2.10.0	Bioconductor	https://www.bioconductor.org/packages/release/bioc/html/flowCore.html
R package - FlowSOM 2.6.0	Van Gassen S et al.^69^	https://bioconductor.org/packages/release/bioc/html/FlowSOM.html
R package - Premessa 0.3.4	GitHub/Parker Institute for Cancer Immunotherapy	https://github.com/ParkerICI/premessa

### Resource availability

#### Lead contact

Requests for further information and reagents should be directed to and will be fulfilled by the Lead Contact, Sébastien Wälchli (sebastw@rr-research.no).

#### Materials availability

Materials created in this study will be available for the scientific community by contacting the corresponding author and completion of a material transfer agreement.

#### Data and code availability

• Raw data have been deposited at Mendeley repository and are publicly available as of the date of publication. DOIs are listed in the key resources table.
• This paper does not report original code.
• Any additional information required to reanalyze the data reported in this work paper is available from the lead contact upon request.

### Experimental model and study participant details

#### Cell lines

The human cell lines HEK-P, BL-41, GRANTA-519, Daudi, K-562, MOLM-13, SKM-1, HL-60, U-937, MV4-11, and HEL were obtained from DSMZ and ATCC. The Jurkat76 was a kind gift from M. Heemskerk (Leiden University Medical Center, The Netherlands). All cell lines were routinely tested by PCR for the presence of mycoplasma (Minerva Biolabs). Cells were maintained in RPMI-1640 (PAA Laboratories) supplemented with 10% fetal bovin serum (FBS; Gibco) and 50 μg/mL gentamycin (Gibco) (complete RPMI medium) in a humidified atmosphere at 37°C, 5% CO_2_.

#### Human samples

All patients’ samples were collected using a written informed consent. The written informed consent of biobanked material to be used for *in vitro* and *in vivo* research in Oslo and Bergen is based on the legislations and in accordance with the Declaration of Helsinki. The study was conducted after approval from the Regional Committees for Medical and Health Research Ethics (https://www.forskningsetikk.no/en/about-us/our-committees-and-commission/rek/). BM samples were collected from twenty-five consenting AML patients (2015/1012/REK sør-øst D). Two BM with matched-blood samples were obtained from healthy donors at BM donation. Buffy coats were purchased from the hospital. Healthy PBMCs and healthy/AML patient-derived BMMCs were isolated by density gradient and cryopreserved in liquid nitrogen. Peripheral blood samples were collected from 59 informed and consenting AML patients (REK VEST 2015/1759) and healthy peripheral blood samples were collected from 5 healthy consenting donors according (REK vest 2012/2247). The PBMCs from PB samples in this cohort (*n* = 64) were isolated by density gradient centrifugation and cryopreserved before mass cytometric assessment.

#### Mouse xenograft studies

The study design was approved by the Norwegian Food Safety Authority (FOTS ID 29646). NOD-Prkdc^scid^ -IL2rg^Tm1^/Rj (NOD xenograft gamma, NXG) mice were bred in-house and maintained in pathogen-free conditions under an approved institutional animal care protocol. Pilot studies were conducted for each xenograft to evaluate the time-to-engraftment, tumor growth and the onset of clinical signs to human endpoints. Six-to 10-week-old NXG mice were injected intravenously (i.v) with either 5x10^5^ U-937 GFP-Luc^+^, 5x10^3^ MOLM-13 GFP-Luc^+^ or 1x10^6^ primary AML PDX GFP-Luc^+^ cells at 3-, 7- or 7/14-day prior T cell infusion, respectively. Mice were injected intraperitoneally with 200 μL of 20 mg/mL Xenolight D-Luciferin potassium salt to confirm engraftment using *in vivo* imaging system (IVIS spectrum, PerkinElmer). Next, mice were allocated to each treatment group, such that each group had a similar representation of engraftment levels. At the indicated time point, mice were then infused with effector T cells adjusted to the same percentage of CAR-expressing cells with Mock T cells. IVIS analysis was repeated weekly, and the condition of the mice was assessed at least twice weekly.

### Method details

#### Gene expression analysis

The transcriptomic datasets were downloaded from Bloodspot,^66^ the GDC data portal and the European Genome-Phenome Archive (EGA).^70^ From Bloodspot, curated and normalized AML microarrays were acquired from 2 datasets. First set was the MILE study^45^ (*n* = 254; GSE13159) and the second set was a pool of different studies (*n* = 2074; GSE13159, GSE15434, GSE61804, GSE14468 and TCGA). Human healthy hematopoietic cells microarrays were from GSE42519 (*n* = 29). From the GDC data portal, we used RNA-seq data from TCGA (release July 27, 2022), project Acute Myeloid Leukemia (LAML; *n* = 150). To this end, RNA-seq raw data and the corresponding patient clinical data were downloaded using TCGAbiolinks.^70^ Analysis was performed using R (https://www.r-project.org/) using Bioconductor (https://www.bioconductor.org) packages. Harmonized database (https://portal.gdc.cancer.gov/) which is mapped to the reference genome GRCh38 (hg38) was used. Raw count normalization, low-count gene pre-filtering, experiment design and differential expression analysis were performed using DESeq2.^71^ In this analysis, the clinical factor of prognosis was used as a specific coefficient in the design experiment. For FDR correction, adjusted *p* values lower than 0.05 were selected. ‘Pheatmap’ and ‘EnhancedVolcano’ packages were used to visualize the differential expression analyses. For the European Genome-Phenome Archive (EGA), data were obtained from EGAD00001004187, DAC: EGAC00001000956 datasets. This dataset contains 100 sequences cryopreserved bone marrow and peripheral blood samples from patients with AML with 10–90% blasts were selected from the biobank of the Department of Hematology of Leiden University Medical Center (LUMC). For the CD37 transcript in total 88 samples were compared, where of 75 samples were AML bone marrows and 13 normal bone marrows. Counts were quantified from fastq files using Kallisto software (https://pachterlab.github.io/kallisto/about) v0.46.1 and imported into R using tximport package version 1.26.1. The data was normalized with TMM-Normalization in edgeR package, version 3.40.2, and later log2 transformed.

#### Single cell transcriptomic analysis

All preprocessing and analysis steps of scRNA-seq data were run in Python 3 using Scanpy^71^ v.1.4.6 to 1.9.1 and anndata^72^ v.0.7.1 to 0.8.0 unless otherwise stated. All scRNA-seq figures were plotted using matplotlib and seaborn. We obtained raw, annotated count data of healthy bone marrow cells from van Galen et al.^43^*.* from Gene Expression Omnibus (GSE116256). Here, we excluded individual AML916, as it had a mixed AML phenotype expressing markers of stem cells, myeloid, T and B lineages. Barcodes were filtered for each sample for high-quality cells based on the total distributions of unique molecular identifier counts and genes, excluding cells with a fraction of mitochondria-encoded genes over 20%. Barcodes that could not be confidently assigned to either healthy or tumor cells were discarded. Genes detected in less than 20 cells were excluded from further analyses. The resulting count matrix was used for normalization. Unique molecular identifier counts of each cell were normalized using the SCRAN algorithm as implemented in the R-based package.^73^^,^^74^ The top 4,000 variable genes were identified based on normalized dispersion, as described previously,^75^ using Scanpy’s pp.highly_variable_genes. Principal-component analysis dimension reduction was performed by computing 15 principal components on highly variable genes using Scanpy’s pp.pca. Next, a neighborhood graph was computed on the first 50 harmony-adjusted principal components using Scanpy’s pp. neighbors with 15 neighbors. For two-dimensional visualization, embedding the neighborhood graph via UMAP^76^ was done by running Scanpy’s tl.umap with an effective minimum distance between embedded points of 0.5.

#### Mass cytometry

Cryopreserved PBMCs were fixed with 2% PFA and barcoded using Cell-ID 20-Plex Palladium Barcoding Kit according to the manufacturer’s protocol. The samples were distributed in four barcode pools where each barcode contained up to 20 samples. All antibodies used in this study were either purchased pre-conjugated from Standard Bio-Toolsor were conjugated in-house using X8 MaxPar conjugation kits according to the manufacturer’s protocol. Aliquots of 1.5x10^6^ – 3.0 x 10^6^ cells were blocked using an anti-human FcR blocking reagent (130-059-901, Miltenyi Biotec) and stained with antibody panel (Table S2) mastermixes in a staining volume of 100 μL per 3.0 x 10^6^ cells for 30 min at room temperature on shaker. The antibody dilutions used are a result of previous titration experiments and peer recommendation. The cells were permeabilized for 10 min on ice using pure methanol (−20°C, 100%), treated with heparin (100 IU/mL, 20 min) and subsequently stained with intracellular antibodies (30 min, room temperature on shaker). To ensure the identification of cells, DNA was labelled with iridium-191/193 by incubation in 0.1 nM Ir-nucleic acid intercalator (Standard Biotools, San Francisco, CA, USA) diluted in MaxPar PBS containing 4% PFA (Alfa Aesar, 16% PFA, methanol-free) overnight at 4°C. The samples were washed, strained and pelleted prior to acquisition on the XT mass cytometer (Standard Bio-Tools). The Automatic sampling carousel on the XT mass cytometer resuspends the samples in MaxPar Cell Acquisition Solution Plus supplemented with a 1:10 dilution of the EQ Six Element calibration beads (Standard Bio-Tools). The acquisition rate was kept below 300 cells per second to reduce the chances of clogging and doublet acquisition. The MATLAB barcode de-convolution tool was used for de-barcoding samples. Sample preprocessing was performed with Premessa. Data analysis was conducted in Cytobank.org and in the statistical programming tool RStudio. We performed dimensionality reduction using the tSNE-CUDA algorithm and unsupervised self-organizing clustering using FlowSOM in Cytobank.^69^ The FlowSOM included 2 286 000 cells distributed into 196 clusters and 10 meta clusters using 23 phenotypic markers; CD45, CD3, CD34, CD123, CD7, HLA-DR, CD8a, CD19, CD45RA, CD38, CD4, CD64, CD16, CD56, CD33, CD90, CD14, CD45RO, CD44, CD25, CD300e, CD11c and CD11b (Table S2). Marker enrichment modeling (MEM) heatmaps were constructed in RStudio from FlowSOM-identified meta clusters. The MEM R script produces a heatmap of MEM values with a summary of feature enrichment as the population (row) names. The + or −value provided along with the marker name is converted to a −10 to +10 scale and rounded to the nearest integer. The MEM-created population labels aids in identification and correct naming of rare phenotypic clusters in complex sample datasets. The patients’ relative meta cluster abundances were depicted by stacked bar plots using RStudio.^77^

To study T cells in mice, CAR T cells were fixed using Stable-Lyse V2 and Stable-Store V2 (Smart Tube Inc., USA) according to the manufacturer’s protocol, and cryopreserved at −80°C in 10% V/V FBS in DMSO. At endpoint, the spleen and bone marrow cells from femurs were harvested from the euthanized experimental mice. Single-cell suspensions from murine spleen were obtained by mechanical maceration using glass microscopy slides in a Petri dish containing a small amount of cell culture medium. The bone marrow cells were flushed out using cell culture medium delivered by syringe. Suspension materials were filtered through 40 μm cell strainers, fixed and cryopreserved in the same manner as describe above. All antibodies were either sourced in their metal-conjugated form directly from Standard Bio-Tools Inc. or conjugated in-house using X8 MaxPar conjugation kits (Standard Bio-Tools Inc., USA) according to the manufacturer’s protocol.

Samples were slowly thawed at 4°C, washed, and barcoded using Cell-ID 20-Plex Palladium Barcoding Kits (Standard BioTools Inc., USA) into five batches of up to 20 palladium-barcoded samples. Each barcoded sample contained up to 4.0∗10^6^ cells. All samples from the same barcode batch were pooled together to enable uniform and consistent antibody staining. Every batch contained aliquots of two types of anchor samples to be used for the elimination of technical variability due to differences in sample staining conditions by means of batch correction. One type of anchor sample consisted of a mix of CD37-targeting CAR T cells, GFP-positive AML cells, phytohemagglutinin-stimulated healthy human donor PBMCs, and unstimulated healthy donor PBMCs. The composition of the mix would enable the detection of a “positive” signal on all markers present in the panel, which would, in turn, enable batch correction to be performed on each. The second type of anchor consisted solely of healthy human donor PBMCs and was meant to serve as a control for the results of batch correction. The functionality of the anchor samples and of the antibody panel was mutually validated.

Prior to surface antibody staining, human and murine Fc receptors were blocked using human (cat. no. 130-059-901, Miltenyi Biotec, Germany) and murine (cat.no. 15288387, eBioscience, USA) FcR blocking reagents and incubated with a biotinylated goat anti-murine-IgG antibody (cat. no. 115-066-072, Jackson ImmunoResearch Laboratories Inc., UK), which served as a primary antibody for marking the murine part of the chimeric antigen receptor of the anti-CD37 CAR T cells. After several washes, the cells were surface-stained on a shaker for 30 min at room temperature with a mix of the appropriate surface-antigen-targeting antibodies from the panel (Table S4) at a concentration of 3 x 10^6^ cells per 100 μL of staining volume. Following several washes, the cells were permeabilized overnight at −20°C in 100% methanol. The following day, cells were washed, filtered through a 35 μm mesh, and stained with intracellular-antigen-targeting antibodies from the panel (Table S4) under the same conditions as when stained with the surface-antigen-targeting antibodies. Excess antibody was washed away, and the cells’ DNA was labeled with iridium by incubation in Cell-ID Intercalator-Ir (Standard Bio-Tools Inc., USA) diluted to 250 nM in MaxPar Cell Staining Buffer (Standard Bio-Tools Inc., USA) containing 2.9% PFA for 10 min at room temperature. The purpose of this DNA labeling was to aid in the detection of cellular events during data acquisition. Stained cells were washed and cryopreserved at −80°C in 10% V/V FBS in DMSO. On the day of data acquisition, cells were thawed, filtered through a 35 μm mesh, counted on a Countess II automated cell counter (Thermo Fisher Scientific Inc., USA), and distributed into 5 mL polypropylene tubes in such a way that a concentration of 8 x 10^5^ cells per mL would be achieved upon resuspension by the mass cytometer in order to keep the acquisition rate below 300 events per second. This event rate was deemed to present an optimal balance of data acquisition speed and clog avoidance. For acquisition, cells were centrifuged into pellets.

Phenotypic data was acquired on the CyTOF XT Mass Cytometer (Standard Bio-Tools Inc., USA). Cell pellets were resuspended by the mass cytometer using MaxPar Cell Acquisition Solution Plus (Standard Bio-Tools Inc., USA) containing a 1:10 dilution of EQ Six Element Calibration Beads (Standard Bio-Tools Inc., USA). Signal decline over time was corrected internally by the CyTOF XT mass cytometer software using the six-element bead data. Sample data was debarcoded using the MatLab-based debarcoding tool developed by Eli Zunder.^67^ Certain samples occupied multiple barcode slots due to the large number of cells they contained. All parts of their data were concatenated back together into one file using the Premessa R-package. Files containing data on over 52 million events were uploaded to Cytobank.org for cleanup by manual gating of event length, EQ bead and doublet exclusion, and Gaussian gating. Human cells were manually gated out according to expression of human and murine CD45. Cleaned up files containing data from approximately eight million human cells were downloaded from Cytobank.org and subjected to batch correction on the basis of the anchor samples in each barcode batch using a graphical-user-interface-based implementation (cytoBatchNorm - unpublished as of yet) of the CytofBatchAdjust batch correction algorithm.^78^

The diffcyt R package^68^ was mainly used for data analysis. Its internal implementations of the flowCore, CATALYST and FlowSOM^69^ packages were used for the clustering, visualization and quality control of the data. CAR T cells were separated out of the human cell parent population by clustering on the expression of the hCD45, CD3, CD123 and CD45RA markers into 64 clusters, and manual metaclustering into AML and CAR T cellT-cell metaclusters. In the same manner CAR T cellT-cell subsets were inferred from the results of clustering of the CAR T cellT-cell data into 100 clusters on the expression of the CD4, CD8, CD45RA, CD45RO, CD27, CD95, CD184, CD185, CD196 and CD197 markers, and manual metaclustering. Each step of clustering and metaclustering was thoroughly checked using heatmaps, UMAPs, biaxial plots. The metacluster assignment of each individual cluster was verified on Cytobank.org. To get deeper insight into the data of the CAR T cells, the CAR T cells were metaclustered into several nested sets of metaclusters of different granularities. The exploration of the data to find significant differences in abundances or cell state marker signal expression in CAR T cellT-cell metaclusters between different sets of samples was done on Cytobank.org using the website’s built-in data plotting and exploration tools. All *p*p-values were inferred by paired or unpaired student’s t-tests and one-way ANOVAs. *p*P-values were corrected for multiple hypothesis testing using the Benjamini-Hochberg method and Tukey’s range test.

#### DNA constructs

The HH1 antibody-based CD37CAR and the fmc63-based CD19CAR designs were previously described.^32^ The humanized mouse anti-human CD33 gemtuzumab ozogamicin sequence from https://go.drugbank.com/drugs/DB00056 and was designed as the other scFv: [leader sequence-light chain-(G_4_S)_4_-heavy chain] fused to the CD8 hinge and transmembrane domains (amino acid 128–210, UniProt P01732) followed by the 4-1BB intracellular costimulation domain (amino acid 208–255, UniProt P07011) and the CD3ζ signaling unit (amino acid 52–164, UniProt P20963) (see Figure S6). CD33CAR was designed as above followed by a T2A ribosome skipping sequence and a truncated sequence of CD34 (CD34t; UniProt P28906, Figure S4) downstream of the CD3ζ signaling unit. Codon optimized sequences were purchased (Eurofins, Erlangen Germany), cloned into the Gateway system pENTR (Thermo Fisher Scientific) and further subcloned into the retroviral vector pMP71. The GFP-firefly luciferase (GFP-Luc) fusion protein coding sequence (a kind gift from Rainer Löw)^79^ was incorporated into pMP71 and used to stably transduce target cell lines as reported in.^32^ The full-length CD37 isoform-1 (UniProt P11049-1), isoform-2 (UniProt P11049-2) and isoform-3 (UniProt P11049-3) were ordered as codon optimized DNA (Eurofins) and cloned into pMP71.

#### Immunophenotyping by flow cytometry

The following anti-human antibodies were purchased from BD Biosciences unless specified: CD3-BV421 and BV605 (SK7), CD4-BV421 and BV605 (RPA-T4), CD8-BV605 and PE-Cy7 (RPA-T8 from BioLegend and Thermo Fisher Scientific), CD11b-PE (ICRF44), CD14-APC-Cy7 (MφP9), CD19-BV421 (HIB19) and PE (HIB19 from Thermo Fisher Scientific), CD33-BV421 (WM53), CD34-PE-Cy7 and APC (581 and 4H11 from Thermo Fisher Scientific), CD37-Af647 (M-B371 and HH1 from Santa Cruz Biotechnologiy), CD38-APC-R7 (HIT2), CD45-FITC (HI30), CD45RA-BV510 and BB515 (HI100), CD56-APC (NCAM16.2 from Thermo Fisher Scientific), CD69-PE-Cy5 (FN50), CD107a-PE-Cy5 and APC (H4A3), CCR7-FITC (150503), CD90-BV605 (5E10), CD123-PE and PerCP-Cy5.5 (7G3), and Lineage cocktail 2 (CD3, CD14, CD19, CD20, CD56)-FITC (SK7, MφP9, Sj25G1, L27 and NCAM16.2). CD19^−^and CD37CAR expression were detected by biotinylated goat anti-mouse Fab antibody (Jackson ImmunoResearch) followed by streptavidin-PE (BD Biosciences). CD33CAR expression was detected using CD34-APC (4H11). Flow cytometry was performed on BD FACSCanto or LSR II instruments (BD Biosciences) and analyzed with the FlowJo software (TreeStar). To study the glycosylation of CD37, AML cell lines have been incubated 30 min, at 37C, with 0.1 U/mL of Neuraminidase in PBS neuraminidase before the staining. For the staining of AML cells, PBMCs and BMMCs, Fcγ receptor blocking was performed using BD Fc Block (BD Biosciences) prior to any relevant staining. Isotype controls were used to set the threshold of background fluorescence for each cell population of interest. Propidium Iodide (PI) was used to discriminate dead cells (Thermo Fisher Scientific). CD37 antigen density was evaluated using the QIFIKIT following the manufacturer’s instructions (Dako). A special order of unconjugated anti-CD37 (HH1, Santa-Cruz) antibody and the corresponding isotype were used for the detection in that assay.

#### Retroviral transduction and expansion of human T cells

Human PBMCs were isolated from healthy donors’ buffy coat as described above. Retroviral supernatants were produced as previously described.^32^ T cells from PBMCs were first activated before transduction. Briefly, PBMCs were resuspended in X-VIVO 15 medium (Lonza) supplemented with 5% human serum (PAN Biotech) and 100 U/mL recombinant human IL-2 (Clinigen) (complete T cell medium) at 0.5x10^6^ cells/mL. Two milliliters of cells were then transferred per well of 24-well plate, previously coated with 1 μg/mL anti-CD3 and anti-CD28 (OKT3 and CD28.6, Thermo Fisher Scientific) and incubated at 37°C for 48 h. Activated T cells were transferred to a non-tissue culture-treated 24-well plate previously coated with 50 μg/mL retronectin (Takara Bio. Inc.). Retroviral supernatant was added, and the cells were spun down at 750g, 32°C for 1 h. T cells were transduced a second time the next day following the same procedure. On day 3, medium was renewed with complete T cell medium. On day 4, the transduction efficiency was evaluated by flow cytometry and cultures were scaled-up. Thereafter, cells were refreshed every 2 days with complete T cell medium by dilution to 1x10^6^ cells/mL for 7 days. Some experiments were performed with T cells maintained in media supplemented with 30 nM of dasatinib from day 2 to day 11 before being washed and switched back to media without dasatinib. On specific days of the expansion, viability and cell concentration was evaluated using a Countess II Automated Cell Counter (Thermo Fisher Scientific). Expanded T cells were frozen in batches and transferred to liquid nitrogen.

#### Isoform detection assay

HEK-P cells were seeded at 1.2x10^6^ cells/well of a 6-well plate and co-transfected 24 h later with a vector encoding a single CD37 isoform and a vector encoding GFP. The cells were stained 48 h later using 2 different clones of commercial anti-CD37 antibody (HH1 and M-B371). An aliquot of cells was lysed in RIPA buffer to confirm protein expression in Western blot. Lysates were run in SDS-PAGE with β-mercaptoethanol and transferred onto PVDF membranes (Bio-Rad). Western-blot of CD37 isoforms were detected using an anti-HA tag antibody (Invitrogen, mAb clone 2–2.2.14, 1:5000) or an anti-CD37 antibody (Cell Signaling Technology, mAb clone E4K2M, 1:1000) overnight at 4°C. The primary antibody was detected using a goat anti-rabbit IgG antibody conjugated to horseradish peroxidase (Invitrogen, 31460, 1:2000).

#### Reporter assay

Jurkat76 cells were first stably transduced with pSIRV-NFAT-eGFP which was a gift from Peter Steinberger (Addgene plasmid # 118031; http://n2t.net/addgene:118031; RRID:Addgene_118031),^45^ and a single cell clone was selected by limiting dilution (J76^NFAT−GFP^). The cells were next transduced with different CAR constructs as described above (CAR-J76^NFAT−GFP^). Target cells were labelled with Cell Trace Violet (Thermo Fisher Scientific) according to the manufacturer’s recommendations to distinguish them from effector cells. CAR-J76^NFAT−GFP^ were cocultured with target cells for 24 h at an effector-to-target (E:T) ratio of 1:2 in complete RPMI medium. The percentage of CAR-expressing population was adjusted with untransduced J76^NFAT−GFP^ cells for each CAR-J76^NFAT−GFP^. After incubation, the GFP signal was measured by flow cytometry.

#### Generation of CD37 knock-out U-937cells

U-937 cells were first retrovirally transduced with MSCV_Cas9_puro which was a gift from Christopher Vakoc (Addgene plasmid # 65655; http://n2t.net/addgene:65655; RRID:Addgene_65655)^80^ and selected for 48 h with 0.1 μg/mL puromycin (Thermo Fisher Scientific). The cells were then electroporated with a sgRNA targeting at least the 3 main CD37 splice variants (CRISPR1015113_SGM, Invitrogen) using a BTX 830 Square Wave Electroporation System (BTX Technologies). The electroporation was performed with 3x10^6^ U-937-Cas9^+^ cells and 7.5ug of CD37 sgRNA, in a 1-mm gap cuvette, at 125 V for 2 ms. Immediately after electroporation, cells were transferred to complete RPMI medium and further expanded for 3 days. CD37 knock-out cells (U-937^*CD37KO*^) were finally stained with anti-CD37 (HH1) and sorted on the negative population.

#### Degranulation analysis

Effector T cells and target cells were resuspended in complete RPMI medium and plated at an E:T ratio of 1:2 in a 96-well plate in duplicates. Then, anti-CD107a (H4A3) antibody, GolgiStop and GolgiPlug (all from BD Biosciences) were added, and the plate was incubated at 37°C, 5% CO_2_ for 6 h. After incubation, T cells were stained with anti-CD3 (SK7) antibody (BD Biosciences), and flow cytometry was performed.

#### Cytokine quantification

Effector T cells and target cells were resuspended in complete RPMI medium and plated at an E:T ratio of 1:2 in a 96-well plate in duplicates. For AML primary cells, cells were seeded in plain X-VIVO 15 medium w/o phenol red (Lonza) instead. After 24 h, the cells were spun down, and the supernatant transferred into another plate and kept at −80°C. Upon thawing, supernatants were diluted 1:3 in plain X-VIVO 15 w/o phenol red supplemented with 2.5% human serum albumin (Octa pharma). Cytokines were quantified using the Bio-Plex Pro Human Cytokine 17-plex assay on a Luminex 200 system (Bio-rad).

#### Bioluminescence (BLI)-based cytotoxicity assay

The killing assay was performed as reported.^32^ Briefly, luciferase-expressing target cells (GFP-Luc^+^) were mixed with 75 μg/mL Xenolight D-Luciferin potassium salt (PerkinElmer) and seeded in 96-well white plates in triplicates. For some assay, target cells have been treated in with 0.1 U/mL of Neuraminidase in PBS, for 30 min at 37°C. Then the cells were washed twice before adding D-Luciferin potassium salt. Effector T cells were added at indicated E:T ratios and incubated at 37°C, 5% CO_2_. BLI was measured with a luminometer (PerkinElmer) as relative light units (RLU). Triplicate wells were averaged, and lysis percentage was calculated using the following equation: % specific killing = 100 x (spontaneous cell death RLU – sample RLU)/(spontaneous death RLU – maximal killing RLU).

#### Cytometry-based cytotoxicity assay

Healthy PBMCs and AML BMMCs used as target cells were first labelled with Cell Trace Violet (Thermo Fisher Scientific) following the manufacturer’s instructions. Effector T cells and target cells were resuspended in plain X-VIVO 15 medium and plated at an E:T ratio of 2:1 (5:1 against AML BMMCs) in a 96-well plate, in duplicates. After 24 h of incubation, cells were collected, and the plate was treated with Accutase (Thermo Fisher Scientific) for 3 min at 37°C to release adherent cells. PBMCs were subsequently stained for lineage markers while anti-CD3, -CD4, -CD34, -CD38 and -CD45 antibodies were used for AML BMMCs. Counting beads (Thermo Fisher Scientific) were added to quantify the absolute count of cells. PI was added extemporary before flow acquisition to gate out dead cells. For monocyte cytoxicity assays, monocytes were magnetically isolated from healthy PBMCs using the Pan Monocyte Isolation Kit human (Miltenyi Biotec) according to the manufacturer’s instructions. The sorted cells were then stained for lineage markers with anti-CD3, -CD11b, -CD14, -CD16, and CD20 antibodies to confirm the population. Then, monocytes were labeled with CellTrace CFSE (Thermo Fisher Scientific) following the manufacturer’s instructions, before being co-cultured with Mock and CD19^−^, CD37 T cells at 37C, for 12 to 15h. Effector T cells and target monocyte, from the same healthy donor, were resuspended in regular X-VIVO 15 medium and plated at a 2:1 E:T ratio in ultra-low attachment 96-well plates. After incubation, cells were harvested and labeled with anti-CD33, -CD3, -CD11b, -CD14, -CD20 antibodies. Counting beads were added to quantify the absolute count of cells. PI was added extemporary before flow acquisition to gate out dead cells.

#### Colony-forming unit (CFU) assay

BMMCs were thawed the day of the assay and an aliquot of cells was left untreated for 6 h at 37°C, 5% CO_2._ Effector T cells were co-cultured with autologous BMMCs for 6 h in complete RPMI medium at an E:T ratio of 10:1. After incubation, cells were washed and adjusted to 2.5x10^5^ alive BM cells/mL in a final volume of 300 μL. For this purpose, the aliquot of untreated BMMCs was used to estimate the true viability count upon thawing (no effectors). The solution was then homogenized with 4 mL of complete MethoCult medium (STEMCELL), and 1.1 mL was transferred per well of 6-w plate (in triplicate). After 10 days, Colony-forming unit-erythroid (CFU-E), Burst-forming unit-erythroid (BFU-E) and colony-forming unit-granulocyte, macrophage (CFU-GM) colonies were counted under a microscope using a background grid.

### Quantification and statistical analysis

All experimental data are represented as individual values with the mean or the mean ± SD. Comparisons between two groups were assessed by two-tailed paired Student’s t-test. Statistical differences among three or more groups were evaluated with One-way ANOVA corrected with post-hoc tests. two-way ANOVA corrected with post-hoc tests was used to assess differences between two variables among three or more groups. Survival studies were assessed by Kaplan–Meier curves and the log rank (Mantel-Cox) test. Data were analyzed with Prism 9 software (GraphPad Software). *p* values lower than 0.05 were considered statistically significant and written as follow: ∗ = *p* < 0.05, ∗∗ = *p* < 0.01, ∗∗∗ = *p* < 0.001, ∗∗∗∗ = *p* < 0.0001.

## Supplemental information

Document S1. Figures S1–S13 and Tables S1–S4

Document S2. Article plus supplemental information
