# Supplementary material for: CD37 is a safe chimeric antigen receptor target to treat acute myeloid leukemia
Source: Cell Rep Med. 2024 May 15;5(6):101572. doi: 10.1016/j.xcrm.2024.101572 (PMC11228397; doi:10.1016/j.xcrm.2024.101572)
Supplement: Document S1. Figures S1–S13 and Tables S1–S4 [file mmc1.pdf]

Cell Reports Medicine, Volume *5*

Supplemental information

CD37 is a safe chimeric antigen receptor target to treat acute myeloid leukemia

Benjamin Caulier, Sandy Joaquina, Pascal Gelebart, Tara Helén Dowling, Fatemeh Kaveh, Moritz Thomas, Luka Tandaric, Patrik Wernhoff, Niveditha Umesh Katyayini, Cara Wogsland, May Eriksen Gjerstad, Yngvar Fløisand, Gunnar Kvalheim, Carsten Marr, Sebastian Kobold, Jorrit M. Enserink, Bjørn Tore Gjertsen, Emmet McCormack, Else Marit Inderberg, and Sébastien Wälchli

Figure S1. Staining with anti-CD37 antibodies

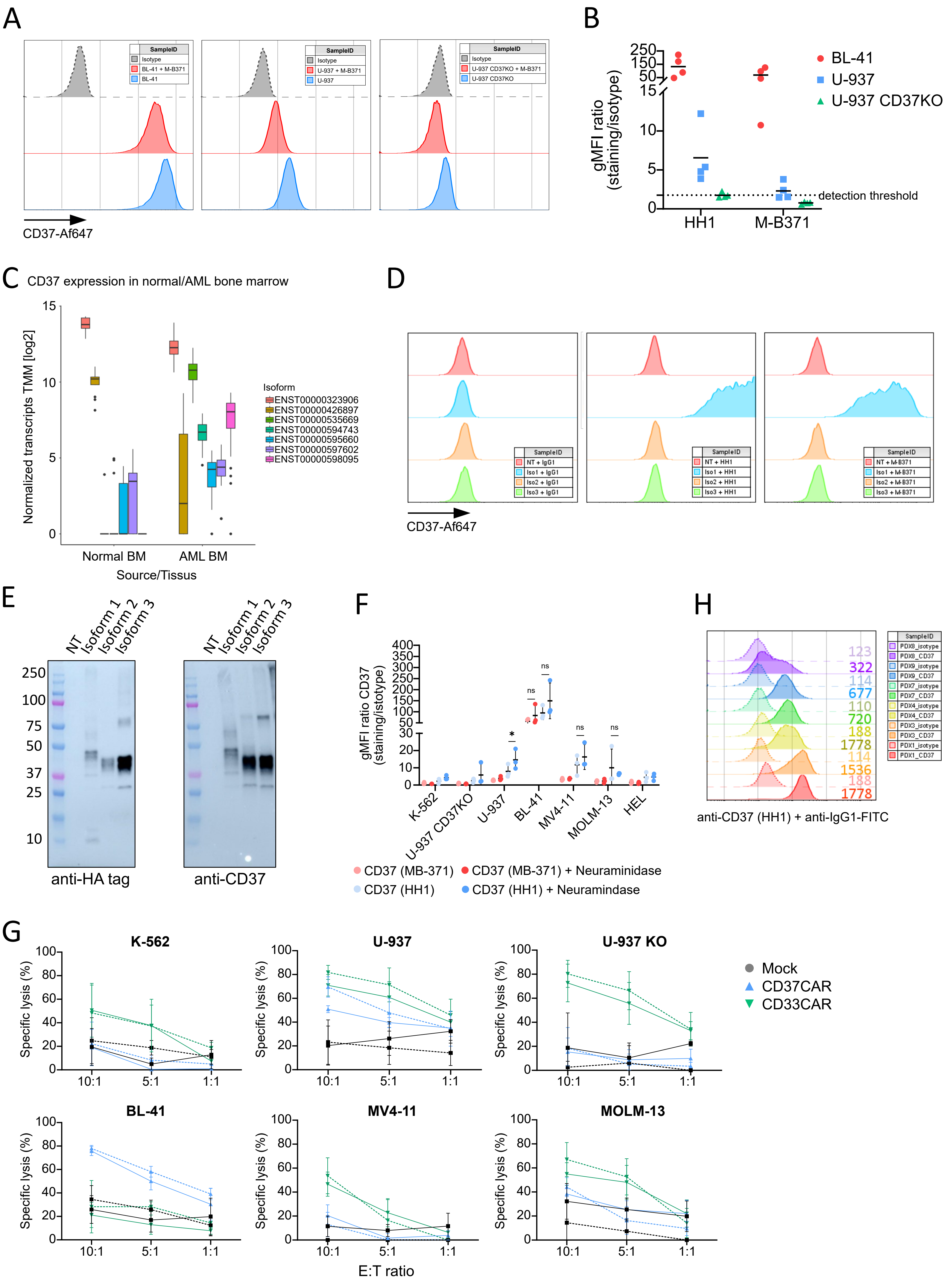

Figure S1. Staining with anti-CD37 antibodies. Related to Figure 1. (A) Representative detection of CD37 on B cell lymphoma (BL-41) and AML cells (U-937 WT and KO for CD37) using 2 different clones of commercial anti-CD37 antibody (M-B371 [red histograms] and HH1 [blue histograms], both murine monoclonal IgG1 used at 1 µg per 1x106 cells). A murine IgG1 isotype was used to set the negative threshold and the percentage of positive cells is displayed. (B) Relative quantification of CD37 staining in (A). The geometric Mean Fluorescence Intensity (gMFI) of CD37 staining was divided by the background staining of an isotype control for each cell line (n = 4 experiments). Bars represente the mean. (C) Comparison of CD37 isoform expression between normal bone marrows vs AML bone marrows from the European Genome-Phenome Archive (EGA)68. Normalized AML datasets are from EGAD00001004187, DAC: EGAC00001000956, 75 samples of AML bone marrows and 13 normal bone marrows were analysis. Differences in transcript expression were tested with Kruskal-Wallis test in R and found significant difference for all transcripts. ENST00000323906 p-value = 0.0002991, ENST00000426897 p-value = 1.484e-08, ENST00000598095 p-value = 1.446e-08, ENST00000535669 p-value = 9.464e-09, ENST00000594743 p-value = 9.608e-09, ENST00000595660 p-value = 1.797e-06, ENST00000597602 p-value = 2.794e-05. (D) We tested if the discrepancy in CD37 detection on AML cells between the M-B371 and HH1 antibody clones was due to the specific recognition of one of the three main CD37 isoforms. We observed that both antibodies solely reacted against the isoform-1 of CD37. Representative detection of CD37 isoforms (isoforms 1, 2 and 3) transiently expressed in HEK cells. Cells were co-transfected with a vector encoding a single CD37 isoform and a vector encoding GFP. GFP-positive, Propidium Iodide (PI)-negative HEK cells were stained 48 hours post-transfection for CD37 using 2 different clones of commercial anti-CD37 antibody (HH1 and M-B371). Inserts represent other channel overlays. NT = non-transfected. (n = 1 representative experiment over 2). (E) Western-blot analysis of lysates from (D). CD37 isoforms were detected using an anti-HA tag antibody or an anti-CD37 antibody. The primary antibody was detected using a goat anti-rabbit IgG antibody conjugated to horseradish peroxydase. NT = non-transfected. (F) Relative quantification of CD37 staining on different AML cell lines (U-937, U-937CD37KO, MV4-11, MOLM-13, HEL) treated or not with Neuraminidase. BL-41 (CD37high) and K-562 (CD37null) cell lines have been used as control. The geometric Mean Fluorescence Intensity (gMFI) of CD37 staining was divided by the background staining of an isotype control for each cell line (n = 3 experiments, mean ± SD). AML cell lines treated with neuraminidase showed a trend towards increased detection of the HH1 epitope at the membrane, not a decrease. HH1 does not appear to depend on sialylation. Two-way ANOVA followed by Tukey's multiple comparisons tests. (G) Specific cytotoxicity of T cells incubated 8 hours with target cells (K-562, BL-41, U-937, U-937 CD37KO, MV4-11 and MOLM-13) treated or not with Neuraminidase. E:T ratios indicated. (n = 3 donors, mean ± SD). No significant difference was observed in the killing of AML cell lines either treated or not with neuramindase. Two-way ANOVA followed by Tukey's multiple comparisons tests. No significant difference was observed. (H) Flow cytometry-based, surface CD37 protein quantification of AML-PDX cells (n = 6).
The murine IgG1 anti-CD37 mAb clone HH1 was used for the detection. A murine IgG1 isotype was used to set the background. The numbers indicate the Mean Fluorescence Intensity (MFI).

Figure S2. Detection of CD37 in a small cohort of patients

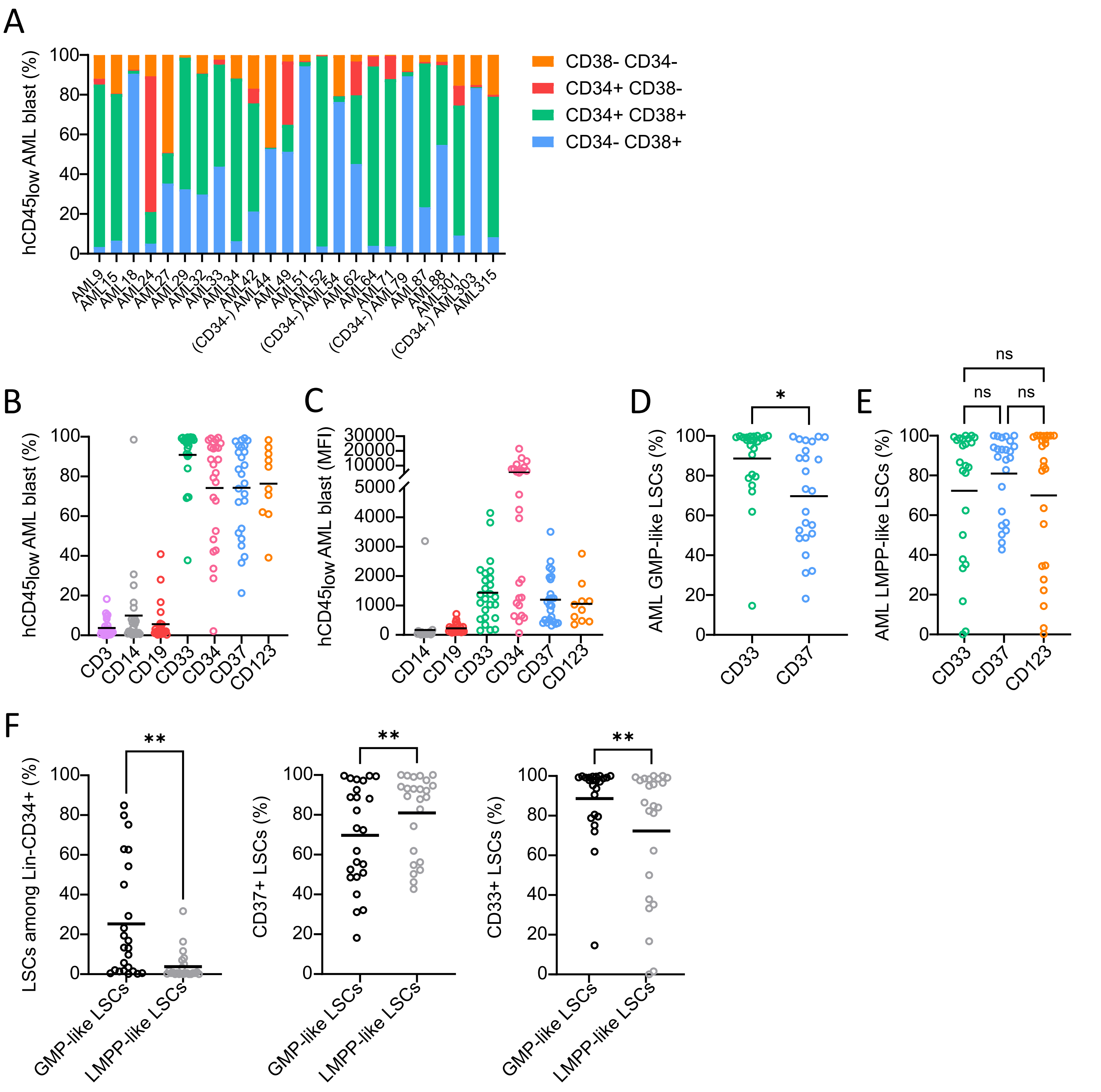

Figure S2. Detection of CD37 in a small cohort of patients. Related to Figure 1. (A) Percentage of CD34/CD38 double-positive cells among the hCD45low blast population of AML BM samples (n=25). Percentage (B) and geometric median fluorescent intensity (C) of CD3, CD14, CD19, CD33, CD34, CD37 (mAb clone HH1) and CD123 surface proteins among the hCD45low blast population of primary AML BM samples (n = 25, mean). A murine IgG isotype was used to set the background for each antibody. (C) Percentage of CD14, CD19, CD33, CD37 and CD123 surface proteins on hCD45low CD34+ AML blast population from primary BM samples (n = 25, mean). One-way ANOVA followed by Dunnett's multiple comparison tests. (D) Percentage of CD33 and CD37 on the granulocyte-macrophage progenitors (GMP)-like LSC population of primary AML BM samples (n = 24, mean). The anti-CD37 mAb clone HH1 was used for detection. A murine IgG1 isotype was used to set the background. GMP-like LSCs were defined as Lin- CD34+ CD38+ CD123+ CD45RA+. Paired t-test (two-tailed) was used for statistical analysis.
(E) Percentage of CD33, CD37 and CD123 among the lymphoid-primed multipotential progenitors (LMPP)-like LSC population of primary AML BM samples (n = 24, mean). The anti-CD37 mAb clone HH1 was used for detection. A murine IgG1 isotype was used to set the background. LMPP-like LSCs were defined as Lin-CD34+ CD38- CD90- CD45RA+. One-way ANOVA followed by Tukey's multiple comparison tests.
(F) (Left) Percentage of granulocyte-macrophage progenitors (GMP)- and lymphoid-primed multipotential progenitors (LMPP)-like leukemic stem cells (LSCs) in primary AML BM samples (n = 25). GMP-like LSCs were defined as Lin- CD34+ CD38+ CD123+ CD45RA+ and LMPP-like LSCs as Lin- CD34+ CD38- CD90- CD45RA+. t-test (paired) was used for statistical analysis. Percentage of CD37 (center) and CD33 (right) on GMP- and LMPP-like LSCs in primary AML BM samples (n = 25). t-test (paired) was used for statistical analysis. Bars represent the mean.

Figure S3. Clustering of AML samples in a large cohort

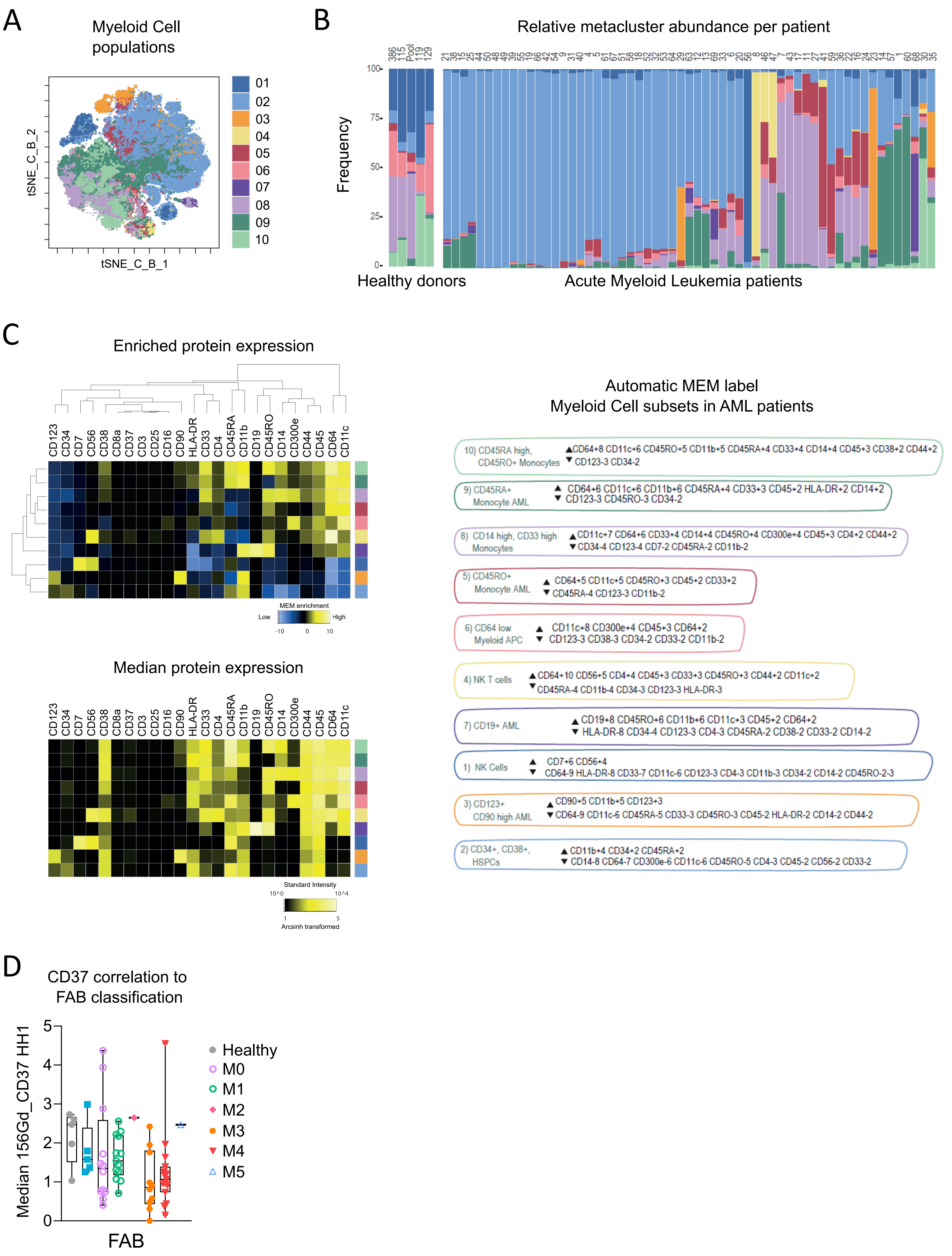

Figure S3. Clustering of AML samples in a large cohort. Related to Figure 1. (A) A tSNE-Cuda of myeloid cells from a concatenated AML sample of 2 286 000 cells (n=59 AML patients) was coloured by metaclusters highlighting position and size of meta cluster. (B) Relative meta cluster abundance as percent of total population in healthy donors to the left (n= 5) and AML patients (n= 59) ordered according to similar meta cluster distribution. (C) Marker expression modelling (MEM) characterizing cell subsets of FlowSOM unsupervised clusters. The MEM labels were computationally assigned using the 9 other metaclusters as reference points. The heatmaps depict the protein enrichment values used to generate the MEM labels and the median protein expression was given for each myeloid cell subset. (D) The distribution of patients in the French American British morphological stratification system did not correlate to CD37 expression. We did not find a significant increase of CD37 expression in FAB M5 as reported in other studies.

Figure S4. Metacluster analysis

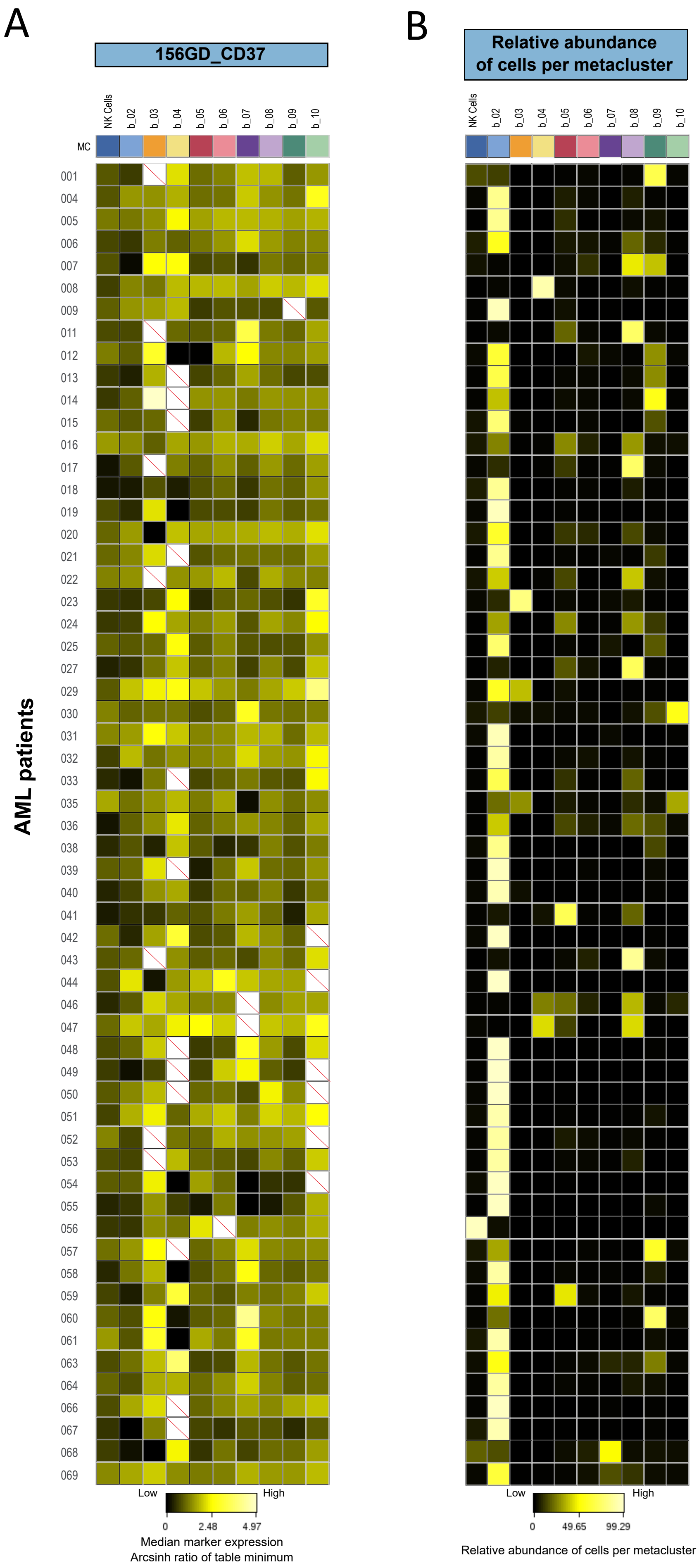

Figure S4. Metacluster analysis. Related to Figure 1. (A) Heatmap of CD37 expression according to meta cluster in each patient shown as median marker expression of arcsinh ratio of tables minimum (n=59). The highest expression levels in patients were observed in MC 3, 4, 7 and 10. (B) Heatmap showing relative meta cluster abundance as percent of total population (n=59). Most patients have the highest frequency of their cells in meta cluster 2.

Figure S5. CD37 mRNA analysis

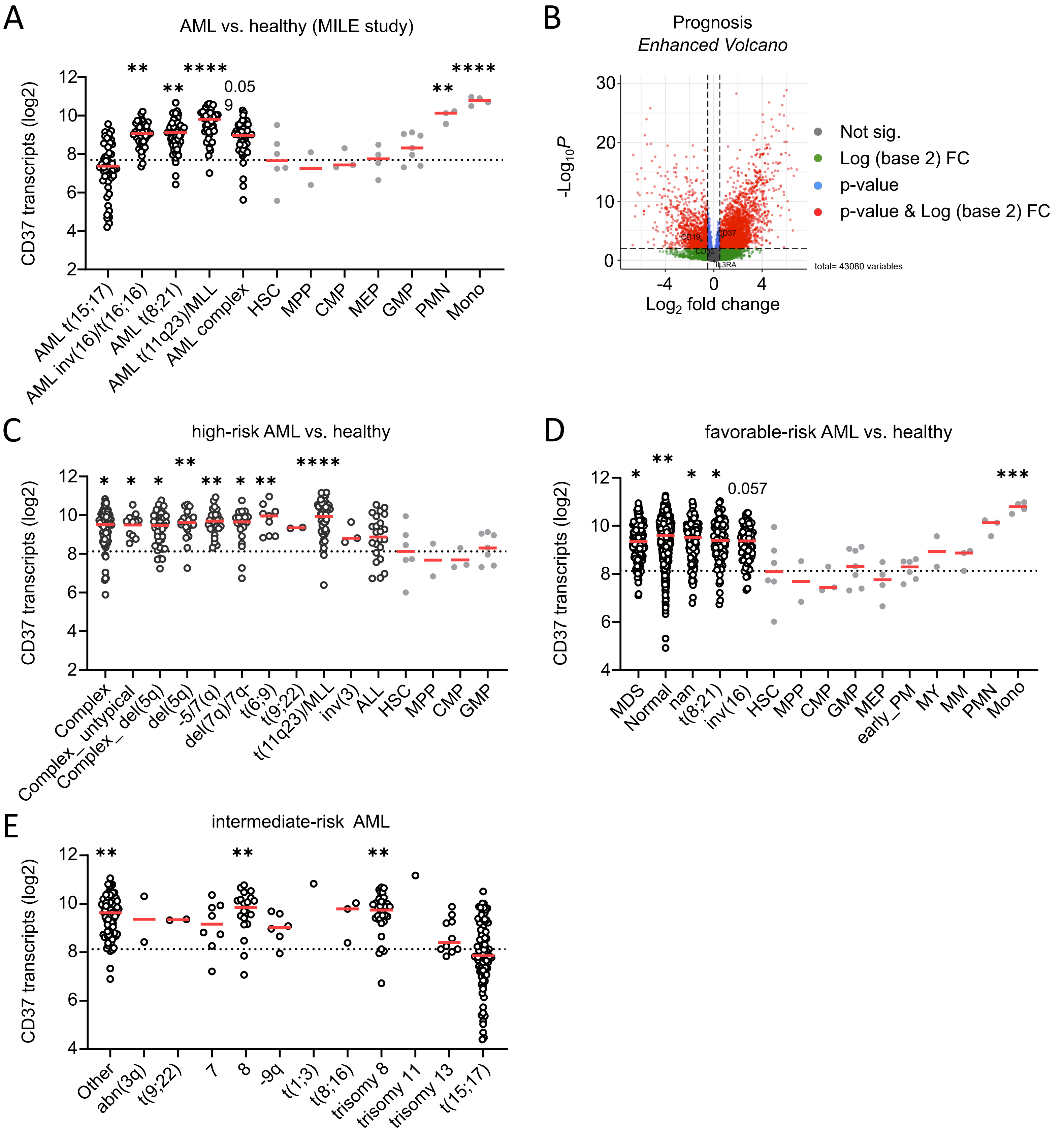

Figure S5. CD37 mRNA analysis. Related to Figure 1. (A) CD37 gene expression analysis of high-risk AML downloaded from BloodSpot67 (n = 242). Curated and normalized AML microarray datasets (black empty circles) are from GSE13159, GSE15434, GSE61804, GSE14468, TCGA-LAML and human healthy hematopoietic cells (filled grey circles) are from GSE42519 (n = 18). The dotted line sets the averaged CD37 expression on HSC. ALL = acute lymphoid leukaemia, HSC = hematopoietic stem cells, MPP = multipotential progenitors, CMP = common myeloid progenitors and GMP = granulocyte-macrophage progenitors. AML abbreviations are defined in Supplementary Table 3. One-way ANOVA tests followed by Dunnett's multiple comparisons versus healthy HSCs. Non-significant statistical tests are not displayed.
(B) Volcano-plot showing the expression of significantly changed mRNAs with FDR < 0.05 and log2FC (fold change) ≥ 0.5. The red-marked dots represent up-regulated genes, the green ones represent down-regulated genes, and the grey ones show no significance. CD37 gene expression analysis of (C) (high-risk), (D) (favourable-risk) and (E) (intermediate-risk) AML downloaded from BloodSpot67 (n = 2074). Curated and normalized AML microarray datasets (black empty circles) are from GSE13159, GSE15434, GSE61804, GSE14468, TCGA-LAML and human healthy hematopoietic cells (filled grey circles) are from GSE42519. The dotted line sets the averaged CD37 expression on HSC. AML and healthy cell abbreviations are defined in Supplementary Table 3. One-way ANOVA tests followed by Dunnett's multiple comparisons versus healthy HSCs. Non-significant statistical tests are not displayed.

Figure S6. Single cell transcriptomic data

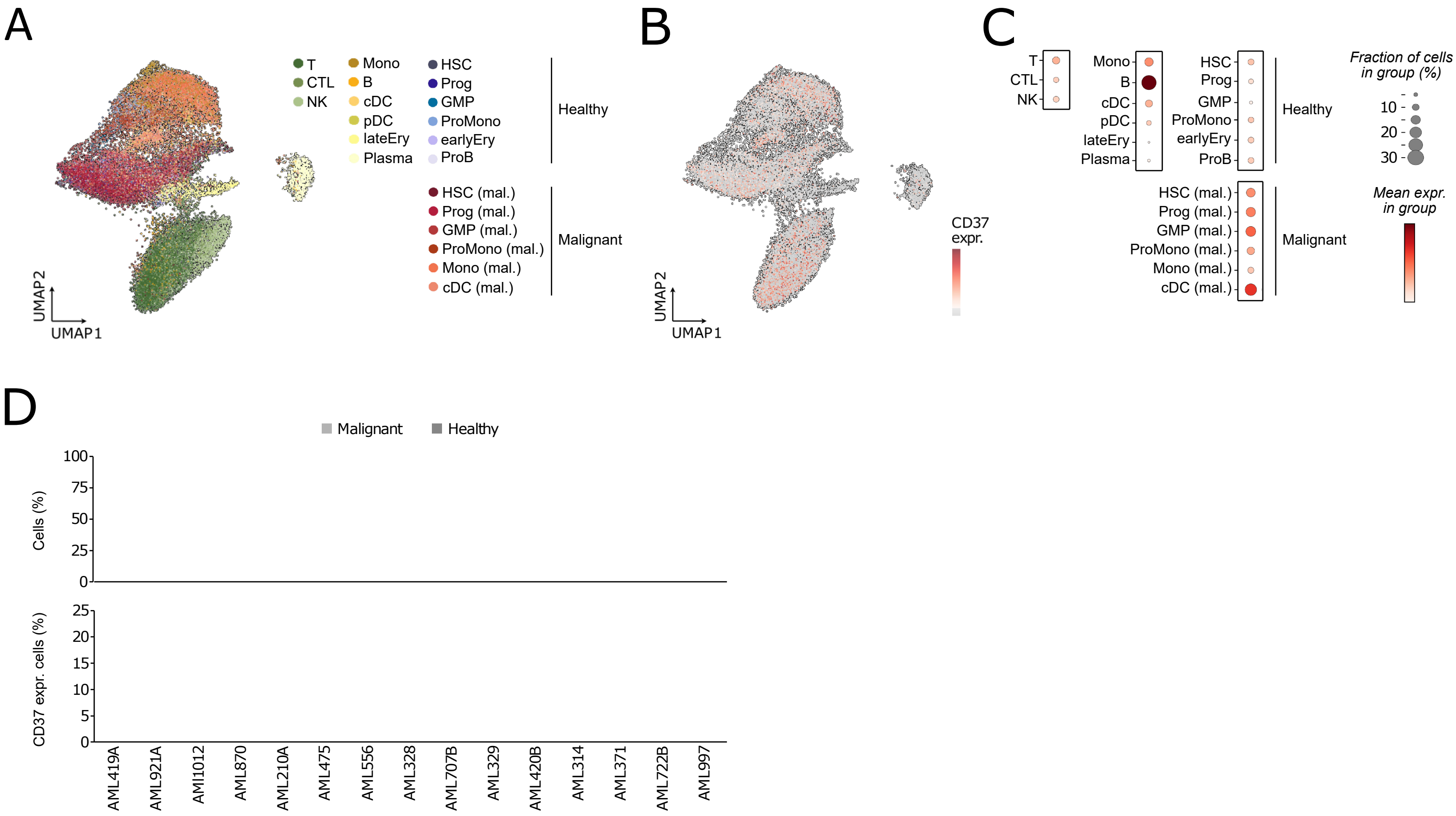

Figure S6. Single cell transcriptomic data. Related to Figure 1. (A) UMAP showing 28,404 healthy and malignant cells from 15 individuals suffering from AML with different mutations1. Colours highlight the 15 different healthy and 6 different malignant cell types. (B) Expression of CD37 in healthy and malignant cells. Normalized gene expression values were log transformed and visualized in a UMAP embedding. (C) Dotplot of CD37 expression on healthy and malignant cell types. Dot size indicates the fraction of cells expressing CD37; colour intensity shows mean normalized gene expression per cell type. (D) Top: amount of malignant and normal cells per AML patient. Bottom: Percentage of malignant and normal cells expressing CD37 for each AML patient.

Figure S7. Constructs

CD19CAR

METDTLLLWVLLLWVPGSTGDIQMTQTTSSLSASLGDRVTISCRASQDISKYLNWYQQKPDGTVKLLIYHTSRLHSGV
PSRFSGSGSGTDYSLTISNLEQEDIATYFCQQGNTLPYTFGGGTKLEITKAGGGGSGGGGSGGGGSGGGGSEVKLQES
GPGLVAPSQSLSVTCTVSGVSLPDYGVSWIRQPPRKGLEWLGVIWGSETTYYNSALKSRLTIIKDNSKSQVFLKMNSL
QTDDTAIYYCAKHYYYGGSYAMDYWGQGTSVTVSSDFVPVFLPAKPTTTPAPRPPTPAPTIASQPLSLRPEACRPAAG
GAVHTRGLDFACDIYIWAPLAGTCGVLLLSLVITLYCNHRNRFSVVKRGRKKLLYIFKQPFMRPVQTTQEEDGCSCRF
PEEEEGGCELRVKFSRSADAPAYQQGQNQLYNELNLGRREEYDVLDKRRGRDPEMGGKPRRKNPQEGLYNELQKDKMA
EAYSEIGMKGERRRGKGHDGLYQGLSTATKDTYDALHMQALPPR

CD37CAR

METDTLLLWVLLLWVPGSTGDIVMTQSHKLLSTSVGDRVSITCKASQDVSTAVDWYQQKPGQSPKLLINWASTRHTGV
PDRFTGSGSGTDYTLTISSMQAEDLALYYCRQHYSTPFTFGSGTKLEIKGGGGSGGGGSGGGGSGGGGSEIQLQQSGP
ELVKPGASVKVSCKASGYSFTDYNMYWVKQSHGKSLEWIGYIDPYNGDTTYNQKFKGKATLTVDKSSSTAFIHLNSLT
SEDSAVYYCARSPYGHYAMDYWGQGTSVTVSSDFVPVFLPAKPTTTPAPRPPTPAPTIASQPLSLRPEACRPAAGGAV
HTRGLDFACDIYIWAPLAGTCGVLLLSLVITLYCNHRNRFSVVKRGRKKLLYIFKQPFMRPVQTTQEEDGCSCRFPEE
EEGGCELRVKFSRSADAPAYQQGQNQLYNELNLGRREEYDVLDKRRGRDPEMGGKPRRKNPQEGLYNELQKDKMAEAY
SEIGMKGERRRGKGHDGLYQGLSTATKDTYDALHMQALPPR

CD33CAR

METDTLLLWVLLLWVPGSTGDIQLTQSPSTLSASVGDRVTITCRASESLDNYGIRFLTWFQQKPGKAPKLLMYAASNQ
GSGVPSRFSGSGSGTEFTLTISSLQPDDFATYYCQQTKEVPWSFGQGTKVEVKRTVAGGGGSGGGGSGGGGSGGGGSE
VQLVQSGAEVKKPGSSVKVSCKASGYTITDSNIHWVRQAPGQSLEWIGYIYPYNGGTDYNQKFKNRATLTVDNPTNTA
YMELSSLRSEDTAFYYCVNGNPWLAYWGQGTLVTVSSFVPVFLPAKPTTTPAPRPPTPAPTIASQPLSLRPEACRPAA
GGAVHTRGLDFACDIYIWAPLAGTCGVLLLSLVITLYCNHRNRFSVVKRGRKKLLYIFKQPFMRPVQTTQEEDGCSCR
FPEEEEGGCELRVKFSRSADAPAYQQGQNQLYNELNLGRREEYDVLDKRRGRDPEMGGKPRRKNPQEGLYNELQKDKM
AEAYSEIGMKGERRRGKGHDGLYQGLSTATKDTYDALHMQALPPREFGSGEGRGSLLTCGDVEENPGPPRGWTALCLL
SLLPSGFMSLDNNGTATPELPTQGTFSNVSTNVSYQETTTPSTLGSTSLHPVSQHGNEATTNITETTVKFTSTSVITS
VYGNTNSSVQSQTSVISTVFTTPANVSTPETTLKPSLSPGNVSDLSTTSTSLATSPTKPYTSSSPILSDIKAEIKCSG
IREVKLTQGICLEQNKTSSCAEFKKDRGEGLARVLCGEEQADADAGAQVCSLLLAQSEVRPQCLLLVLANRTEISSKL
QLMKKHQSDLKKLGILDFTEQDVASHQSYSQKTLIALVTSGALLAVLGITGYFLMNRRSWSPTGERLELEP

Single chain variable fragment (V_L-V_H) CD8-hinge-TM 4-1BB CD3z 2A-CD34t

Figure S7. Constructs. Related to Figure 2. Protein sequence of CD19-, CD37- and CD33CAR used in this study.

Figure S8. CD37CAR activity

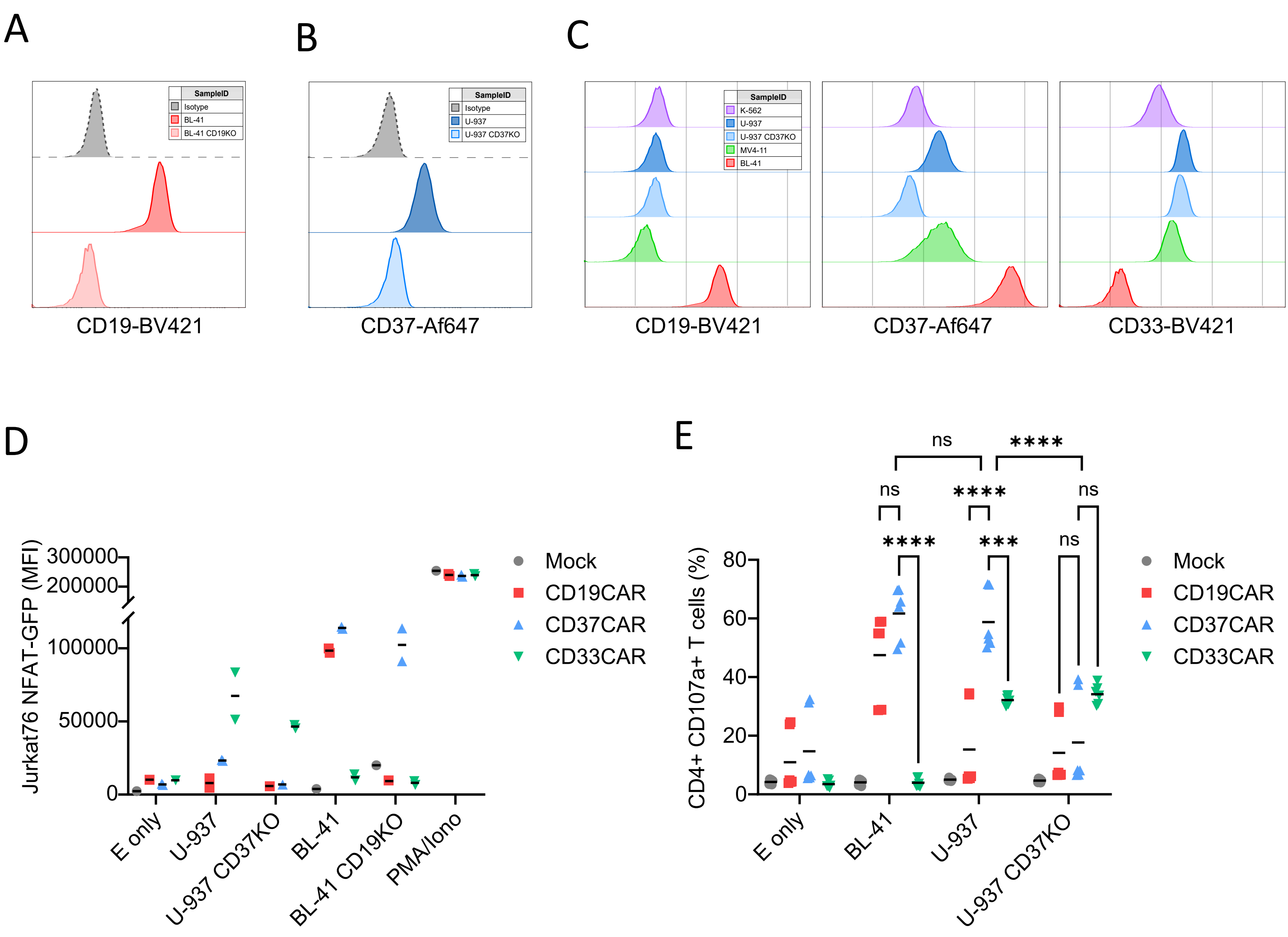

Figure S8. CD37CAR activity. Related to Figure 2. (A) Representative detection of CD19 on BL-41 and BL-41 CD19 knock-out (KO). The dotted line represents the corresponding isotype control. (B) Representative detection of CD37 on U-937 and U-937 CD37KO. The anti-CD37 mAb clone HH1 was used for detection. The dotted line represents the corresponding isotype control. (C) Comparative detection of CD19, CD37 and CD33 on K-562, U-937, U-937 CD37KO, MOLM-13, MV4-11, and BL-41. The anti-CD37 mAb clone HH1 was used for detection. Representative detection of geometric median fluorescent intensity of GFP (D) (NFAT-GFP activation signal) of Jurkat76 cells transduced with Mock, CD19-, CD37- or CD33CAR and co-cultured for 24 hours with the indicated cell lines or left alone (E only). E:T = 1:2 (2 independent experiments, mean). Percentage (E) of CD4+ CD107a+ T cells upon 6 hours of co-culture with the indicated cell lines or left alone (E only). E:T = 1:2 (n = 3 donors in duplicates, mean). Two-way ANOVA followed by Tukey's multiple comparison tests. Comparisons versus CD37CAR are displayed.

Figure S9. Cytokine release

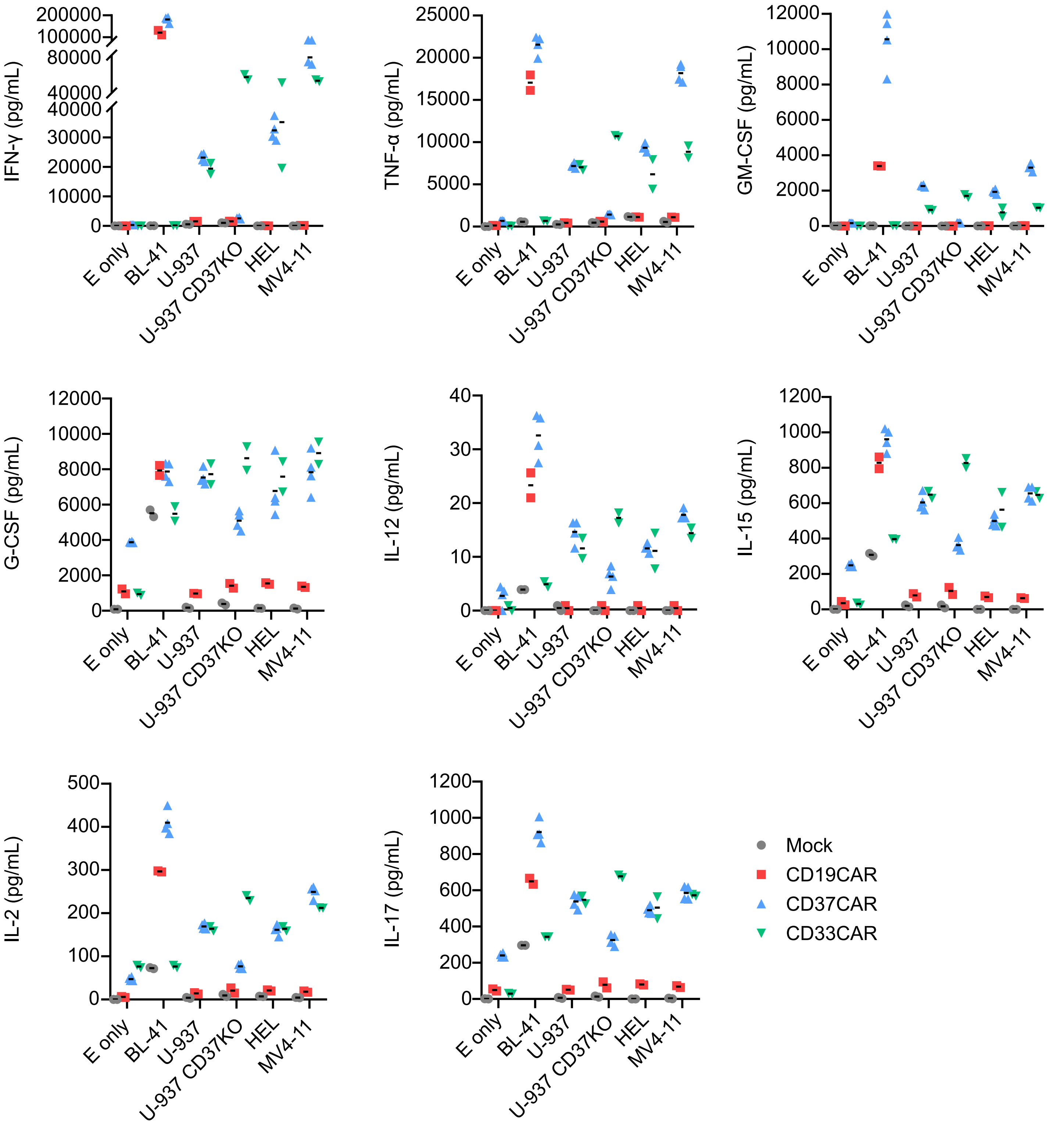

Figure S9. Cytokine release. Related to Figure 2. Secretion (pg/mL) of IFN-γ, TNF-α, GM-CSF, G-CSF, IL-2, IL-12, IL-15, and IL-17 in the supernatant of T cell co-culture with the indicated cell lines or left alone (E only) after 24 hours. E:T = 1:2 (n = 2 donors except CD37CAR n = 4, mean).

Figure S10. Weight of mice

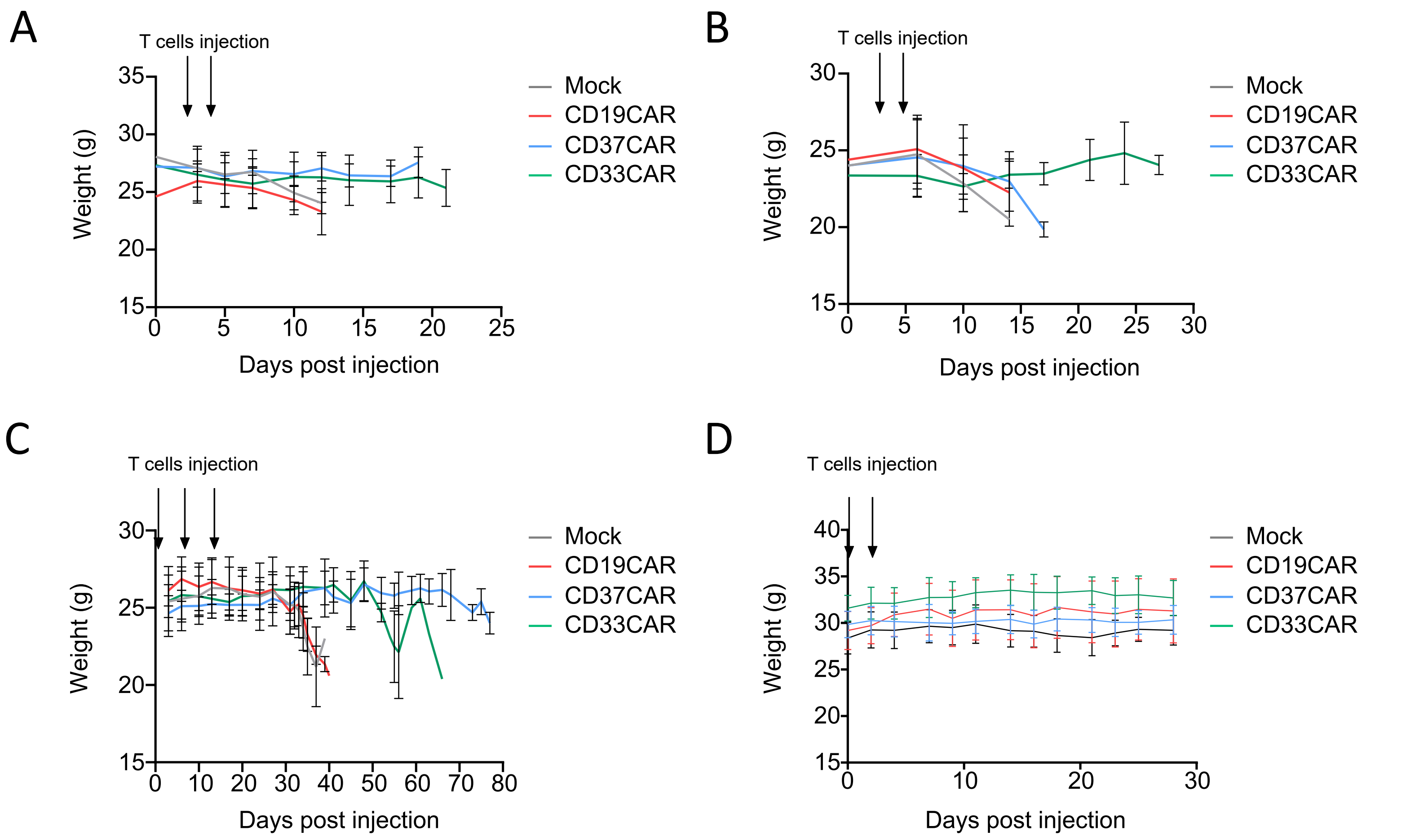

Figure S10. Weight of mice. Related to Figures 4 and 5. (A) Representation of the weight monitoring of the U-937 mice model (Figure 4A-D). (n = 5, mean ± SD). (B) Representation of the weight monitoring of the MOLM-13 mice model (Figure 4E-H). (n = 5, mean ± SD). (C) Representation of the weight monitoring of the AML-PDX mice model (Figure 5). (n = 7, mean ± SD). (D) Representation of the weight monitoring of the AML-PDX mice model (Figure S11 C-G). (n = 8, mean ± SD).

Figure S11. In vivo models

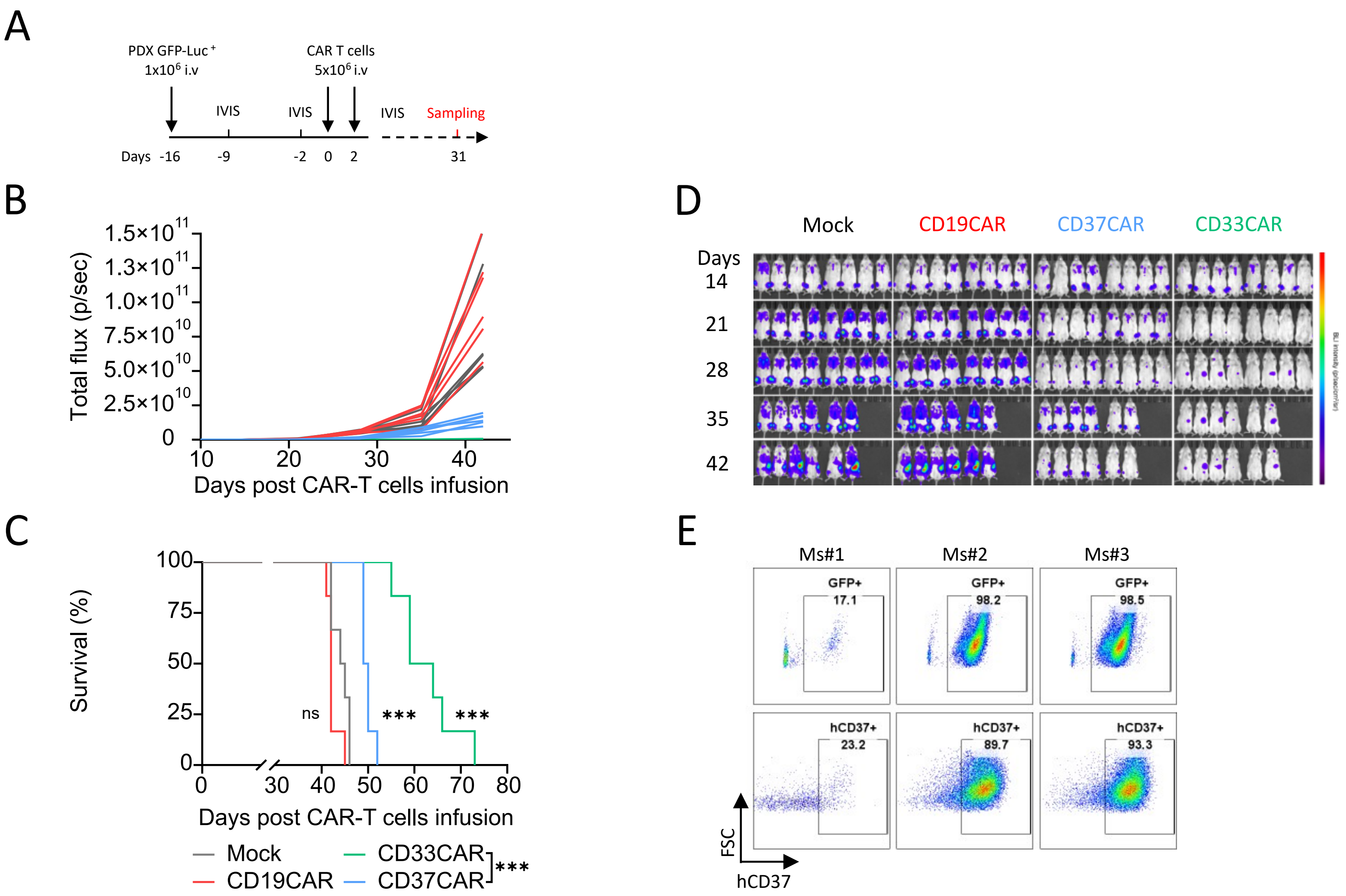

Figure S11. In vivo model. Related to Figure 5. (A) Schematic representation of the 1st PDX in vivo experiment. 1x106 AML-PDX GFP-Luc+ cells were inoculated intravenously (i.v) in NSG mice 16 days prior T cell injection. IVIS was performed 2 days before T cell injection to confirm tumour establishment and randomize the mice. 5x106 Mock, CD19-, CD37- or CD33CAR T cells were injected i.v on day 0 and 2. The % of CAR -expressing population was adjusted between the groups to 60% using Mock cells. Tumor growth was tracked weekly using IVIS. 3 mice were sampled per group on day 31 to analyze recurrent AML cells. (B) Bioluminescence kinetics of the AML-PDX GFP-Luc+ cells growth in NSG mice treated with CAR T cells (n = 8 mice per group). (C) Kaplan-Meier survival curves of NSG mice-bearing AML-PDX GFP-Luc+ cells and treated with CAR T cells (n = 8 mice per group). Comparisons of survival curves were determined by log-rank test.(D) Representative bioluminescence images of (B) and (C). (E) Detection of CD37 (mAb clone HH1) on AML-PDX GFP-Luc+ cells sampled from the bone marrow on day 31 (n = 3 mice of CD37CAR group).

Figure S12. CD37CAR T cells treatment with dasatinib

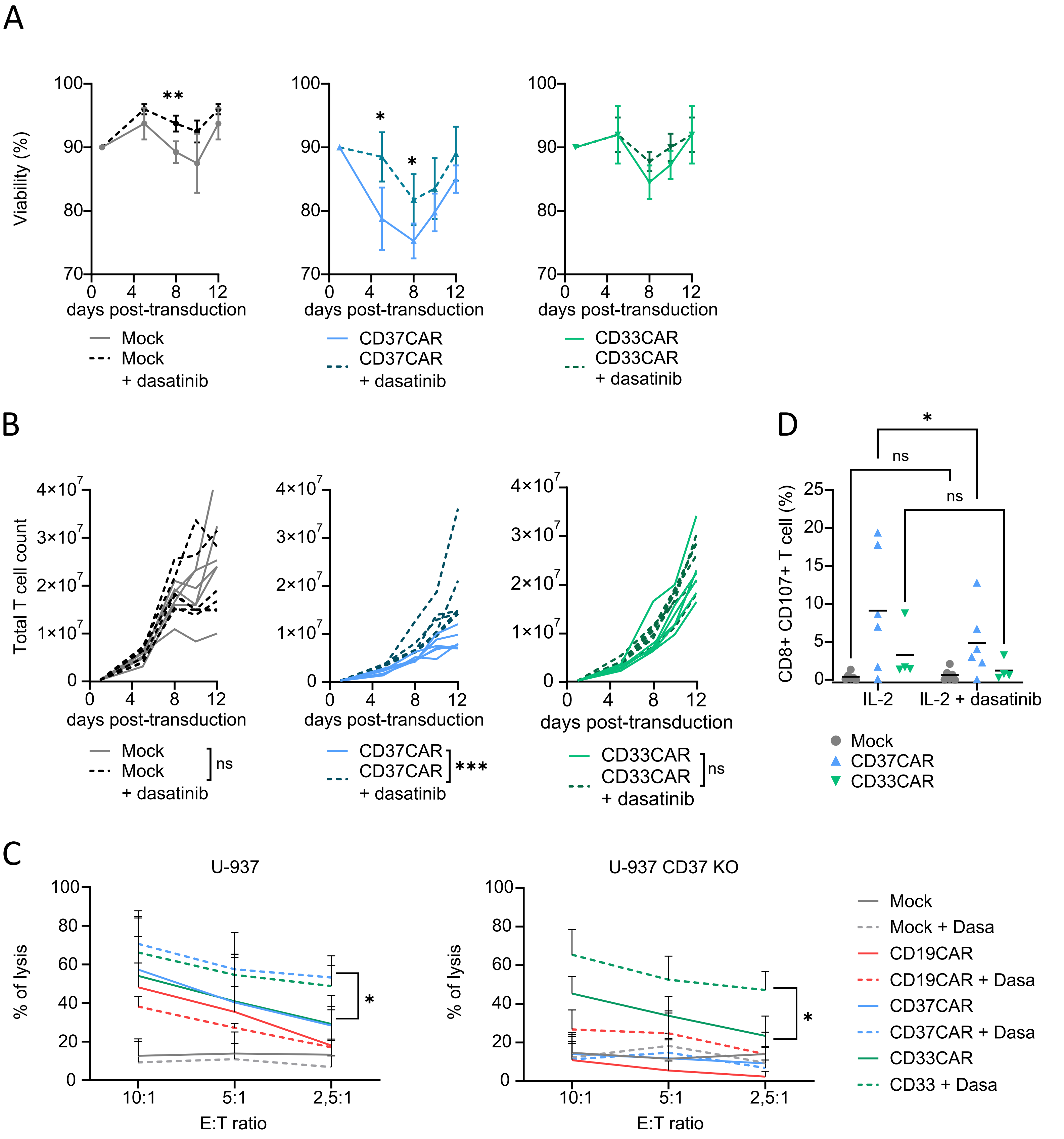

Figure S12. CD37CAR T cells treatment with dasatinib. Related to Figure 5. Viability (A) and expansion in total T cell count (B) of T cell donors-bearing the CAR constructs with or without dasatinib for 12 days post-transduction. (A) Paired t-test was used for statistical analysis (n = 4 donors, mean ± SD). (B) Two-way ANOVA followed by Tukey's multiple comparisons tests (n = 6 donors). Stats are displayed for day 12, as result of the expansion. (C) Specific cytotoxicity of CAR constructs (manufactured either with or without dasatinib for 12 days post-transduction) incubated 6 hours with U-937 and U-937 CD37KO. E:T ratios indicated (n = 6 donors,mean ± SD). Two-tailed paired Student's t-test.
(D) Percentage of CD8+ CD107a+ T cells at 10 days of expansion with or without dasatinib (n = 6 donors, mean). Two-way ANOVA followed by Sifak's multiple comparisons tests.

Figure S13. The resurgence of AML cells in the PDX animal is not due to antigen escape

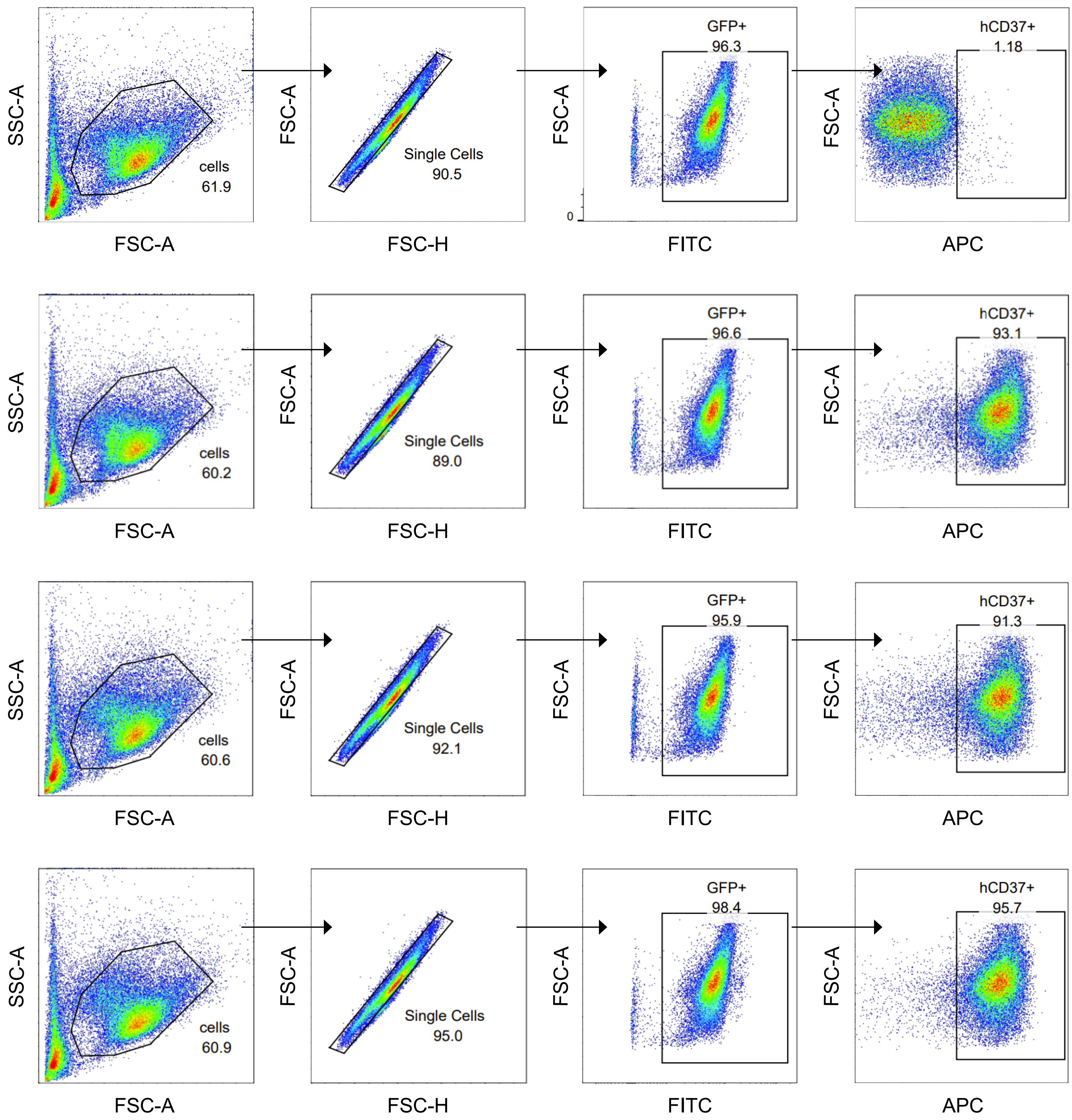

Figure S13. The resurgence of AML cells in the PDX animal is not due to antigen escape. Related to Figure 5. Human AM PDX cells were recovered from mouse spleen and analysed for CD37 expression. First raw: Gating strategy and Isotype control staining of isolated human cells from mouse spleen. Second raw: Gating strategy and CD37 staining of AML PDX cells from mouse 1. Third raw: Gating strategy and CD37 staining of AML PDX cells from mouse 2. Fourth raw: Gating strategy and CD37 staining of AML PDX cells from mouse 3.

Supplementary Table 1. AML patient cohort. Related to Figure 1.

Patient nr	Age	WBC at diagnosis	Diagnosis	De novo/sAML	FAB	ELN 2017 risk	Treatment	CR/nonCR	TX-status	5-year survival (months)	CD37 myeloid cells
**1**	35-60		AML	De novo	M5	I	STD	CR	Allo	>107	Low
**4**	35-60	57.8	AML	De novo	M5	I	STD	CR		6	Low
**5**	>60		MDS		M1	A	no treatment		Allo		Low
**6**	<35		AML	De novo	M4	F	STD	CR	Allo	>84	Low
**7**	35-60		AML	De novo	M4	I	unknown status	CR	Allo		Low
**8**	>60	102	CMML		M4	A	no treatment		Auto		High
**9**	>60		AML	De novo	M0	A	no treatment		Auto		Low
**11**	35-60	37.4	AML	De novo	M5	I	STD	CR	Allo	>144	Low
**12**	35-60		AML	De novo	M2	I	STD	nonCR	Allo	>144	Low
**13**	35-60		AML	De novo	M5	F	STD	CR	Allo	27	Low
**14**	35-60		AML	De novo	M5	I	STD	nonCR		>118	Low
**15**	35-60		AML	De novo	M0	I	STD	CR	Allo	73	High
**16**	>60	101	AML	De novo	M5	A	STD	CR	Allo	>103	Low
**17**	>60		AML	De novo	M5	I	STD	CR	Auto	>92	Low
**18**	35-60		AML	De novo	M1	F	STD	CR		>98	Low
**19**	35-60		AML	De novo	M1	I	unknown status			26	Low
**20**	35-60		AML	De novo	M5	I	STD	CR		7	High
**21**	35-60		AML	De novo	M2	I	STD	CR	Allo	27	Low
**22**	>60		AML	De novo	nt	A	no treatment				High
**23**	>60	29.8	AML	De novo	M1	I	STD	nonCR	Allo	22	Low
**24**	>60	214	AML	De novo	M5	I	STD			1	Low
**25**	35-60	241	AML	De novo	M2	I	STD	CR		>82	Low
**27**	35-60		AML	De novo	M4	F	STD	CR	Auto	>66	Low
**29**	>60		AML	De novo	M1	A	no treatment				High
**30**	>60	22.3	AML	De novo	M2	A	unknown status		Allo		High
**31**	>60	191	AML	De novo	nt	I	no treatment				High
**32**	>60	67	AML	De novo	M1	A	STD	CR	Allo	8	High
**33**	35-60	65.4	AML	De novo	M4	F	STD	CR		14	Low
**35**	>60	12.7	CMML		M5	A	no treatment		Allo	6	High
**36**	>60	82.3	AML	De novo	M5	I	STD	CR		1	Low
**38**	35-60	78	AML	tAML	M3	F	APL specific treatment	CR			High
**39**	>60	12.6	AML	De novo	M4	I	STD	CR		6	High
**40**	>60		AML	De novo	M1	A	no treatment		Allo		Low
**41**	>60	105	MDS		M4	I	no		NA		Low

							treatment				
**42**	35-60	46	AML	De novo	M1	F	unknown status				Low
**43**	>60		CMML		M5	I	no treatment				Low
**43**	>60	101	AML	Residiv	M2	I	no treatment				High
**46**	<35	111	AML	De novo	M5	I	STD	CR		>102	Low
**47**	>60	44.7	AML	De novo	M5	A	STD	CR		16	High
**48**	35-60		CMML		M5	A	no treatment				Low
**49**	35-60	96.9	AML	De novo	M2	I	STD	nonCR		29	Low
**50**	>60	65.3	AML	De novo	M1	I	STD	CR		5	High
**51**	>60	92	MF		M1	I	no treatment				High
**52**	>60	23	AML	De novo	M0	I	no treatment				Low
**53**	35-60		AML	De novo	M2	I	STD	CR		24	Low
**54**	>60		AML	De novo	M0	A	no treatment				Low
**55**	35-60	28.2	AML	De novo	M1	F	STD	CR		>80	Low
**56**	>60	nt	MDS		M2	I	unknown status			1	Low
**57**	35-60	34.5	AML	De novo	M4	I	STD	CR		>80	High
**58**	>60	47.5	AML	De novo	M4	F	unknown status				Low
**59**	>60	22.6	AML	De novo	M4	I	STD	CR		14	Low
**60**	>60	37.2	AML	De novo	M5	F	STD	CR		8	Low
**61**	>60	23.1	AML	De novo	M2	I	STD	nonCR		15	High
**63**	35-60	17.3	AML	De novo	M2	A	STD				Low
**64**	>60	71.4	AML	De novo	M2	F	no treatment				Low
**66**	>60	145	AML	De novo	M2	I	no treatment	CR			High
**67**	35-60	72.4	AML	De novo	M2	I	STD	CR		>75	Low
**68**	>60	5.6	AML	De novo	M1	I	STD	CR			Low
**69**	>60	nt	MF		M2	nt	no treatment				High

Supplementary Table 2. Mass Cytometry antibody panel for 59 AML patients and 5 healthy donors. Related to the STAR Methods, mass cytometry section.

Plus 16 bleed	Tag	Antibody	Clone	Used for Clustering	Comments
105	89 Y	CD45	HI30	x	Human Leukocytes
127	111 Cd	CD3	UCHT1	x	T cells
128	112 Cd	CD34	581	x	Hematopoietic stemcells and progenitors
129	113 Cd	CD123	6H6	x	AML blasts, basophils
130	114 Cd	CD7	2A3	x	HSC, NK cells and T cells, myeloblasts
132	116 Cd	HLA-DR	L243	x	AML blasts
155	139 La	CD8a	RPA-T8	x	T cell marker
157	141 Pr	CD19	HIB19	x	Activated B cells
158	142 Nd	Caspase 3 (Cleaved)	D3E9		Dying cells
159	143 Nd	CD45RA	Hi100	x	Naive T cells
160	144 Nd	CD38	HIT2	x	Basophils high, HSC low
161	145 Nd	CD4	RPA-T4	x	T cell marker, T helper cells, Monocytes and macrophage
162	146 Nd	CD64	10,1	x	Monocytes
163	147 Nd	pStat5 [Y694]	47		NRAS downstream
164	148 Nd	CD16	3G8	x	Granulocytes and NK cells
166	150 Nd	pRB (S807/811)	J112-906		phosphorylated retinoblasoma protein allowing cell cycle progression
168	152 Sm	CD66b	8OH3		Granulocytes
169	153 Eu	RUNX	EPR3099		AML biomarker
170	154 Sm	NRAS	EPR20278		AML biomarker
171	155 Gd	CD56	B159	x	NK cells and T lymphocyte
172	156 Gd	CD37	HH1		Potential AML biomarker, very positive in B cells.
174	158 Gd	CD33	WM53	x	Myeloid lineage,
175	159 Tb	CD90	5E10	x	HSC and MSC
176	160 Gd	CD14	M5E2	x	Monocytes
177	161 Dy	FLT3	S-18		Hematopoietic precursor marker, Common AML biomarker
178	162 Tm	Ki67	B56		Proliferation marker
179	163 Dy	CD105	43A3		MSC marker
180	164 Dy	Cyclin B1	GNS-1		G2/mitotic specific
181	165 Ho	CD45RO	UCHL1	x	Leukocyte common antigen, T cell activation
182	166 Er	CD44	x	x	Monocytes, T cells and ALML
183	167 Er	pErk 1/2 [T202/Y204]	D13.14.4		MAPK/ERK pathway, downstream of RAS
184	168 Er	cKit	YB5.B8		HSC and some AML
185	169 Tm	CD25	2A3	x	Activated T cells, Activated B cells, Myeloid precursor cells
186	170 ER	CSF1R (intracellular C-term)	9-4D2-1E4		Macrophages
188	172 Yb	CD73	EPR6115		MSC, fibroblast
189	173 Yb	CD300e	233810	x	Myeloid cells, activating receptor
190	174 Yb	CD11c	L243	x	Monocytes, macrophages and B cells, myeloblast
192	176 Yb	CD95/FAS	DX-2		Fatty acid synthesis, AML biomarker
225	209 Bi	CD11b	ICRF44	x	Neutrophils, NK cells and macrophage

Supplementary Table 3. AML and healthy cell description. Related to Figure 1.

Short	Abbreviation
HSC	Hematopoietic stem cell
MPP	Multipotential progenitors
CMP	Common myeloid progenitor cell
GMP	Granulocyte monocyte progenitors
MEP	Megakaryocyte-erythroid progenitor cell
early_PM	Early Promyelocyte
MM	Metamyelocytes
MY	Myelocyte
Mono	Monocytes
PMN	Polymorphonuclear cells
Normal	AML with Normal karyotype
Complex	AML with Complex karyotype
inv(16)	AML with inv(16)
t(15;17)	AML with t(15;17)
t(8;21)	AML with t(8;21)
t(11q23)/MLL	AML with t(11q23)/MLL
MDS	MDS
nan	AML with no karyotype information
Trisomy 8	AML with Trisomy 8
del(5q)	AML with del(5q)
del(7q)/7q-	AML with del(7q)/7q-
t(9;11)	AML with t(9;11)
Other	AML with Other abnormalities
7	AML with +7
Complex_ del(5q)	AML with Complex del(5q)
Complex_ untypical	AML with Complex untypical karyotype
ALL	ALL
inv(3)	AML with inv(3)
trisomy 11	AML with trisomy 11
trisomy 13	AML with trisomy 13
t(6;9)	AML with t(6;9)
t(8;16)	AML with t(8;16)
del(9q)	AML with del(9q)
t(1;3)	AML with t(1;3)
-5/7(q)	AML with -5/7(q)
-9q	AML with -9q
8	AML with +8
t(9;22)	AML with t(9;22)
abn(3q)	AML with abn(3q)

Supplementary Table 4. CyTof antibody panel used for the phenotypic characterization of the CAR T cell population. Related to Figure 5.

Tag	Antibody Target	Clone	Target significance	Concentration
89Y	hCD45	HI30	human leukocytes	200
111Cd	CD4	RPA-T4	CART T helper cells	2 µg/mL
113Cd	CD8	HIT8a	CART Cytotoxic T cells	0.25 µg/mL
116Cd	CD57	HNK-1	T cell differentiation, senescence	0.5 µg/mL
141Pr	CD196	G034E3	CART Homing/polarization	100
142Nd	OX40	ACT35	CART Activation	100
143Nd	Biotin	1D4C5	CD19 and CD37 CART cells	200
144Nd	CD38	HIT2	AML	100
145Nd	trCD34	4H11	CD33 CART cells	2 µg/mL
146Nd	GFP	FM264G	Luc+ PDX1 AML cells	6 µg/mL
147Sm	mCD45	30F11	mouse leukocytes	1600
148Nd	ICOS	C398.4A	CART Activation	100
149Sm	CD45RO	UCHL1	CART Effector/Memory	400
150Nd	LAG-3	11C3C65	CART Exhaustion	100
151Eu	CD123	6H6	AML	100
152Sm	Fas	DX2	CART Effector/Memory	200
153Eu	CD45RA	HI100	CART Effector/Memory	400
154Sm	TIM-3	F38-2E2	CART Exhaustion	100
155Gd	PD-1	EH12.2H7	CART Exhaustion	100
156Gd	CD37	HH1	AML (clone HH1)	10 µg/mL
158Gd	CD33	WM53	AML	100
159Tb	TIGIT	MBSA43	CART Exhaustion	100
160Gd	CD28	CD28.2	CART Activation	200
161Dy	CTLA-4	14D3	CART Exhaustion	200
162Dy	CD27	L128	CART Effector/Memory	400
164Dy	CD185	RF8B2	CART Homing/polarization	100
165Ho	CD19	HIB19	negative control	1000
167Er	CD197	G043H7	CART Effector/Memory	100
168Er	CD127	A019D5	CART Homing/polarization	80
169Tm	CD25	2A3	CART Activation	500
170Er	CD3	UCHT1	CART all T cells	400
171Yb	CD44	IM7	almost all cells	1600
172Yb	Ki-67	B56	CART proliferation	200
173Yb	4-1BB	4B4-1	CART Activation	200
174Yb	HLA-DR	L243	CART Activation	200
175Lu	CD184	12G5	CART Homing/polarization	100
176Yb	Histone 3	D1H2	all cells	100
209Bi	CD11b	ICRF44	AML	150
