# Supplementary material for: CD37 is a safe chimeric antigen receptor target to treat acute myeloid leukemia
Source: Cell Rep Med. 2024 May 15;5(6):101572. doi: 10.1016/j.xcrm.2024.101572 (PMC11228397; doi:10.1016/j.xcrm.2024.101572)
Supplement: Document S2. Article plus supplemental information [file mmc2.pdf]

Cell Reports Medicine

Article

CD37 is a safe chimeric antigen receptor target to treat acute myeloid leukemia

Graphical abstract

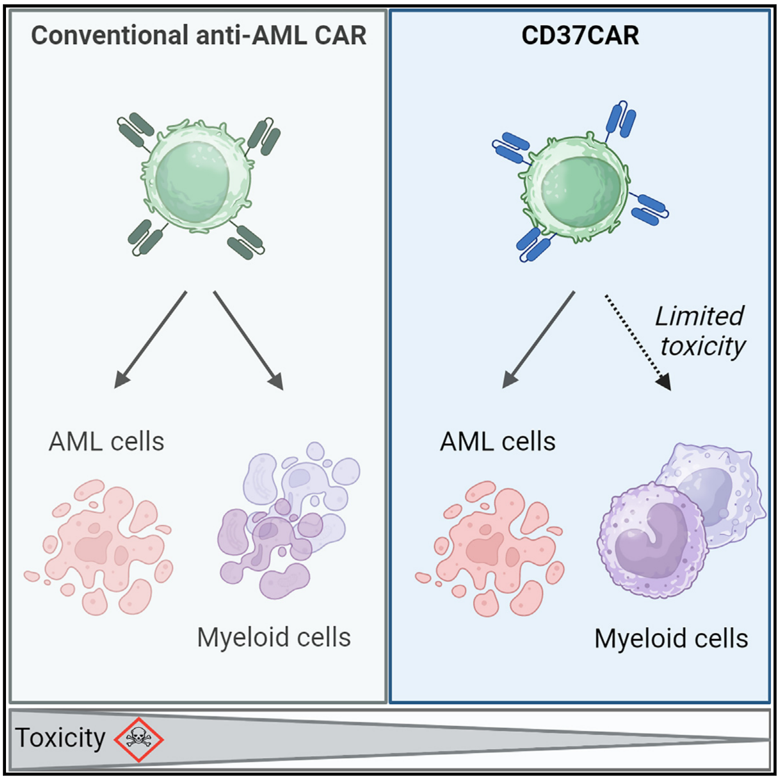

Highlights

- CD37 is expressed on AML blasts
- CD37 expression correlates with ELN 2017 risk stratification
- CD37CAR T cells are efficient against AML *in vitro* and *in vivo*
- CD37CAR T cells do not deplete myeloid progenitors

Authors

Benjamin Caulier, Sandy Joaquina, Pascal Gelebart, ..., Emmet McCormack, Else Marit Inderberg, Sébastien Wälchli

Correspondence

sebastw@rr-research.no

In brief

Caulier et al. demonstrate that CD37, a B cell marker, is expressed on the cell surface of the majority of primary AML samples. A CAR construct directed against CD37 controls AML progression, suggesting that CD37CAR T cells represent a promising solution for AML treatment.

Caulier et al., 2024, Cell Reports Medicine *5*, 101572
June 18, 2024 © 2024 The Author(s). Published by Elsevier Inc.
https://doi.org/10.1016/j.xcrm.2024.101572

CellPress

Cell Reports Medicine

CellPress
OPEN ACCESS

Article

CD37 is a safe chimeric antigen receptor target to treat acute myeloid leukemia

Benjamin Caulier,[1,2,3] Sandy Joaquina,[1,15] Pascal Gelebart,[4,6,15] Tara Helén Dowling,[4,5,6] Fatemeh Kaveh,[1] Moritz Thomas,[7,8] Luka Tandaric,[6,9] Patrik Wernhoff,[1] Niveditha Umesh Katyayini,[2,3] Cara Wogsland,[4,6] May Eriksen Gjerstad,[4,6] Yngvar Fløisand,[2] Gunnar Kvalheim,[1] Carsten Marr,[7] Sebastian Kobold,[10,11,12] Jorrit M. Enserink,[2,3,13] Bjørn Tore Gjertsen,[6,14] Emmet McCormack,[4,5,6] Else Marit Inderberg,[1] and Sébastien Wälchli[1,16,*]

[1]Translational Research Unit, Section for Cellular Therapy, Department of Oncology, Oslo University Hospital, Oslo, Norway
[2]Institute for Cancer Research, Department of Molecular Cell Biology, Oslo University Hospital, Oslo, Norway
[3]Center for Cancer Cell Reprogramming (CanCell), Institute for Clinical Medicine, Faculty of Medicine, University of Oslo, Oslo, Norway
[4]Department of Clinical Science, Precision Oncology Research Group, University of Bergen, 5021 Bergen, Norway
[5]Centre for Pharmacy, Department of Clinical Science, University of Bergen, Bergen, Norway
[6]Centre for Cancer Biomarkers (CCBIO), University of Bergen, Bergen, Norway
[7]Institue of AI for Health, Helmholtz Munich, 85764 Neuherberg, Germany
[8]School of Life Sciences Weihenstephan, Technical University of Munich, Freising, Germany
[9]Department of Obstetrics and Gynecology, Haukeland University Hospital, Bergen, Norway
[10]Division of Clinical Pharmacology, Department of Medicine IV, University Hospital, Ludwig-Maximilians-Universität München, Munich, Germany
[11]German Center for Translational Cancer Research (DKTK), Partner Site Munich, Munich, Germany
[12]Einheit für Klinische Pharmakologie (EKLiP), Helmholtz Zentrum München, Research Center for Environmental Health (HMGU), Neuherberg, Germany
[13]Section for Biochemistry and Molecular Biology, Faculty of Mathematics and Natural Sciences, University of Oslo, Oslo, Norway
[14]Department of Medicine, Hematology Section, Haukeland University Hospital, Bergen, Norway
[15]These authors contributed equally
[16]Lead contact
*Correspondence: sebastw@rr-research.no
https://doi.org/10.1016/j.xcrm.2024.101572

SUMMARY

Acute myeloid leukemia (AML) is characterized by the accumulation of immature myeloid cells in the bone marrow and the peripheral blood. Nearly half of the AML patients relapse after standard induction therapy, and new forms of therapy are urgently needed. Chimeric antigen receptor (CAR) T therapy has so far not been successful in AML due to lack of efficacy and safety. Indeed, the most attractive antigen targets are stem cell markers such as CD33 or CD123. We demonstrate that CD37, a mature B cell marker, is expressed in AML samples, and its presence correlates with the European LeukemiaNet (ELN) 2017 risk stratification. We repurpose the anti-lymphoma CD37CAR for the treatment of AML and show that CD37CAR T cells specifically kill AML cells, secrete proinflammatory cytokines, and control cancer progression *in vivo*. Importantly, CD37CAR T cells display no toxicity toward hematopoietic stem cells. Thus, CD37 is a promising and safe CAR T cell AML target.

INTRODUCTION

Acute myeloid leukemia (AML) is an aggressive blood cancer that remains difficult to treat.[1] The first line of treatment against AML relies on repeated high-dose chemotherapy frequently consolidated with allogeneic hematopoietic stem cell transplantation (HSCT). Improved knowledge of leukemia genetics has led to a recent update of the European LeukemiaNet (ELN) risk classifications, response criteria, and therapy guidelines.[2,3] First-line and relapse therapy are constantly developed for AML and include antibody-drug conjugate (ADC) gemtuzumab ozogamicin targeting CD33; monoclonal antibodies (mAbs) targeting CD44; and CD123, bi-specific T cell engagers (BiTEs) or immune checkpoint inhibitors.[4–7] Unfortunately, 50% of AML patients relapse after initial remission.[8] This situation is presumably due to the presence of a persisting population of leukemic stem cells (LSCs), which are known to initiate and maintain the disease by exhibiting properties of self-renewal, cell cycle quiescence, and chemo-resistance.[9] The outcome of patients with relapsed/refractory (R/R) AML is particularly dismal, with no more than 10% of overall survival (OS) at 3 years.[2,10] Recently, adoptive cellular therapies (ACTs) with T cells expressing chimeric antigen receptors (CARs) have been investigated in R/R AML. Some of these CARs showed promising pre-clinical results by targeting the myeloid cell-restricted CD33[11,12] and stem cell marker interleukin (IL)-3 receptor (CD123)[13] as well as other

© 2024 The Author(s). Published by Elsevier Inc.
This is an open access article under the CC BY license (http://creativecommons.org/licenses/by/4.0/).

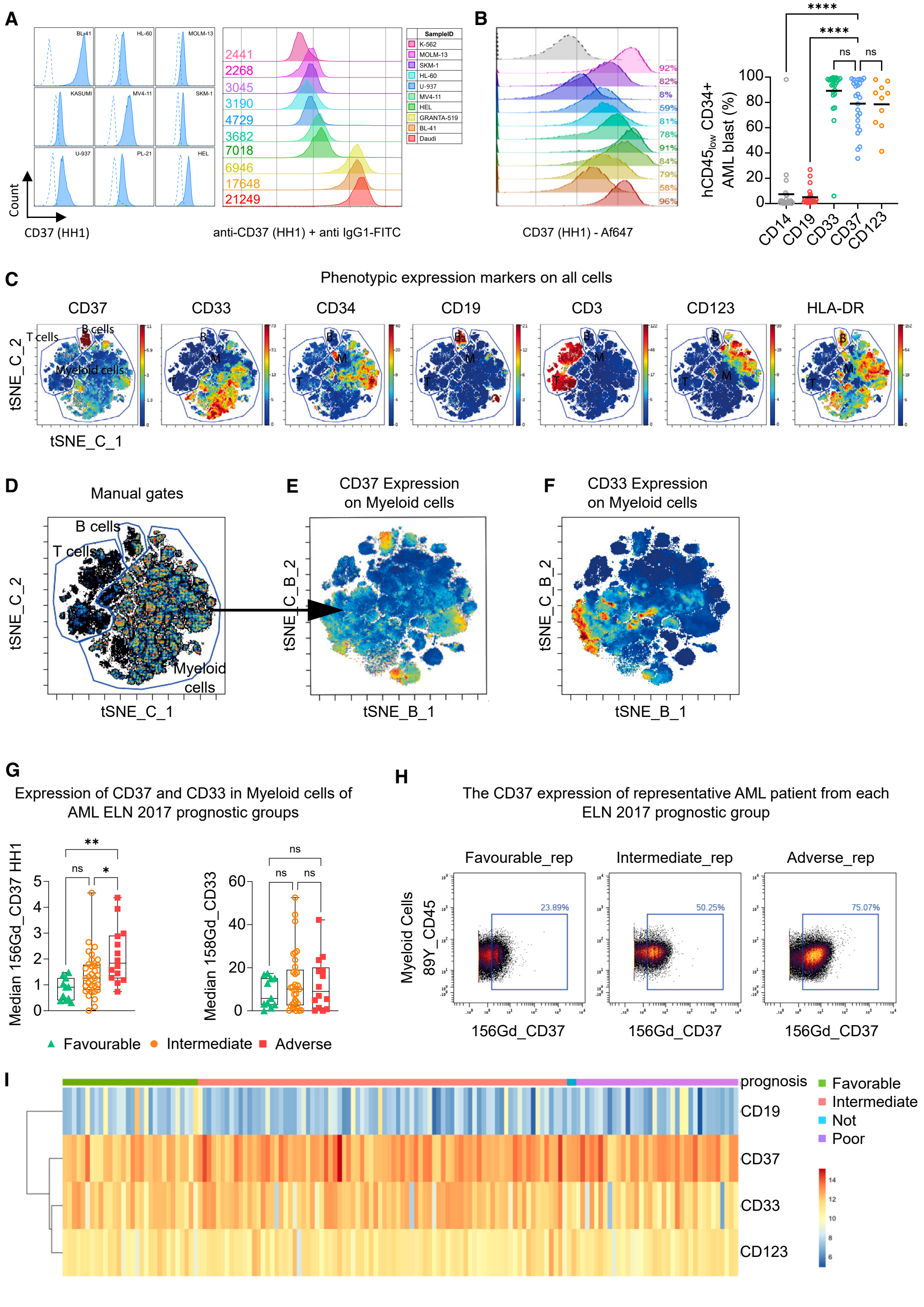

(legend on next page)

leukemia-associated antigens. CD33 and CD123 are currently the main targets under evaluation in clinical trials.[7,14] Combinatorial approaches to prevent disease relapses associated with antigen loss, as observed in B cell malignancies,[15,16] are also being tested. However, the main challenge of AML CAR T therapy is that myeloblasts frequently share target expression with healthy hematopoietic stem cells (HSCs), or targets are also broadly expressed outside the hematopoietic system,[7] contributing to the off-tumor toxicity of the CAR T cell therapy. There is a strong need to develop safer CAR designs against R/R AML to achieve objective long-term remission.

CD37 is a cell-surface glycoprotein that belongs to the four-transmembrane tetraspanin superfamily,[17] known to interact with a range of adhesion molecules, growth and signaling receptors, as well as other tetraspanins, all of which are involved in membrane organization, signal transduction, survival, and apoptosis.[17–19] Unlike other broadly distributed tetraspanins (e.g., CD9, CD81, CD151), CD37 expression appears restricted to B cells, although it has been reported at far lower levels in other healthy hematopoietic cells.[20–25] In contrast, CD37 expression in cancer has been demonstrated in B cell non-Hodgkin lymphoma (B-NHL),[26] but it was recently observed that CD37 expression is not a hallmark of all B cell malignancies; some recent studies demonstrated that only 60% of follicular lymphoma (FL)[27] and only 40% of diffuse large B cell lymphoma (DLBCL)[28] tested positive. In addition, CD37 was also detected in T cell lymphoma,[24,29] whereas one report detected it in AML at the protein level[24] and another showed increased mRNA level in AML patient samples.[30] Finally, CD37 is druggable and several antibody-based molecules have been exploited,[20,24,31–35] mainly to treat B-NHL.

In the present work, we undertook a deeper study of CD37 expression in AML samples covering the full spectrum of the disease and detected CD37 in the majority of AML patients. We observed that, in contrast to CD33, CD37 protein expression showed an excellent correlation with the ELN 2017 patient prognostic stratification. The HH1 anti-CD37 antibody-based CAR (CD37CAR), previously tested in B-NHL,[32] was tested against AML and shown to be of comparable efficiency to the CD33CAR, with the advantage of being less toxic against healthy cells. The present data pre-clinically validate CD37 as a safe and efficient target for CAR T cell therapy in AML.

RESULTS

CD37 is expressed in AML patient samples

We tested the presence of CD37 at the cell surface using two different anti-CD37 antibodies (Figure S1A). When the anti-CD37 antibody M-B371 was used on the pro-monocytic myeloid leukemia cell line, U-937, a negative-to-weak signal was observed. However, when the cells were stained with the anti-CD37 HH1 antibody,[36] a clear binding was detected. The difference between the antibodies was much less marked when staining B cell lymphoma. In order to test the possibility that HH1 recognized another protein at the surface of AML cells, we generated a U-937 knockout for CD37, U-937^{CD37KO}, and observed no binding of HH1 (Figures S1A and S1B). These data suggest that the anti-CD37 HH1 antibody was much more sensitive to the CD37 protein expressed on AML cells. We then tested whether a difference in the three main isoforms of CD37 could affect the recognition. First, we studied their expression in AML, and although the distribution of mRNA isoforms was different between normal and AML bone marrow (BM), all isoforms were expressed (Figure S1C). Nevertheless, when we overexpressed these isoforms in HEK cells, both HH1 and M-B371 antibodies' recognition was restricted to the isoform 1 (Figures S1D–S1E). Second, we tested whether the glycosylation status of CD37 might interfere with antibody recognition, but treatment of target cells with neuraminidase altered neither its recognition nor CAR activity (Figures S1F and S1G). We confirmed the ability of HH1 to detect CD37 on different AML cell lines (Figure 1A left) and quantified CD37 antigen density (Figure 1A right). CD37^{high} B cell lymphoma BL-41 and

Figure 1. CD37 expression on AML

(A) CD37 staining of different AML cell lines and BL-41 (B cell lymphoma) HH1 antibody. The dotted line represents the corresponding murine IgG1 isotype control (left). Surface CD37 protein quantification of K-562 (CML), U-937, MV4-11, MOLM-13, HEL (AML), and BL-41 (B cell lymphoma) (right). The murine IgG1 anti-CD37 mAb clone HH1 was used for the detection. A murine IgG1 isotype was used to set the background. The numbers indicate the amount of CD37 molecules per cell.

(B) CD37 staining in primary samples. (Left) Percentage of hCD45$^+$ CD37$^+$ cells in primary AML samples used as PDX models (n = 11). The anti-CD37 HH1 was used, and the dotted line represents the isotype control. (Right) Percentage of CD14, CD19, CD33, CD37, and CD123 surface proteins on hCD45$_{low}$ CD34$^+$ AML blast population from primary BM samples (n = 25). Black bar represents the mean. One-way ANOVA followed by Dunnett's multiple comparison tests is displayed, ****$p < 0.0001$, ns = not significant.

(C) Expression of CD37, CD33, CD34, CD19, CD3, CD123, and HLA-DR are shown as heat dot plot on tSNE-Cuda (tSNE_C) of concatenated AML patients (n = 59). The markers shown are indicative of cell subset , B cells (CD19), T cells (CD3), and AML (CD33, CD34, CD123, and HLA-DR). Manual gates are annotated.

(D) The tSNE-Cuda here with manual gates drawn guided by CD3 and CD19. Further analysis was made with the myeloid cell gate.

(E) The expression of CD37 in myeloid cells here shown as heat on the tSNE-Cuda of the myeloid cell gate from (B).

(F) The expression of CD33 in myeloid cells here shown as heat on the tSNE-Cuda of the myeloid cell gate from (D).

(G) The raw median expression intensity of CD37 and CD33 in myeloid cells grouped according to ELN 2017 risk stratification (n = 59). One-way ANOVA multiple comparison test identified that the expression of CD37 was found to be significantly increased for the adverse patient group in comparison to the good (p = 0.0011) and intermediate (p = 0.0247) patient group. No significant correlation of CD33. One-way ANOVA found no significance when investigating the expression of CD33 between ELN 2017 risk groups.

(H) Bi-axial plots of CD45 vs. CD37 expression on myeloid cells of a patient representative from each ELN 2017 risk group showing a 25% stepwise increase between the risk groups. The tSNE-Cuda of the concatenated AML patients (n = 59) using only myeloid cells colored by population annotated as MC.

(I) *CD19*, *CD37*, *CD33*, and *CD123* (*IL3RA*) gene expression analysis from RNA-seq dataset (TCGA-LAML, n = 150). The heatmap shows the normalized RNA-seq counts (DESeq2) for the four genes and each column represents a patient. Patient clustering was performed according to the prognosis (favorable, intermediate, poor).

CD37^{null} chronic myelogenous leukemia K-562 cell lines were used as references (Figure 1A right). AML cell lines displayed generally lower levels of CD37 than B cell lymphomas and required staining with a specific antibody, elucidating the previous conflicting reports on CD37 positivity. We also investigated CD37 expression in several patient-derived xenograft (PDX) models of AML and observed broad expression with an antigen density equivalent to AML cell lines (Figure 1B left and S1H). In a series of patient biopsies ($n = 25$; Figure S2A), we compared the level of CD37 positivity to the validated CAR targets CD33 and CD123 in AML[7] (Figure 1B right; Figures S2B and S2C) and detected similar positivity on the bulk population. Furthermore, the presence of CD37 was detected in AML LSC populations (Figures S2D and S2E). This prompted us to further evaluate CD37 cell-surface expression in a larger and more extensive AML patient cohort, and we examined the CD37 expression using the HH1 antibody in diagnostic peripheral blood samples from 59 AML patients and five healthy donors. These patients were stratified according to ELN 2017 risk classification at diagnosis and complete remission after standard induction therapy was used as an indicator of therapy response/resistance (Table S1). An antibody panel of 40 markers was designed to identify phenotypic cell subsets of the samples including leukemic blasts and healthy populations. The 11 intracellular markers allow for investigation of signaling pathways regulating the differentiation, proliferation, and survival of myeloid cell subsets.[37] From the panel, we used 23 extracellular markers for unsupervised clustering (FlowSOM) and dimensionality reduction (GPU-accelerated implementation of t-distributed stochastic neighbor embedding, tSNE-Cuda) (Figure 1C). As expected, high expression of CD37 was observed in the CD19-expressing B cell population, whereas CD3-expressing T cell populations had no detectable expression of CD37. In contrast, CD37 expression was observed on myeloid cells, and we went on to further investigate the distribution of CD37 in myeloid subsets by FlowSOM and tSNE-Cuda (Figures 1D–1F). The 10 myeloid meta-clusters (MCs) were annotated by application of marker expression and marker enrichment modeling (Figure S3A). They were further manually annotated based on their expression profile and healthy abundance, and relative MC abundance was visualized in a stacked bar plot (Figure S3B) in which healthy donors and patients were ordered based on similar MC distribution patterns (Figure S3C). The MC b_02 was the major MC prevalent in most of the patients and includes the most cells. We investigated the patients' individual CD37 expression levels and discovered significant association of median CD37 expression with the ELN 2017 risk classification (Figures S4A and S4B). Tukey's multiple comparisons test found a significant difference between good and adverse risk groups ($p = 0.0011$), and between intermediate and adverse risk groups ($p = 0.0247$) (Figures 1G and 1H). Furthermore, CD33 protein expression showed no correlation to ELN 2017 risk groups (Figure 1G). Previous data-mining studies have shown a correlation between *CD37* mRNA expression and the French American British (FAB) phenotypic classification system, with the FAB subgroup M5 showing higher expression than the other subgroups.[30] However, in our AML dataset, we found no significant correlation between FAB classification and CD37 median expression (Figure S3D), which could be due to the comparatively small number of samples or a discrepancy between mRNA presence and protein expression.

We used the TCGA-LAML RNA sequencing (RNA-seq) dataset and grouped patients according to the clinical prognostic factors to identify differentially expressed genes ($n = 150$; Figure 1I). To assess the overall similarity between samples, we performed unsupervised clustering and evaluated the expression of *CD19*, *CD33*, *CD37*, and *CD123*. Extracting significantly differentially expressed genes revealed that *CD37* was overexpressed compared to *CD33* and *CD123* ($p = 0.00188$), while, as expected, *CD19* expression levels were much lower ($p = 0.0066$). We further investigated microarray data from the Microarray Innovations in Leukemia (MILE) study[38] ($n = 254$; Figure S5A) and a pool of AML samples[39–41] (high risk, $n = 242$, Figure S5B; and favorable/intermediate risk $n = 1832$, Figures S5C–S5E) and compared CD37 expression to that of hematopoietic stem and progenitor cells (HSPCs) and differentiated myeloid cells from adult healthy BM.[42] Next, we evaluated *CD37* expression using a single-cell RNA-seq dataset consisting of 28,404 healthy and malignant cells from 15 patients diagnosed with AML[43] (Figures S6A and S6B). On a transcriptomic level, *CD37* expression was predominantly restricted to populations of malignant cells (Figure S6C), showing consistent expression among all AML patients exhibiting malignant cellular populations (Figure S6D).

Together, these data confirm that *CD37* mRNA is translated and expressed at the surface of AML cells, but its detection efficiency might only be restricted to some antibodies, thus making CD37 a potential HH1 antibody-based CAR target.

CD37-targeting CAR T cells demonstrate potent activity against AML cell lines

Having established that CD37 is broadly expressed in AML blast cells, we evaluate the potency of CD37CAR T cells against AML, comparing it to anti-CD19 fmc63-based CAR (CD19CAR) as a negative control and to anti-CD33 gemtuzumab ozogamicin-based CAR,[11] CD33CAR, as a validated anti-AML CAR (Figure 2A). Given that the CD33CAR is humanized, we added a truncated CD34 tag to enable its detection (construct sequences provided in Figure S7). We confirmed CD37CAR specificity toward CD37 using a reporter system in which single clones of Jurkat76[44] expressing GFP under the control of NFAT (J76$^{NFAT-GFP}$)[45] were transduced with CD19^{-}, CD37^{-}, or CD33CAR. The CAR-J76$^{NFAT-GFP}$ cells were co-cultured with BL-41 (CD19^{+}, CD37^{high}, CD33^{-}) and U-937 (CD19^{-}, CD37^{low}, CD33^{+}) as well as cell lines in which either CD19 or CD37 was knocked out (Figures S8A–S8C). We observed a strong GFP-positive specific signal from CAR-J76$^{NFAT-GFP}$ cells in the presence of their cognate target (Figures 2B and S8D). Importantly, even the CD37^{low} U-937cell line could induce a specific CD37CAR response, suggesting a strong functional avidity of the construct. Encouraged by these results, we transduced primary T cells from nine healthy donors with the indicated CAR construct (Figures 2C and 2D). In agreement with Okuno et al.,[20] we observed that the viability of CD37CAR T cells decreased by 30%–40% 12 days after transduction (Figure 2E), affecting the

CellPress
OPEN ACCESS

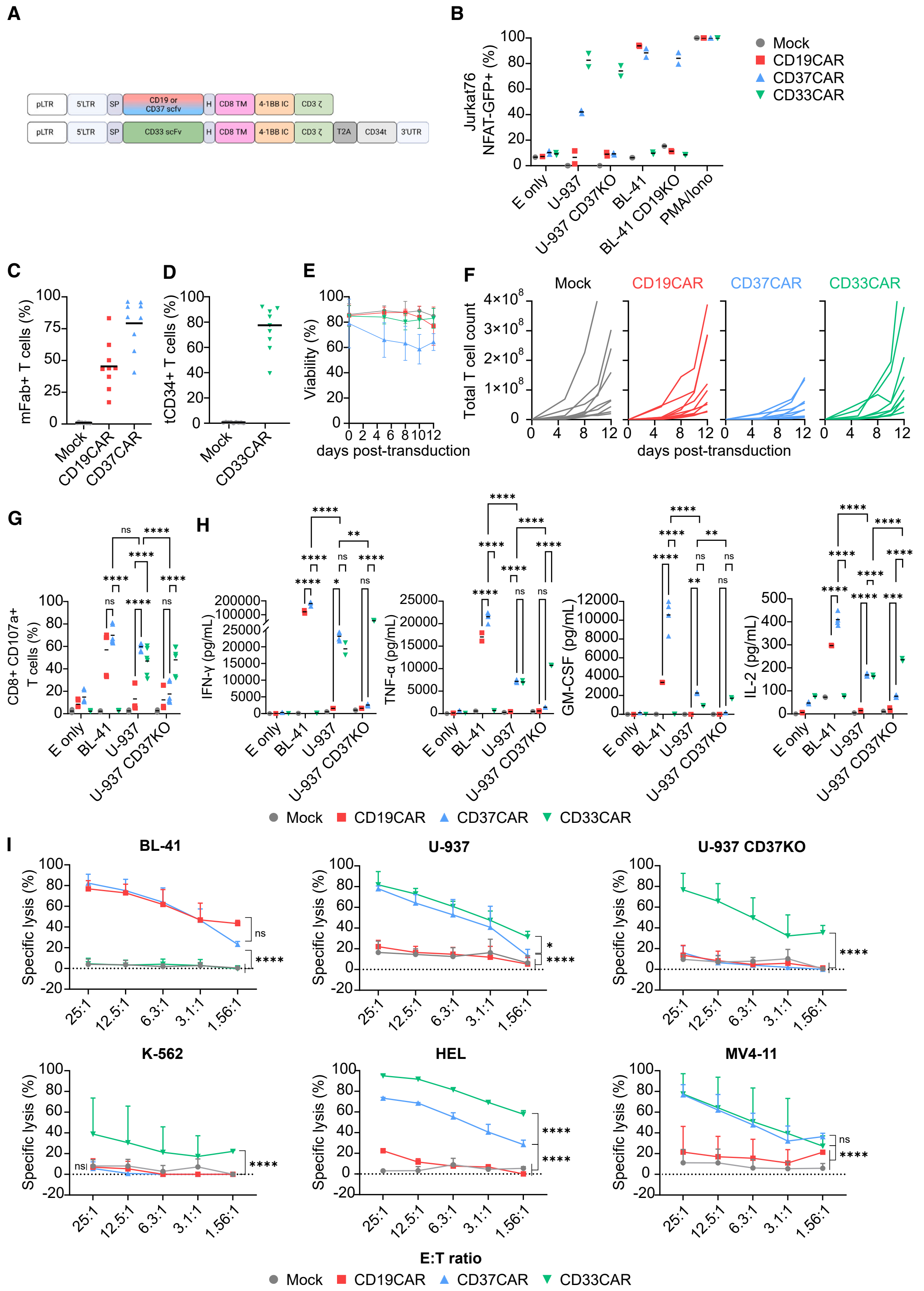

(legend on next page)

expansion of the T cells (Figure 2F). Nonetheless, CAR T cell specificity and functionality were evaluated against target cell lines. Co-cultured CAR T cells displayed a specific CD107a degranulation marker upon target cell encounter. Importantly, CD37CAR reacted against CD37^{high} BL-41 and CD37^{low} U-937 but not against the U-937^{CD37KO}. The controls, CD19CAR, and CD33CAR T cells were as potent as CD37CAR T cells against relevant targets, and all constructs activated both CD8 and CD4 T cell populations (Figures 2G and S8E). Activated CAR T cells produced inflammatory cytokines such as interferon (IFN)-γ, tumor necrosis factor (TNF)-α IL-2, and granulocyte-macrophage colony-stimulating factor (GM-CSF) following the same pattern of antigen specificity (Figures 2H and S9). Interestingly, some cytokines (G-CSF, IL-12, IL-15, IL-2, and IL-17) seemed to be produced by CD37CAR T cells in the presence of U-937^{CD37KO} or without target cells (E only), which probably reflects the tonicity of the construct (Figure S9). Finally, CAR T cell killing capacity was evaluated using bioluminescence (BLI)-based killing assays against different AML cell lines (Figure 2I, and see Figure S8C for target expression). Together, these assays demonstrate the potency and specificity of CD37CAR T cells against AML.

CD37CAR T cells efficiently kill AML cells *ex vivo* and spare normal HSCs

We next tested the ability of CD37CAR T cells to kill primary AML samples. AML bone marrow mononuclear cells (BMMCs) were co-cultured with CAR T cells for 24 h. We observed that CD37^{-} and CD33CAR T cells, but not CD19CAR T cells, specifically killed AML blasts (Figures 3A and 3B). Of note, CD33 and CD37 expression was comparable in the challenged blast population (Figure 3C). We also evaluated the cytokines secreted in the co-culture supernatants. Since the BM might contain B cells, we cannot exclude that they contributed to the stimulation of CD19^{-} and CD37CAR T cells. Likewise, CD33 CAR T cells could be stimulated by myeloid cells (Figure 3D). Although less potent than CD33CAR, CD37CAR T cells secreted cytokines corresponding to a T helper 1 (Th1)-like profile with IFN-γ, TNF-α, and IL-2. Taken together, we showed that CD37CAR T cells depleted primary patient AML cells *ex vivo* and produced inflammatory cytokines.

Next, we evaluated CD37CAR toxicity toward terminally differentiated blood cell populations and HSPCs.[12,13] Different autologous CAR T cells were generated and co-cultured with peripheral blood mononuclear cells (PBMCs) for 24 h or BMMCs for 6 h (Figures 3E–3G). BMMCs were further cultivated for 10 days in a hematopoietic colony-forming unit (CFU) assay. CD19^{-} and CD37CAR T cells depleted B cells (Figure 3F). In this assay, the effect on the monocyte population was difficult to capture (not shown); we thus run a similar co-culture assay where a larger number of monocytes was isolated and co-cultured with CAR T cells (Figure 3G). Here, CD33CAR T cells clearly reduced the monocyte population, whereas CD37CAR T cells had little or no effect and were significantly less toxic than CD33CAR T cells. We next evaluated the toxicity of the different CAR T against normal hematopoiesis and noticed that CD33CAR T cells displayed slight toxicity toward progenitors of erythroid origin ($p < 0.05$ vs. CD19CAR) while being highly toxic toward myeloid progenitors (Figure 3H; $p < 0.001$ vs. both CD19^{-} and CD37CAR). In comparison, CD37CAR T cells did not react toward either erythroid or myeloid progenitors (Figure 3H, not significant). We concluded that CD37CAR T cells are less myelotoxic than CD33CAR T cells.

CD37CAR T cells control different AML models *in vivo*

To study the efficacy of the CD37 CAR *in vivo*, we injected intravenously (i.v.) a high-dose of CAR T cells (1×10^7) into NOD xenograft gamma (NXG) mice that were challenged with U-937 cells expressing GFP-Luciferase^{+} (GFP-Luc^{+}) (Figure 4A). While mock and CD19CAR T cells failed to control tumor burden, CD37^{-} and CD33CAR T cells showed anti-AML activity with prolonged survival (Figures 4B–4D, median survival 19–21 days versus 12 in control; $p < 0.001$). The sensitivity of CD37CAR T cells to antigen density was tested using a CD37$^{very\ low}$ AML model, MOLM-13 (Figures S2C and S5C), *in vivo* (Figure 4E). Importantly, we observed with this aggressive model that CD37CAR T cells were still able to slow tumor progression (Figures 4E–4H; $p < 0.01$). In addition, in these two models, we observed that the CARs did not trigger any cytokine release syndrome based on the absence of weight loss in the animals (Figures S10A and S10B). Thus, CD37CAR is reactive against AML cell lines with a variable range of CD37 antigen density with no major side effects.

Our first attempt to evaluate CD37CAR T cells in a PDX model revealed that CD37CAR T cell fitness was probably not sufficient to control tumor growth in more complex models (Figures S11A–S11E). As previously shown, CD37CAR impeded T cell growth

Figure 2. CD37CAR T cells against AML

(A) Design of retrovirus vectors encoding second-generation CARs comprising a murine anti-human single-chain variable fragment (scFv), the CD8a hinge and transmembrane domains, the cytoplasmic domain of 4-1BB costimulatory molecule, and the CD3ζ subunit of the TCR. Sequences are provided in Figure S6.
(B) Percentage of activation of J76$^{NFAT-GFP}$ cells transduced with either mock, CD19^{-}, CD37^{-}, or CD33CAR and co-cultured for 24 h with the indicated cell lines or left alone (E only). E:T = 1:2 ($n = 2$ independent experiments, mean).
(C–F) Percentage of CAR expression (C) and (D), viability (E), and expansion in total T cell count (F) of T cell donors bearing the CAR constructs ($n = 9$) for 12 days post transduction. The CAR expression was detected using an anti-murine fragment antigen-binding (Fab) antibody for CD19^{-} and CD37CAR and an anti-CD34 mAb for CD33CAR, at day 4 post-transduction. The black bar represents the mean.
(G) Percentage of CD8^{+} CD107a^{+} T cells upon 6 h of co-culture with the indicated cell lines or left alone (E only). E:T = 1:2 ($n = 3$ donors in duplicates, mean). One-way ANOVA followed by Dunnett's multiple comparison tests is displayed, ****$p < 0.0001$, ns = not significant.
(H) Secretion (pg/mL) of IFN-γ, TNF-α, IL-2, and GM-CSF in the supernatant of T cell co-culture with the indicated cell lines or left alone (E only) after 24 h. E:T = 1:2 ($n = 2$ donors except CD37CAR $n = 4$, mean), **$p < 0.01$, ***$p < 0.001$, ****$p < 0.0001$, ns = not significant.
(I) Specific cytotoxicity of T cells incubated for 4 h with BL-41; 6 h with U-937, U-937 CD37KO, K-562, and HEL; or 7 h with MV4-11. Different E:T ratios ($n = 4$ donors except HEL = 2, mean ± SD). Two-way ANOVA followed by Tukey's (G) and (H) or Dunnett's (I) multiple comparisons tests. Comparisons versus CD37CAR are displayed, ****$p < 0.0001$, ns = not significant.

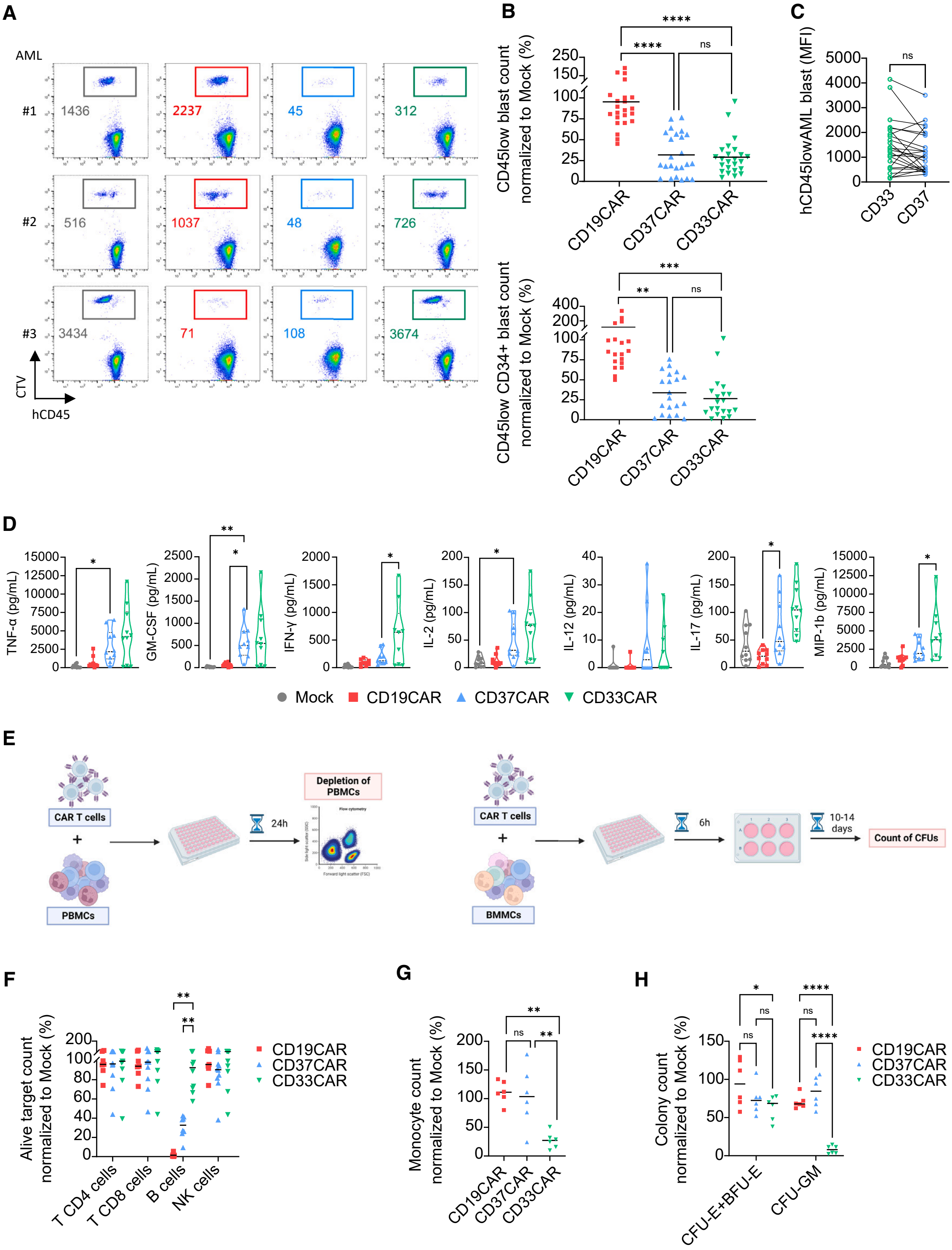

Figure 3. CD37CAR is as efficient but safer than CD33CAR

(A) Flow cytometry-based, depletion killing assay of two AML(#1 and #2) and one B-ALL (#3) patients' BMMCs labeled with CTV and co-cultured with T cells for 24 h at E:T = 5:1. Remining live cells are gated using color code corresponding to the construct expressed by T cells, mock = black, CD19CAR = red, CD37CAR = blue, and CD33CAR = green. Numbers are event count.

(B) Same as in (A), counts of live AML patients' $CD45_{low}$ blasts ($n = 24$) and $CD45_{low}$ $CD34^{+}$ blasts ($n = 20$) normalized to mock (%) after 24 h of co-culture. One-way ANOVA followed by Tukey's multiple comparisons tests, bars are mean, $^{**}p < 0.01$, $^{***}p < 0.001$, $^{****}p < 0.0001$, ns = not significant.

(legend continued on next page)

(Figure 2F). It was recently reported that the kinase inhibitor dasatinib could improve CAR T cell manufacturing,[46,47] which we also observed with CD37CAR T cells (Figures S12A and S12B) as well as the killing of CD37 and CD33CAR T cells (Figure S12C). Remarkably, dasatinib seemed to dampen CD37CAR tonicity as reflected by the decrease of $CD107^+$ $CD8^+$ CD37CAR T cells at steady state (Figure S12D). Importantly, dasatinib did not affect mock or CD33CAR T cell expansion and phenotype (Figure S12). To evaluate the efficacy of CD37CAR T cells in a more relevant pre-clinical model, we engrafted mice with GFP-Luc^+ AML patient-derived cells. As shown in Figure 5A, the AML patient-derived cells expressed CD33 and CD37 antigen. In comparison to control groups, CD37CAR T cells delayed AML growth (Figures 5B, 5C, and 5E) and extended survival of the animals (Figure 5D; $p < 0.001$). Strikingly, the dasatinib-expanded CD37CAR T cells outperformed dasatinib-expanded CD33CAR T cells and demonstrated a significantly improved median survival of 55 vs. 74 days (Figure 5D). Interestingly, the pattern of disease recurrence was different between CD37CAR and CD33CAR; in contrast to CD33CAR, the recurrence of cancer cells in mice treated with CD37CAR did not originate from expected sites such as spine or femur BM (Figures 5F and 5G, green circle) of the mice, but from skull or liver localized cells (Figure 5H, green circle). In mice treated with CD33CAR, control of disease progression was lost on day 14 post CAR T injection (Figure 5E), and disease relapse could be detected in the femur (Figures 5F, G and H, red circle) and in the spinal cord (Figure 5H, red circle). Notably, for both $CD33^-$- and CD37CAR T cell-treated mice, the recurrence of cancer cells in the PDX model was not due to antigen loss (Figure S13). These data suggest that CD37CAR has superior pre-clinical efficacy than the current anti-AML CD33CAR. Importantly, no weight loss in the animals was observed, suggesting that neither CD33 nor CD37CAR triggered cytokine release syndrome (Figures S10C and S10D).

To follow the differentiation of the CAR T cells *in vivo*, we undertook a comprehensive characterization of the CAR T cell population during the treatment. T cell populations were metaclustered by using two different levels of classifications (level 1 and level 2) based on the selection of antigens described in Table S4. As shown in Figures 5I and 5J, we compared the T cell phenotype of the different CAR T cell groups (i.e., mock, CD33, and CD37) before injection. The distribution of the CD4, CD8, double-negative (DN), and double-positive (DP) T cell populations was similar between the CD33 and CD37CAR T cells (Figure 5I). Interestingly, the distribution was slightly different for the mock group. This could reflect an effect of the CD33 and CD37CAR-T construct expression on the biology of the T cells during the activation and expansion phase of the CAR T product preparation. Using a larger panel of markers (level 2) to identify more in depth the T cell populations, we confirmed that the $CD33^-$ and CD37CAR-T products were very similar, in terms of T cell subtype population distribution, at the time of injection.

As shown in Figures 5K and 5L, we explored the differentiation phenotype of T cells during CAR T cell therapy. To align with prior studies on T cell differentiation following infection,[48] mice were sacrificed on day 6. We observed distinct distributions of the CAR T cell phenotype among the groups (Figures 5K–5L). Notably, there was an increased proportion of CD8 CM T cells in the BM of CD37CAR T-treated mice compared to CD33CAR-T cells and mock-treated mice. Conversely, the spleen of CD33 CAR T-treated mice exhibited a higher proportion of CD8 CM T cells compared to the one of CD37 CAR T-treated group. Overall, our observations highlight CAR T construct and organ dependencies in T cell phenotype and differentiation. Notably, in our experimental setup, the absence of disease recurrence in the BM of CD37 CAR T-treated mice, as opposed to CD33 CAR T-treated mice, may be attributed to the presence of more CD8 CM T cells in their BM.

DISCUSSION

In the present study, we validated the utilization of CD37 as a target for a safe CAR T cell therapy approach to treat AML. CD37 was detected by flow and mass cytometry on the surface of different AML cell lines and primary samples. This was supported by RNA-seq analysis showing that, although *CD37* mRNA was more broadly detected, it was enriched in AML samples. Because the HH1 anti-CD37 antibody efficiently detected CD37 on AML samples, we tested the efficacy of HH1-based CD37CAR in different pre-clinical experiments. We compared it to the clinically tested CD33CAR and observed a similar anti-AML activity but, unlike CD33CAR, without toxicity against healthy tissues.

Antibody- and CAR-based therapies rely on restricted surface expression of their target. CD37 previously appeared as an ideal therapeutic target: it is a lineage marker of mature B cells also highly expressed in B cell malignancies, yet its presence was

(C) Geometric median fluorescent intensity of CD33 and CD37 staining on AML patients ($n = 24$). Paired t test was used for statistical analysis, ns = not significant.
(D) Cytokine secretion (pg/mL) of TNF-α, IFN-γ, GM-CSF, G-CSF, IL-2, IL-12, IL-15, IL-17, and MIP-1b in the supernatant of (A) and (B) after 24 h of co-culture. E:T = 1:2 ($n = 2$ T cell donors co-cultured with five AML samples each; mean). One-way ANOVA followed by Dunnett's multiple comparisons tests. Only significant comparisons to CD37CAR are displayed, $^*p < 0.05$, $^{**} = p < 0.01$.
(E) Evaluation of CAR T cell toxicity toward healthy blood and hematopoiesis. Healthy donors' PBMCs (labeled CTV) (right) and BMMCs (containing $CD34^+$) (left) were co-incubated with autologous T cells. After 24 h of co-culture, PBMCs were then analyzed by flow cytometry for depletion killing. After 6 h of co-culture, BMMCs were further cultured for 10 days in a colony-forming unit (CFU) assay.
(F) Counts of $CD45^+$ $CD3^+$ $CD4^+$ T cells alive, $CD45^+$ $CD3^+$ $CD8^+$ T cells, $CD45^+$ $CD19^+$ B cells, $CD45^+$ $CD3^-$ $CD19^-$ $CD56^+$ natural killer (NK) cells, and $CD45^+$ $CD14^+$ monocytes normalized to mock (%) after 24 h of co-culture ($n = 7$ donors for CD19CAR and $n = 10$ donors for $CD37^-$and CD33CAR; mean). Bars are mean, two-way ANOVA followed by Tukey's multiple comparisons tests. Only significant comparisons are displayed, $^{**}p < 0.01$.
(G) Counts of $CD45^+$ $CD11b^+$ monocytes alive normalized to mock (%) after 24 h of co-culture with CD19CAR, CD37CAR, and CD33CAR ($n = 6$ donors). Bars represent means, one-way ANOVA followed by Tukey's multiple comparisons tests, $^{**}p < 0.01$, ns = not significant.
(H) CFU counts of erythroid colonies (CFU-erythroid and burst-forming unit-erythroid) and myeloid colonies (CFU-granulocyte macrophage) normalized to mock (%) ($n = 2$ donors, triplicates, mean). Two-way ANOVA followed by Tukey's multiple comparisons tests, $^*p < 0.05$, $^{****}p < 0.0001$, ns = not significant.

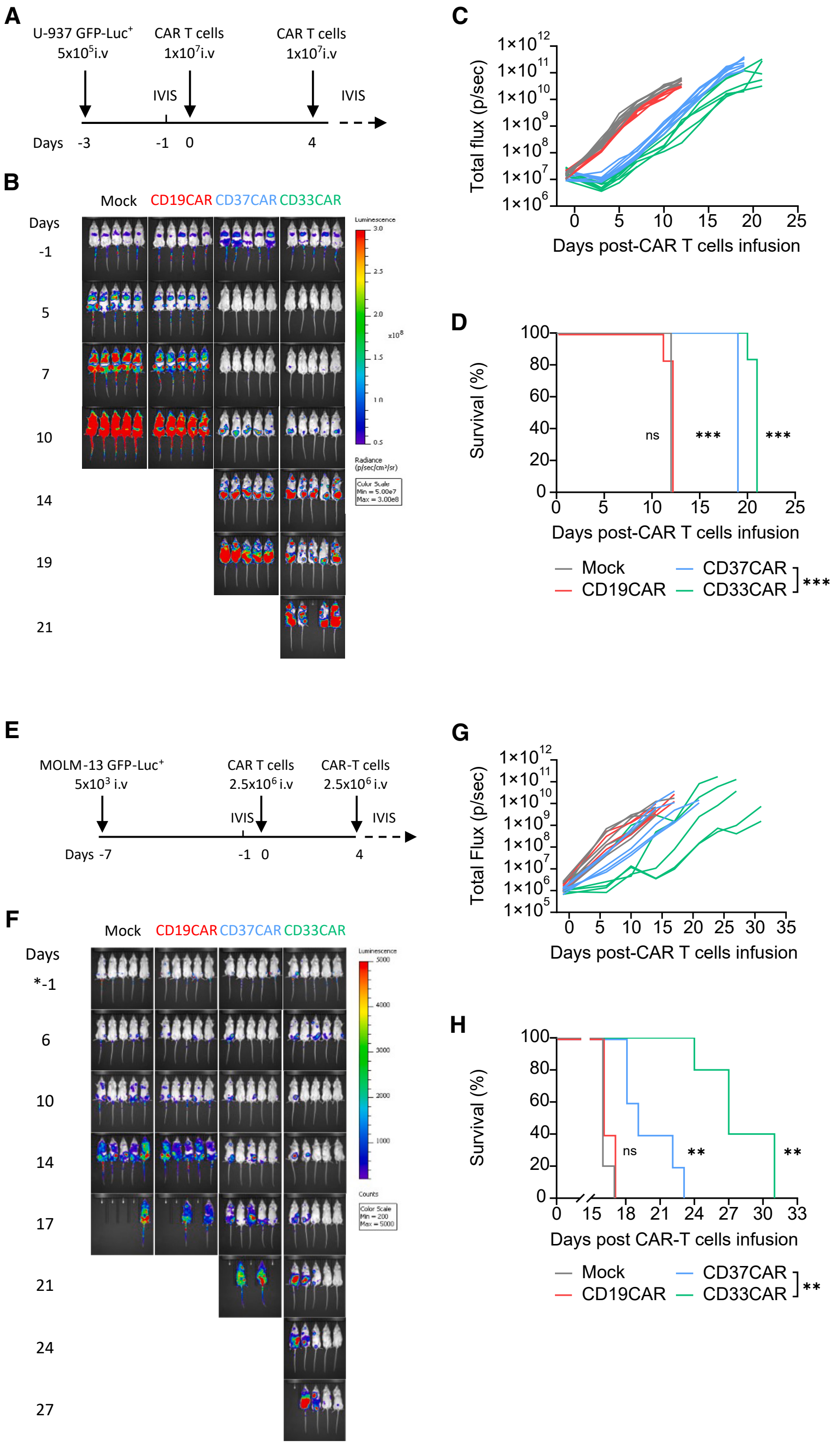

Figure 4. CD37CAR T cells have potent anti-AML activity *in vivo*

(A) Schematic of the U-937-based *in vivo* experimental design. Three days before T cell injection, 5×10^5 U-937 GFP-Luc$^+$ cells were inoculated intravenously (i.v.) in NXG mice. *In vivo* imaging system (IVIS) was performed 1 day before T cell injection to confirm tumor establishment and randomize the mice. On day 0 and day 4, 1×10^7 mock, CD19$^-$, CD37$^-$, or CD33CAR T cells were injected i.v. The percentage of CAR-expressing population was adjusted between the groups to 50% using mock cells. Tumor growth was tracked two times a week using IVIS.

(B) Representative bioluminescence images.

(C) Bioluminescence kinetics of U-937 GFP-Luc$^+$ cells growth in NXG mice treated with CAR T cells (n = 6 mice per group).

(D) Kaplan-Meier survival curves of NXG mice bearing U-937 GFP-Luc$^+$ cells and treated with CAR T cells (n = 6 mice per group). Comparisons of survival curves were determined by log rank test.

(E) Schematic of the MOLM-13-based *in vivo* experimental design. Seven days before T cell injection, 5×10^3 MOLM-13 GFP-Luc$^+$ cells were inoculated i.v. in NXG mice. IVIS was performed 1 day before T cell injection to confirm tumor establishment and randomize the mice. On day 0 and day 4, 2.5×10^6 mock, CD19$^-$, CD37$^-$, or CD33CAR T cells were injected i.v. The percentage of the CAR-expressing population was adjusted between the groups to 40% using mock cells. Tumor growth was tracked two times a week using IVIS.

(F) Representative bioluminescence images.

(G) Bioluminescence kinetics of MOLM-13 GFP-Luc$^+$ cells growth in NXG mice treated with CAR T cells (n = 5 mice per group).

(H) Kaplan-Meier survival curves of NXG mice bearing MOLM-13 GFP-Luc$^+$ cells and treated with CAR T cells (n = 5 mice per group). Comparisons of survival curves were determined by log rank test, ***$p < 0.001$, ns = not significant.

approach described for the treatment of B cell leukemia, B cell lymphoma,[26,29] and T cell lymphoma.[31] We established that the *CD37* mRNA was overexpressed in a wide range of AML patients' samples, especially in the adverse risk AML group, such as t(11q23)/MLL AML, indicating that this AML population could potentially benefit from a CAR therapy targeting CD37. Because gene expression profile analysis may not fully predict protein expression, we investigated the presence of CD37 on AML cell lines by flow cytometry and on 59 AML patient samples by

detected at a much lower level in monocytes[22,23] and activated T cells.[20] Still, this did not seem to lead to any toxic effects since therapeutic anti-CD37 antibodies were reported to be safe.[49,50] Our group and others have recently developed a CD37-targeting CAR T cell-based therapy[20,31,51] mirroring the CD19CAR T cell

mass cytometry. The CD37 protein expression on the AML cells significantly correlated with the ELN 2017 patient risk stratification. A recent study[52] has linked CD37 protein to fatty acid metabolism to aggressive B cell lymphoma. It is tempting to speculate that AML cells, which are dependent on long-chain fatty acid

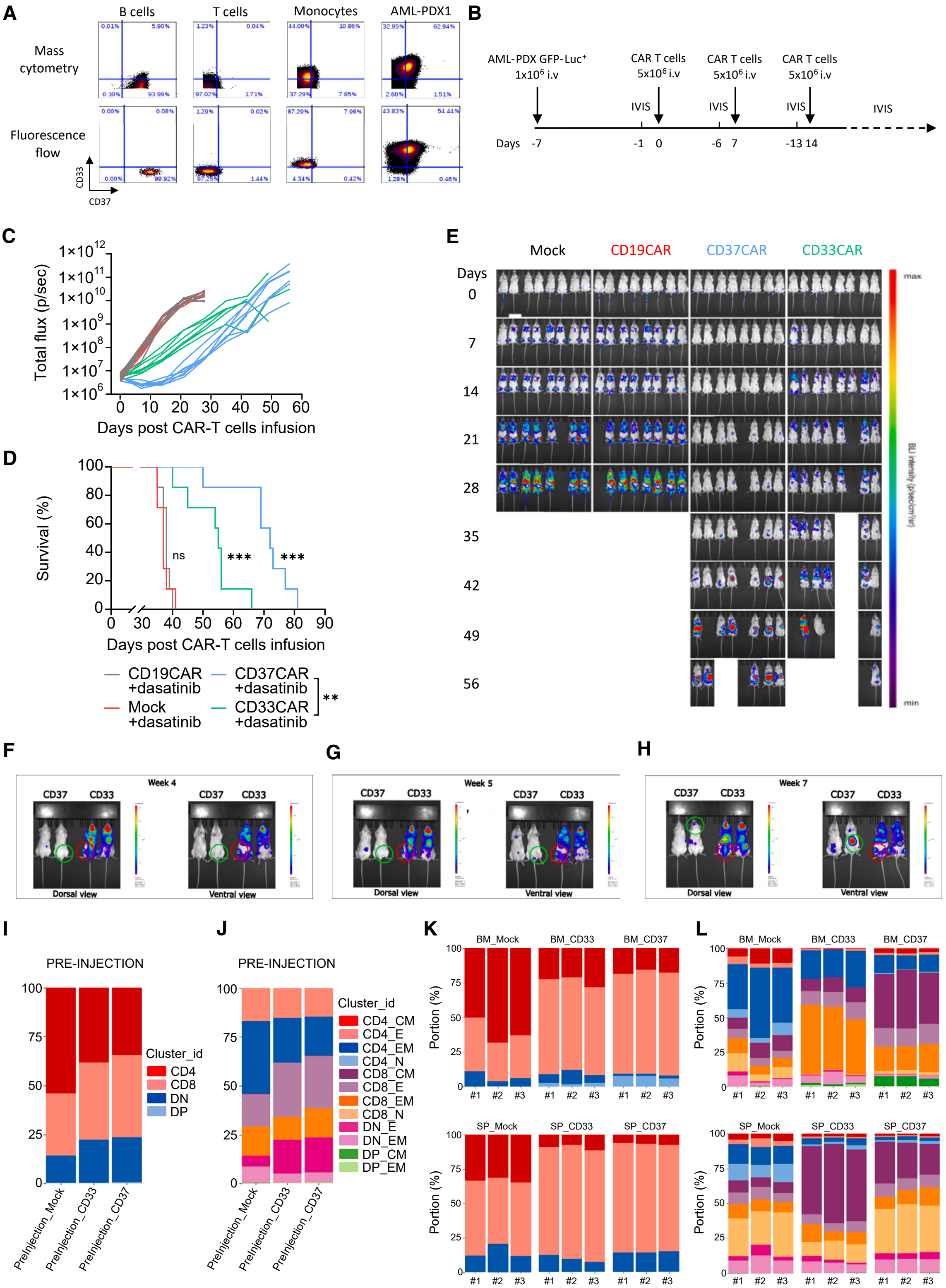

Figure 5. CD37CAR T cells control AML-PDX model

(A) Detection of CD37 and CD33 in AML-PDX1 cells and healthy donor cells by mass cytometry and flow cytometry.

(B) Schematic representation of the PDX *in vivo* experimental design. Seven days before the first T cell injection, 1 × 10^6 AML-PDX GFP-Luc$^+$ cells (F2) were inoculated i.v. in NOD scid gamma (NSG) mice. IVIS was performed 1 day before T cell injection to confirm tumor establishment and randomize the mice, and 5 ×

(legend continued on next page)

metabolism for their survival,[53] might also require CD37 to increase their fitness. Thus, the increased presence of CD37 would follow the resistance to standard treatments by enhancing their metabolic efficiency. Our results are in accordance with previous findings, using immunohistochemistry and flow cytometry-based approaches, showing that AML cells overexpressed CD37 at the protein level compared to healthy $CD34^+$ cells and that the density on AML LSCs is equal to, if not higher than, that of AML myeloblasts.[24] We sought to validate our target by rationalizing its expression in comparison to the pan-myeloid CD33 marker and the CD123 stem cell marker currently evaluated in CAR clinical trials for AML.[7] We observed an equivalent frequency of expression of around 80% of the primary blasts expressing the three proteins regardless of the prognosis factor and CD34 expression. This provides evidence that CD37 is expressed at the cell surface and represents a rational biomarker in term of expression, equivalent to CD33 and CD123, for the characterization of AML cells. In contrast to CD37, the expression of CD33 did not correlate with the AML subtypes or ELN risk stratification. Hence, unlike CD33 and CD123 showing broader expression outside the hematopoietic system,[54] CD37 distribution seems to be restricted to terminally differentiated blood cells,[21,22] making CD37 an attractive target for cell therapy in AML.

The relapsed and refractory forms of AML are in part presumably due to the presence of a persisting population of LSCs that is resistant to chemotherapy and replenishes the pool of AML blasts.[9] Therefore, assessing the presence of CD37 in these cells is of great interest to confirm that a CD37-based CAR T therapy can eradicate the disease at its roots. Previous studies have confirmed the expression of CD33 and CD123 in R/R AML, including LSCs.[54] Here, we described that CD37 expression can be detected on 70%–80% of both GMP- and LMPP-like LSC patient blasts. The observation that these two LSC populations coexist in 80% of patients[9] and that the primitive LMPP-like LSCs are found enriched in recurrent AML[55] warrants clinical investigation of CD37 CAR T cells in AML treatment.

Remarkably, the detection of CD37 was not straightforward and, by using different commercial antibodies, we unexpectedly noticed discrepancies in staining of CD37 in AML that were not apparent in B cells. We anticipated that different splice variants could be involved in this cell-type recognition, but we did not identify any differences. AML patients have not been reported to carry CD37 mutations and the patient samples used in our study did not have detectable CD37 mutations. However, despite the fact that AML could be characterized as a disease with low tumor mutational burden, more extensive analysis could be undertaken to exclude the presence of CD37 mutations in AML patients. The sensitive of the anti-CD37 antibody could be affected by the cell-surface antigen density or post-translational modifications such as glycosylation.[17,23] In fact, aberrant glycosylation is considered a hallmark of cancer and it has been exploited to develop glycoantigen-specific antibodies and CARs.[56] However, using an enzyme removing sialic acid groups, a common modification of glycosylated proteins in cancer, did not alter HH1 antibody and CAR recognition of AML cells. Accurately determining the HH1 dependency on glycosylation will require deeper structural analysis. Regardless, the HH1-based CAR construct conserved its ability to recognize CD37 in the context of AML.

Although CD37 levels on AML are low compared to B cell malignancies, we clearly demonstrate that AML can efficiently be targeted using HH1 antibody-derived CD37CAR. We further show that this CD37CAR was as efficient as, or superior to, CD33CAR in several models. The CD37 target carries the advantage of low expression in healthy tissues except in mature B cells. Moreover, it is important to note that the CD37CAR T cells will only eliminate terminally differentiated B cells and leave a naive population. Thus, in a clinical setting, B cell aplasia might be expected but will be manageable.[57] None of the AML CAR antigens investigated so far are exclusively expressed on AML cells and often share expression with healthy HSPCs or other non-hematopoietic tissues.[7,54]For example, although successful in pre-clinical studies, $CD33^-$ and CD123CAR T cells cause long-term myeloablation, which can lead to serious toxicity issues.[11,12] A few other CARs targeting early developmental markers have been shown to spare myeloid cells.[58,59] However, one of these targets, CD70, is expressed on activated lymphocytes and subsets of dendritic cells, and another, Siglec-6, is expressed on mast cells and basophils.[60]On the other hand, CD37CAR T cells have shown no toxicity and no inflammatory cytokine production against differentiated cell lineages other than B cells.[20,31,32] We also observed that CD37CAR T cells showed superior ability to control disease recurrence in the BM in comparison to CD33CAR T cells. Interestingly, our phenotypic characterization demonstrated CAR

10^6 mock, $CD19^-$, $CD37^-$, or CD33CAR T cells expanded with dasatinib were injected i.v. on day 0, 7, and 14. The percentage of CAR-expressing population was adjusted between the groups to 50% using mock cells. Tumor growth was tracked weekly using IVIS for 6 weeks after T cell injection.

(C) Bioluminescence kinetics of the AML-PDX GFP-Luc^+ cells growth in NSG mice treated with CAR T cells (n = 7 mice per group).

(D) Kaplan-Meier survival curves of NSG mice bearing AML-PDX GFP-Luc^+ cells and treated with CAR T cells (n = 7 mice per group). Comparisons of survival curves were determined by log rank test, ***$p < 0.001$, ns = not significant.

(E) Representative bioluminescence images.

(F) Representative bioluminescence images of close-up BM area of mice treated with CD33CAR or CD37CAR. Dorsal and ventral view comparing the bioluminescence at week 4. The green circle (CD37CAR-treated mice) and the red circle (CD33CAR-treated mice) emphasize the re-growth of AML cancer cells in the mice.

(G) Same as in (F) at week 5.

(H) Same as in (F) at week 7.

(I) MC of group CAR T cell at pre-injection using level 1 depth characterization. Pool of three mice per group.

(J) MC of group CAR T cell at pre-injection using level 2 depth characterization. Pool of three mice per group.

(K) MC of CAR T cells from three mice per group (#1, #2, #3) at day 6 after CAR T injection, grouped by anatomical site using level 1 depth characterization.

(L) MC of CAR T cells from three mice per group (#1, #2, #3) at day 6 after CAR T injection, grouped by anatomical site using level 2 depth characterization.

T cell differentiation *in vivo* to be organ dependent. Indeed, we showed that the CD33 and CD37CAR T cell products were nearly identical at the level of T cell subpopulation distribution before injection. However, after 6 days, the distribution of the different T cell populations was clearly distinct between the CD33⁻ and CD37CAR T cells. Interestingly, we could observe a higher proportion of CD8 CM T cells in the BM of animals treated with CD37CAR T cells when compared to mice treated with the CD33CAR and mock T cells. In addition, we observed that disease recurrence never occurred in the BM of the mice treated with CD37CAR T cells, which contrasts with the other groups. This might be linked to the increased presence of CD8 CM T cells in the BM of the CD37CAR T cell group and the ability of these cells to drive a more potent T cell differentiation that will result in improved control of the AML. Previous studies investigating T cell differentiation after infection have connected it to antigen recognition, directly shaped by the functional avidity of the T cell receptor (TCR) for its target.[48,61–63] It is tempting to speculate that the differentiation spectrum of the CAR T cells and the performance of the different CAR T cell products is linked to its functional avidity, which might differ between CD37CAR and CD33CAR.[48] Even if our experimental setup cannot fully address this question, our study detected a striking modification in the differentiation profile of the CAR groups *in vivo*. Nonetheless, our investigation has highlighted that CAR T cell differentiation *in situ* depends on the single-chain variable fragment (scFv) and/or the antigen density on the targeted organ.

Interestingly, Okuno et al.[20] reported that a short and transient expression of CD37 during T cell activation can affect T cell expansion. The authors suggested that the lower efficacy in T cell expansion was reminiscent of T cell fratricide killing. However, fratricide was never experimentally demonstrated for CD37CAR T cells and we rather attribute this lack of expansion to toxicity caused by the strong tonic signaling (Forcados, Wälchli, et al., unpublished data). In line with this idea, chronic toxic signaling was also reported with CAR targeting GRP78, which would be induced by the encounter of the CAR molecule and the target within the T cell. Intriguingly, the authors resolved the tonic toxicity issue by inhibiting endogenous CAR activation during CAR T cell manufacturing using the kinase inhibitor dasatinib.[46] The efficacy of dasatinib at preventing tonic signaling was further confirmed in a study using a CAR targeting CD7.[64] Our results also show that the presence of dasatinib during CD37CAR T cell manufacturing can restore T cell expansion without affecting their functional capacities.

In summary, CD37 is an attractive CAR target. It is widely expressed at the surface of primary AML cells, including LSCs. Moreover, we uncover that the expression of CD37 in myeloid cells is associated with ELN 2017 risk stratification and thus is related to adverse outcome and poor survival. In addition, CD37 biodistribution predicts lower on-target toxicity over the other AML CAR targets. Our *in vitro* and *in vivo* studies highlight the potent anti-AML activity of HH1-based CD37CAR, which is also able to spare normal myeloid cells and stem cells. This is an important feature and, in several cases, the targets are genetically manipulated by disruption[65] or base correction[66] to circumvent myeloid and stem cell toxicity. Thus, our findings strongly warrant clinical investigation of CD37CAR to treat AML patients.

Limitations of the study

Although we present *in vivo* data from different AML cell lines and PDX, experiments with lower number of CAR T cell injections would provide additional insight on the robustness of CD37CAR T cells to treat AML in more physiological conditions. Furthermore, studying long-term T cell persistence in animals could support prediction of the clinical CAR product efficacy, but human T cells in these model systems often cause xenogeneic graft-versus-host disease (GvHD), hampering long-term monitoring. We focused our study on the presented 4-1BB-containing CD37CAR design; however, alternative signaling tails could be compared to identify the most efficient anti-AML CD37CAR.

STAR★METHODS

Detailed methods are provided in the online version of this paper and include the following:

- KEY RESOURCES TABLE
- RESOURCE AVAILABILITY
  - Lead contact
  - Materials availability
  - Data and code availability
- EXPERIMENTAL MODEL AND STUDY PARTICIPANT DETAILS
  - Cell lines
  - Human samples
  - Mouse xenograft studies
- METHOD DETAILS
  - Gene expression analysis
  - Single cell transcriptomic analysis
  - Mass cytometry
  - DNA constructs
  - Immunophenotyping by flow cytometry
  - Retroviral transduction and expansion of human T cells
  - Isoform detection assay
  - Reporter assay
  - Generation of CD37 knock-out U-937cells
  - Degranulation analysis
  - Cytokine quantification
  - Bioluminescence (BLI)-based cytotoxicity assay
  - Cytometry-based cytotoxicity assay
  - Colony-forming unit (CFU) assay
- QUANTIFICATION AND STATISTICAL ANALYSIS

SUPPLEMENTAL INFORMATION

Supplemental information can be found online at https://doi.org/10.1016/j.xcrm.2024.101572.

ACKNOWLEDGMENTS

We are grateful to MSc. Lizet Baken (former OUS) for performing the preliminary experiments of this project, to Dr. Pilar Ayuda Duran (OUS) for her help with the selection of AML samples from the Biobank to MSc, and to Sepehr Teimouri (OUS) and Dr. Marit Renée Myhre (former OUS) for their support with the animal studies. We also thank Drs. Mihaela Popa (UiB) and Mireia Safont (UiB) for the PDX pre-clinical experiments and related flow cytometry. This study was partially supported by the National Center for Research and Development (Poland) within the POLNOR program ALTERCAR (0056/2019), the Norwegian Health Authority South-East (2022009, 2024080), The Norwegian

CellPress
OPEN ACCESS

Childhood Cancer Society (PERCAP, 230004), and the Research Council of Norway (NFR, 337468) to S.W. and from the NFR KSP-2021 CellFit project (326811) to E.M.I. N.K. and B.C. are postdoctoral fellows of the Norwegian Cancer Society (208012), and B.C. is also supported by the Scientia Fellow II—Marie Skłodowska-Curie Actions program (801133). S.J. is a PhD student supported by era-net EURONANOMED-3 NAN-4-TUM (310531). F.K. is a postdoctoral fellow supported by the NFR KSP-2021 CellFit project (326811), and P.W. was a former postdoctoral fellow supported by an NFR BIOTEK grant (284983). J.M.E. received funding from the Norwegian Cancer Society (182524 and 208012), the Norwegian Health Authority South-East (2017064, 2017072, 2018012, 2019096), and the Research Council of Norway (261936, 301268, 262652). E.M.C. and P.G. were supported by a grant from the Research Council of Norway (326300) and the Norwegian Cancer Society (223171). T.H.D. and M.E.G. are recipients of a University of Bergen PhD fellowship. S.K. is supported by the Marie Sklodowska-Curie Program Training Network for Optimizing Adoptive T Cell Therapy of Cancer funded by the H2020 Program of the European Union (grant 955575); by the Hector Foundation; by the International Doctoral Program i-Target, Immunotargeting of Cancer funded by the Elite Network of Bavaria; by the Else Kröner-Fresenius-Stiftung (2021_EKFK_01); by the German Cancer Aid (AvantCAR.de); by the Ernst-Jung-Stiftung; by the Bundesministerium für Bildung und Forschung (CONTRACT); by the Go-Bio Initiative; by the m4 award of the Bavarian Ministry for Economical Affairs; by the Bayerische Forschungsstiftung (Baycellator); by the Wilhelm Sander-Stiftung (2022.051.1); by the European Research Council grants 756017 and 101100460, Deutsche Forschungsgemeinschaft (DFG; KO5055-2-1 and 510821390); by the SFB-TRR 338/1 2021–452881907; by the Fritz Bender Foundation; and by the Deutsche José Carreras Leukämie-Stiftung. We are grateful to the European Genome-Phenome Archive (EGA) for granting us access to their database. We thank the Flow Cytometry Core Facility and the Department of Comparative Medicine at the Oslo University Hospital.

AUTHOR CONTRIBUTIONS

Conceptualization, S.W., E.M.I., and G.K.; methodology, E.M.I., B.C., S.J., F.K., P.W., T.H.D., C.W., P.G., E.M.C., and S.W.; software, F.K., P.W., M.T., C.M., and S.K.; investigation, B.C., P.G., P.W., N.U.K., T.H.D., C.W., M.E.G., S.J., F.K., S.W., J.M.E., and L.T.; resources/funding acquisition, E.M.I., S.W., E.M.C., and B.T.G.; visualization, B.C., T.H.D., F.K., S.J., P.G., and M.T.; writing – original draft, B.C. and S.W.; writing – review & editing, all authors; supervision, E.M.I., B.T.G., and S.W.

DECLARATION OF INTERESTS

The CD37CAR construct has been patented (WO2017118745A1) and E.M.I., G.K., and S.W. are listed among the inventors. S.K. has received honoraria from TCR2 Inc., Miltenyi, Novartis, BMS, and GSK. S.K. is inventor of several patents in the field of immuno-oncology. S.K. received license fees from TCR2 Inc. and Carina Biotech. S.K. received research support from TCR2 Inc., Plectonic GmbH, Tabby Therapeutics, and Arcus Bioscience for work unrelated to this manuscript. The funding agencies had no role in the conduction and management of the presented research and were not involved in the preparation of this manuscript.

Received: October 30, 2023
Revised: March 5, 2024
Accepted: April 23, 2024
Published: May 15, 2024

REFERENCES

1. Dohner, H., Weisdorf, D.J., and Bloomfield, C.D. (2015). Acute Myeloid Leukemia. N. Engl. J. Med. *373*, 1136–1152. https://doi.org/10.1056/NEJMra1406184.
2. Dohner, H., Wei, A.H., Appelbaum, F.R., Craddock, C., DiNardo, C.D., Dombret, H., Ebert, B.L., Fenaux, P., Godley, L.A., Hasserjian, R.P., et al. (2022). Diagnosis and management of AML in adults: 2022 recommendations from an international expert panel on behalf of the ELN. Blood *140*, 1345–1377. https://doi.org/10.1182/blood.2022016867.
3. Döhner, H., Estey, E., Grimwade, D., Amadori, S., Appelbaum, F.R., Büchner, T., Dombret, H., Ebert, B.L., Fenaux, P., Larson, R.A., et al. (2017). Diagnosis and management of AML in adults: 2017 ELN recommendations from an international expert panel. Blood *129*, 424–447. https://doi.org/10.1182/blood-2016-08-733196.
4. Williams, B.A., Law, A., Hunyadkurti, J., Desilets, S., Leyton, J.V., and Keating, A. (2019). Antibody Therapies for Acute Myeloid Leukemia: Unconjugated, Toxin-Conjugated, Radio-Conjugated and Multivalent Formats. J. Clin. Med. *8*, 1261. https://doi.org/10.3390/jcm8081261.
5. Boddu, P., Kantarjian, H., Garcia-Manero, G., Allison, J., Sharma, P., and Daver, N. (2018). The emerging role of immune checkpoint based approaches in AML and MDS. Leuk. Lymphoma *59*, 790–802. https://doi.org/10.1080/10428194.2017.1344905.
6. Majeti, R. (2011). Monoclonal antibody therapy directed against human acute myeloid leukemia stem cells. Oncogene *30*, 1009–1019. https://doi.org/10.1038/onc.2010.511.
7. Daver, N., Alotaibi, A.S., Bücklein, V., and Subklewe, M. (2021). T-cell-based immunotherapy of acute myeloid leukemia: current concepts and future developments. Leukemia *35*, 1843–1863. https://doi.org/10.1038/s41375-021-01253-x.
8. Estey, E.H. (2018). Acute myeloid leukemia: 2019 update on risk-stratification and management. Am. J. Hematol. *93*, 1267–1291. https://doi.org/10.1002/ajh.25214.
9. Thomas, D., and Majeti, R. (2017). Biology and relevance of human acute myeloid leukemia stem cells. Blood *129*, 1577–1585. https://doi.org/10.1182/blood-2016-10-696054.
10. Thol, F., and Ganser, A. (2020). Treatment of Relapsed Acute Myeloid Leukemia. Curr. Treat. Options Oncol. *21*, 66. https://doi.org/10.1007/s11864-020-00765-5.
11. Kenderian, S.S., Ruella, M., Shestova, O., Klichinsky, M., Aikawa, V., Morrissette, J.J.D., Scholler, J., Song, D., Porter, D.L., Carroll, M., et al. (2015). CD33-specific chimeric antigen receptor T cells exhibit potent preclinical activity against human acute myeloid leukemia. Leukemia *29*, 1637–1647. https://doi.org/10.1038/leu.2015.52.
12. Gill, S., Tasian, S.K., Ruella, M., Shestova, O., Li, Y., Porter, D.L., Carroll, M., Danet-Desnoyers, G., Scholler, J., Grupp, S.A., et al. (2014). Preclinical targeting of human acute myeloid leukemia and myeloablation using chimeric antigen receptor-modified T cells. Blood *123*, 2343–2354. https://doi.org/10.1182/blood-2013-09-529537.
13. Mardiros, A., Dos Santos, C., McDonald, T., Brown, C.E., Wang, X., Budde, L.E., Hoffman, L., Aguilar, B., Chang, W.C., Bretzlaff, W., et al. (2013). T cells expressing CD123-specific chimeric antigen receptors exhibit specific cytolytic effector functions and antitumor effects against human acute myeloid leukemia. Blood *122*, 3138–3148. https://doi.org/10.1182/blood-2012-12-474056.
14. Ritchie, D.S., Neeson, P.J., Khot, A., Peinert, S., Tai, T., Tainton, K., Chen, K., Shin, M., Wall, D.M., Hönemann, D., et al. (2013). Persistence and efficacy of second generation CAR T cell against the LeY antigen in acute myeloid leukemia. Mol. Ther. *21*, 2122–2129. https://doi.org/10.1038/mt.2013.154.
15. Orlando, E.J., Han, X., Tribouley, C., Wood, P.A., Leary, R.J., Riester, M., Levine, J.E., Qayed, M., Grupp, S.A., Boyer, M., et al. (2018). Genetic mechanisms of target antigen loss in CAR19 therapy of acute lymphoblastic leukemia. Nat. Med. *24*, 1504–1506. https://doi.org/10.1038/s41591-018-0146-z.
16. Majzner, R.G., and Mackall, C.L. (2018). Tumor Antigen Escape from CAR T-cell Therapy. Cancer Discov. *8*, 1219–1226. https://doi.org/10.1158/2159-8290.CD-18-0442.
17. Hemler, M.E. (2005). Tetraspanin functions and associated microdomains. Nat. Rev. Mol. Cell Biol. *6*, 801–811. https://doi.org/10.1038/nrm1736.

18. Lapalombella, R., Yeh, Y.Y., Wang, L., Ramanunni, A., Rafiq, S., Jha, S., Staubli, J., Lucas, D.M., Mani, R., Herman, S.E.M., et al. (2012). Tetraspanin CD37 directly mediates transduction of survival and apoptotic signals. Cancer Cell *21*, 694–708. https://doi.org/10.1016/j.ccr.2012.03.040.

19. Bobrowicz, M., Kubacz, M., Slusarczyk, A., and Winiarska, M. (2020). CD37 in B Cell Derived Tumors-More than Just a Docking Point for Monoclonal Antibodies. Int. J. Mol. Sci. *21*, 9531. https://doi.org/10.3390/ijms21249531.

20. Okuno, S., Adachi, Y., Terakura, S., Julamanee, J., Sakai, T., Umemura, K., Miyao, K., Goto, T., Murase, A., Shimada, K., et al. (2021). Spacer Length Modification Facilitates Discrimination between Normal and Neoplastic Cells and Provides Clinically Relevant CD37 CAR T Cells. J. Immunol. *206*, 2862–2874. https://doi.org/10.4049/jimmunol.2000768.

21. Schaper, F., and van Spriel, A.B. (2018). Antitumor Immunity Is Controlled by Tetraspanin Proteins. Front. Immunol. *9*, 1185. https://doi.org/10.3389/fimmu.2018.01185.

22. de Winde, C.M., Zuidscherwoude, M., Vasaturo, A., van der Schaaf, A., Figdor, C.G., and van Spriel, A.B. (2015). Multispectral imaging reveals the tissue distribution of tetraspanins in human lymphoid organs. Histochem. Cell Biol. *144*, 133–146. https://doi.org/10.1007/s00418-015-1326-2.

23. Schwartz-Albiez, R., Dörken, B., Hofmann, W., and Moldenhauer, G. (1988). The B cell-associated CD37 antigen (gp40-52). Structure and subcellular expression of an extensively glycosylated glycoprotein. J. Immunol. *140*, 905–914.

24. Pereira, D.S., Guevara, C.I., Jin, L., Mbong, N., Verlinsky, A., Hsu, S.J., Aviña, H., Karki, S., Abad, J.D., Yang, P., et al. (2015). AGS67E, an Anti-CD37 Monomethyl Auristatin E Antibody-Drug Conjugate as a Potential Therapeutic for B/T-Cell Malignancies and AML: A New Role for CD37 in AML. Mol. Cancer Therapeut. *14*, 1650–1660. https://doi.org/10.1158/1535-7163.MCT-15-0067.

25. Zou, F., Wang, X., Han, X., Rothschild, G., Zheng, S.G., Basu, U., and Sun, J. (2018). Expression and Function of Tetraspanins and Their Interacting Partners in B Cells. Front. Immunol. *9*, 1606. https://doi.org/10.3389/fimmu.2018.01606.

26. Barrena, S., Almeida, J., Yunta, M., López, A., Fernández-Mosteirín, N., Giralt, M., Romero, M., Perdiguer, L., Delgado, M., Orfao, A., and Lazo, P.A. (2005). Aberrant expression of tetraspanin molecules in B-cell chronic lymphoproliferative disorders and its correlation with normal B-cell maturation. Leukemia *19*, 1376–1383. https://doi.org/10.1038/sj.leu.2403822.

27. Yoshimura, T., Miyoshi, H., Shimono, J., Nakashima, K., Takeuchi, M., Yanagida, E., Yamada, K., Shimasaki, Y., Moritsubo, M., Furuta, T., et al. (2022). CD37 expression in follicular lymphoma. Ann. Hematol. *101*, 1067–1075. https://doi.org/10.1007/s00277-022-04785-z.

28. Xu-Monette, Z.Y., Li, L., Byrd, J.C., Jabbar, K.J., Manyam, G.C., Maria de Winde, C., van den Brand, M., Tzankov, A., Visco, C., Wang, J., et al. (2016). Assessment of CD37 B-cell antigen and cell of origin significantly improves risk prediction in diffuse large B-cell lymphoma. Blood *128*, 3083–3100. https://doi.org/10.1182/blood-2016-05-715094.

29. Bertoni, F., and Stathis, A. (2016). Staining the target: CD37 expression in lymphomas. Blood *128*, 3022–3023. https://doi.org/10.1182/blood-2016-11-748137.

30. Zhang, Q., Han, Q., Zi, J., Song, C., and Ge, Z. (2020). CD37 high expression as a potential biomarker and association with poor outcome in acute myeloid leukemia. Biosci. Rep. *40*. https://doi.org/10.1042/BSR20200008.

31. Scarfo, I., Ormhoj, M., Frigault, M.J., Castano, A.P., Lorrey, S., Bouffard, A.A., van Scoyk, A., Rodig, S.J., Shay, A.J., Aster, J.C., et al. (2018). Anti-CD37 chimeric antigen receptor T cells are active against B- and T-cell lymphomas. Blood *132*, 1495–1506. https://doi.org/10.1182/blood-2018-04-842708.

32. Koksal, H., Dillard, P., Josefsson, S.E., Maggadottir, S.M., Pollmann, S., Fane, A., Blaker, Y.N., Beiske, K., Huse, K., Kolstad, A., et al. (2019). Preclinical development of CD37CAR T-cell therapy for treatment of B-cell lymphoma. Blood Adv *3*, 1230–1243. https://doi.org/10.1182/bloodadvances.2018029678.

33. Golubovskaya, V., Zhou, H., Li, F., Valentine, M., Sun, J., Berahovich, R., Xu, S., Quintanilla, M., Ma, M.C., Sienkiewicz, J., et al. (2021). Novel CD37, Humanized CD37 and Bi-Specific Humanized CD37-CD19 CAR-T Cells Specifically Target Lymphoma. Cancers *13*, 981. https://doi.org/10.3390/cancers13050981.

34. Pagel, J.M., Spurgeon, S.E., Byrd, J.C., Awan, F.T., Flinn, I.W., Lanasa, M.C., Eisenfeld, A.J., Stromatt, S.C., and Gopal, A.K. (2015). Otlertuzumab (TRU-016), an anti-CD37 monospecific ADAPTIR therapeutic protein, for relapsed or refractory NHL patients. Br. J. Haematol. *168*, 38–45. https://doi.org/10.1111/bjh.13099.

35. Deckert, J., Park, P.U., Chicklas, S., Yi, Y., Li, M., Lai, K.C., Mayo, M.F., Carrigan, C.N., Erickson, H.K., Pinkas, J., et al. (2013). A novel anti-CD37 antibody-drug conjugate with multiple anti-tumor mechanisms for the treatment of B-cell malignancies. Blood *122*, 3500–3510. https://doi.org/10.1182/blood-2013-05-505685.

36. Smeland, E., Funderud, S., Ruud, E., Kiil Blomhoff, H., and Godal, T. (1985). Characterization of two murine monoclonal antibodies reactive with human B cells. Their use in a high-yield, high-purity method for isolation of B cells and utilization of such cells in an assay for B-cell stimulating factor. Scand. J. Immunol. *21*, 205–214. https://doi.org/10.1111/j.1365-3083.1985.tb01422.x.

37. Tislevoll, B.S., Hellesøy, M., Fagerholt, O.H.E., Gullaksen, S.-E., Srivastava, A., Birkeland, E., Kleftogiannis, D., Ayuda-Durán, P., Piechaczyk, L., Tadele, D.S., et al. (2023). Early response evaluation by single cell signaling profiling in acute myeloid leukemia. Nat. Commun. *14*, 115. https://doi.org/10.1038/s41467-022-35624-4.

38. Haferlach, T., Kohlmann, A., Wieczorek, L., Basso, G., Kronnie, G.T., Béné, M.C., De Vos, J., Hernández, J.M., Hofmann, W.K., Mills, K.I., et al. (2010). Clinical utility of microarray-based gene expression profiling in the diagnosis and subclassification of leukemia: report from the International Microarray Innovations in Leukemia Study Group. J. Clin. Oncol. *28*, 2529–2537. https://doi.org/10.1200/JCO.2009.23.4732.

39. Klein, H.U., Ruckert, C., Kohlmann, A., Bullinger, L., Thiede, C., Haferlach, T., and Dugas, M. (2009). Quantitative comparison of microarray experiments with published leukemia related gene expression signatures. BMC Bioinf. *10*, 422. https://doi.org/10.1186/1471-2105-10-422.

40. Metzelder, S.K., Michel, C., von Bonin, M., Rehberger, M., Hessmann, E., Inselmann, S., Solovey, M., Wang, Y., Sohlbach, K., Brendel, C., et al. (2015). NFATc1 as a therapeutic target in FLT3-ITD-positive AML. Leukemia *29*, 1470–1477. https://doi.org/10.1038/leu.2015.95.

41. Wouters, B.J., Löwenberg, B., Erpelinck-Verschueren, C.A.J., van Putten, W.L.J., Valk, P.J.M., and Delwel, R. (2009). Double CEBPA mutations, but not single CEBPA mutations, define a subgroup of acute myeloid leukemia with a distinctive gene expression profile that is uniquely associated with a favorable outcome. Blood *113*, 3088–3091. https://doi.org/10.1182/blood-2008-09-179895.

42. Rapin, N., Bagger, F.O., Jendholm, J., Mora-Jensen, H., Krogh, A., Kohlmann, A., Thiede, C., Borregaard, N., Bullinger, L., Winther, O., et al. (2014). Comparing cancer vs normal gene expression profiles identifies new disease entities and common transcriptional programs in AML patients. Blood *123*, 894–904. https://doi.org/10.1182/blood-2013-02-485771.

43. van Galen, P., Hovestadt, V., Wadsworth Ii, M.H., Hughes, T.K., Griffin, G.K., Battaglia, S., Verga, J.A., Stephansky, J., Pastika, T.J., Lombardi Story, J., et al. (2019). Single-Cell RNA-Seq Reveals AML Hierarchies Relevant to Disease Progression and Immunity. Cell *176*, 1265–1281.e24. https://doi.org/10.1016/j.cell.2019.01.031.

44. Heemskerk, M.H.M., Hoogeboom, M., de Paus, R.A., Kester, M.G.D., van der Hoorn, M.A.W.G., Goulmy, E., Willemze, R., and Falkenburg, J.H.F. (2003). Redirection of antileukemic reactivity of peripheral T lymphocytes using gene transfer of minor histocompatibility antigen HA-2-specific T-cell receptor complexes expressing a conserved alpha joining region. Blood *102*, 3530–3540. https://doi.org/10.1182/blood-2003-05-1524.

45. Jutz, S., Leitner, J., Schmetterer, K., Doel-Perez, I., Majdic, O., Grabmeier-Pfistershammer, K., Paster, W., Huppa, J.B., and Steinberger, P. (2016).

Assessment of costimulation and coinhibition in a triple parameter T cell reporter line: Simultaneous measurement of NF-kappaB, NFAT and AP-1. J. Immunol. Methods *430*, 10–20. https://doi.org/10.1016/j.jim.2016.01.007.

46. Hebbar, N., Epperly, R., Vaidya, A., Thanekar, U., Moore, S.E., Umeda, M., Ma, J., Patil, S.L., Langfitt, D., Huang, S., et al. (2022). CAR T cells redirected to cell surface GRP78 display robust anti-acute myeloid leukemia activity and do not target hematopoietic progenitor cells. Nat. Commun. *13*, 587. https://doi.org/10.1038/s41467-022-28243-6.

47. van den Ancker, W., Terwijn, M., Westers, T.M., Merle, P.A., van Beckhoven, E., Dräger, A.M., Ossenkoppele, G.J., and van de Loosdrecht, A.A. (2010). Acute leukemias of ambiguous lineage: diagnostic consequences of the WHO2008 classification. Leukemia *24*, 1392–1396. https://doi.org/10.1038/leu.2010.119.

48. Richard, A.C. (2022). Divide and Conquer: Phenotypic and Temporal Heterogeneity Within CD8+ T Cell Responses. Front. Immunol. *13*, 949423. https://doi.org/10.3389/fimmu.2022.949423.

49. Levy, M.Y., Jagadeesh, D., Grudeva-Popova, Z., Trněný, M., Jurczak, W., Pylypenko, H., André, M., Dwivedy Nasta, S., Rechavi-Robinson, D., Toffanin, S., et al. (2021). Safety and Efficacy of CD37-Targeting Naratuximab Emtansine PLUS Rituximab in Diffuse Large B-Cell Lymphoma and Other NON-Hodgkin'S B-Cell Lymphomas - a Phase 2 Study. Blood *138*, 526. https://doi.org/10.1182/blood-2021-145102.

50. Stathis, A., Flinn, I.W., Madan, S., Maddocks, K., Freedman, A., Weitman, S., Zucca, E., Munteanu, M.C., and Lia Palomba, M. (2018). Safety, tolerability, and preliminary activity of IMGN529, a CD37-targeted antibody-drug conjugate, in patients with relapsed or refractory B-cell non-Hodgkin lymphoma: a dose-escalation, phase I study. Invest. N. Drugs *36*, 869–876. https://doi.org/10.1007/s10637-018-0570-4.

51. Köksal, H., Dillard, P., Josefsson, S.E., Maggadottir, S.M., Pollmann, S., Fåne, A., Blaker, Y.N., Beiske, K., Huse, K., Kolstad, A., et al. (2019). Preclinical development of CD37CAR T-cell therapy for treatment of B-cell lymphoma. Blood Adv. *3*, 1230–1243. https://doi.org/10.1182/bloodadvances.2018029678.

52. Peeters, R., Cuenca-Escalona, J., Zaal, E.A., Hoekstra, A.T., Balvert, A.C.G., Vidal-Manrique, M., Blomberg, N., van Deventer, S.J., Stienstra, R., Jellusova, J., et al. (2022). Fatty acid metabolism in aggressive B-cell lymphoma is inhibited by tetraspanin CD37. Nat. Commun. *13*, 5371. https://doi.org/10.1038/s41467-022-33138-7.

53. Tcheng, M., Roma, A., Ahmed, N., Smith, R.W., Jayanth, P., Minden, M.D., Schimmer, A.D., Hess, D.A., Hope, K., Rea, K.A., et al. (2021). Very long chain fatty acid metabolism is required in acute myeloid leukemia. Blood *137*, 3518–3532. https://doi.org/10.1182/blood.2020008551.

54. Haubner, S., Perna, F., Köhnke, T., Schmidt, C., Berman, S., Augsberger, C., Schnorfeil, F.M., Krupka, C., Lichtenegger, F.S., Liu, X., et al. (2019). Coexpression profile of leukemic stem cell markers for combinatorial targeted therapy in AML. Leukemia *33*, 64–74. https://doi.org/10.1038/s41375-018-0180-3.

55. Zeng, A.G.X., Bansal, S., Jin, L., Mitchell, A., Chen, W.C., Abbas, H.A., Chan-Seng-Yue, M., Voisin, V., van Galen, P., Tierens, A., et al. (2022). A cellular hierarchy framework for understanding heterogeneity and predicting drug response in acute myeloid leukemia. Nat. Med. *28*, 1212–1223. https://doi.org/10.1038/s41591-022-01819-x.

56. Raglow, Z., McKenna, M.K., Bonifant, C.L., Wang, W., Pasca di Magliano, M., Stadlmann, J., Penninger, J.M., Cummings, R.D., Brenner, M.K., and Markovitz, D.M. (2022). Targeting glycans for CAR therapy: The advent of sweet CARs. Mol. Ther. *30*, 2881–2890. https://doi.org/10.1016/j.ymthe.2022.07.006.

57. Topp, M., and Feuchtinger, T. (2022). Management of Hypogammaglobulinaemia and B-Cell Aplasia. In The EBMT/EHA CAR-T Cell Handbook, N. Kröger, J. Gribben, C. Chabannon, I. Yakoub-Agha, and H. Einsele, eds. (Springer International Publishing), pp. 147–149. https://doi.org/10.1007/978-3-030-94353-0_28.

58. Jetani, H., Navarro-Bailón, A., Maucher, M., Frenz, S., Verbruggen, C., Yeguas, A., Vidriales, M.B., González, M., Rial Saborido, J., Kraus, S., et al. (2021). Siglec-6 is a novel target for CAR T-cell therapy in acute myeloid leukemia. Blood *138*, 1830–1842. https://doi.org/10.1182/blood.2020009192.

59. Sauer, T., Parikh, K., Sharma, S., Omer, B., Sedloev, D., Chen, Q., Angenendt, L., Schliemann, C., Schmitt, M., Müller-Tidow, C., et al. (2021). CD70-specific CAR T cells have potent activity against acute myeloid leukemia without HSC toxicity. Blood *138*, 318–330. https://doi.org/10.1182/blood.2020008221.

60. Smiljkovic, D., Herrmann, H., Sadovnik, I., Gamperl, S., Berger, D., Stefanzl, G., Eisenwort, G., Hoermann, G., Kopanja, S., Dorofeeva, Y., et al. (2023). Expression and regulation of Siglec-6 (CD327) on human mast cells and basophils. J. Allergy Clin. Immunol. *151*, 202–211. https://doi.org/10.1016/j.jaci.2022.07.018.

61. Corse, E., Gottschalk, R.A., Krogsgaard, M., and Allison, J.P. (2010). Attenuated T cell responses to a high-potency ligand in vivo. PLoS Biol. *8*, e1000481. https://doi.org/10.1371/journal.pbio.1000481.

62. Ghorashian, S., Kramer, A.M., Onuoha, S., Wright, G., Bartram, J., Richardson, R., Albon, S.J., Casanovas-Company, J., Castro, F., Popova, B., et al. (2019). Enhanced CAR T cell expansion and prolonged persistence in pediatric patients with ALL treated with a low-affinity CD19 CAR. Nat. Med. *25*, 1408–1414. https://doi.org/10.1038/s41591-019-0549-5.

63. Biasco, L., Izotova, N., Rivat, C., Ghorashian, S., Richardson, R., Guvenel, A., Hough, R., Wynn, R., Popova, B., Lopes, A., et al. (2021). Clonal expansion of T memory stem cells determines early anti-leukemic responses and long-term CAR T cell persistence in patients. Nat. Can. (Ott.) *2*, 629–642. https://doi.org/10.1038/s43018-021-00207-7.

64. Watanabe, N., Mo, F., Zheng, R., Ma, R., Bray, V.C., van Leeuwen, D.G., Sritabal-Ramirez, J., Hu, H., Wang, S., Mehta, B., et al. (2023). Feasibility and preclinical efficacy of CD7-unedited CD7 CAR T cells for T cell malignancies. Mol. Ther. *31*, 24–34. https://doi.org/10.1016/j.ymthe.2022.09.003.

65. Kim, M.Y., Yu, K.R., Kenderian, S.S., Ruella, M., Chen, S., Shin, T.H., Aljanahi, A.A., Schreeder, D., Klichinsky, M., Shestova, O., et al. (2018). Genetic Inactivation of CD33 in Hematopoietic Stem Cells to Enable CAR T Cell Immunotherapy for Acute Myeloid Leukemia. Cell *173*, 1439–1453.e19. https://doi.org/10.1016/j.cell.2018.05.013.

66. Casirati, G., Cosentino, A., Mucci, A., Salah Mahmoud, M., Ugarte Zabala, I., Zeng, J., Ficarro, S.B., Klatt, D., Brendel, C., Rambaldi, A., et al. (2023). Epitope editing enables targeted immunotherapy of acute myeloid leukaemia. Nature *621*, 404–414. https://doi.org/10.1038/s41586-023-06496-5.

67. Zunder, E.R., Finck, R., Behbehani, G.K., Amir, E.-A.D., Krishnaswamy, S., Gonzalez, V.D., Lorang, C.G., Bjornson, Z., Spitzer, M.H., Bodenmiller, B., et al. (2015). Palladium-based mass tag cell barcoding with a doublet-filtering scheme and single-cell deconvolution algorithm. Nat. Protoc. *10*, 316–333. https://www.nature.com/articles/nprot.2015.020.

68. Weber, L.M., Nowicka, M., Soneson, C., and Robinson, M.D. (2019). diffcyt: Differential discovery in high-dimensional cytometry via high-resolution clustering. Commun. Biol. *2*, 1–11. https://doi.org/10.1038/s42003-019-0415-5.

69. Van Gassen, S., Callebaut, B., Van Helden, M.J., Lambrecht, B.N., Demeester, P., Dhaene, T., and Saeys, Y. (2015). FlowSOM: Using self-organizing maps for visualization and interpretation of cytometry data. Cytometry A. *87*, 636–645. https://doi.org/10.1002/cyto.a.22625.

70. Whole transcriptome RNA sequencing on bone marrow and peripheral blood samples from patients with acute myeloid leukemia at diagnosis or relapse. - EGA European Genome-Phenome Archive https://ega-archive.org/datasets/EGAD00001004187.

71. Wolf, F.A., Angerer, P., and Theis, F.J. (2018). SCANPY: large-scale single-cell gene expression data analysis. Genome Biol *19*, 15. https://doi.org/10.1186/s13059-017-1382-0.

72. Virshup, I., Rybakov, S., Theis, F.J., Angerer, P., and Wolf, F.A. (2021). anndata: Annotated data. Preprint at bioRxiv, https://doi.org/10.1101/2021.12.16.473007.

73. L Lun, A.T., Bach, K., and Marioni, J.C. (2016). Pooling across cells to normalize single-cell RNA sequencing data with many zero counts. Genome Biol. *17*, 75. https://doi.org/10.1186/s13059-016-0947-7.

74. Lun, A.T.L., McCarthy, D.J., and Marioni, J.C. (2016). A step-by-step workflow for low-level analysis of single-cell RNA-seq data with Bioconductor. F1000Res. *5*, 2122. https://doi.org/10.12688/f1000research.9501.2.

75. Zheng, G.X.Y., Terry, J.M., Belgrader, P., Ryvkin, P., Bent, Z.W., Wilson, R., Ziraldo, S.B., Wheeler, T.D., McDermott, G.P., Zhu, J., et al. (2017). Massively parallel digital transcriptional profiling of single cells. Nat. Commun. *8*, 14049. https://doi.org/10.1038/ncomms14049.

76. McInnes, L., Healy, J., and Melville, J. (2020). UMAP: Uniform Manifold Approximation and Projection for Dimension Reduction. Preprint at arXiv. https://doi.org/10.48550/arXiv.1802.03426.

77. Diggins, K.E., Greenplate, A.R., Leelatian, N., Wogsland, C.E., and Irish, J.M. (2017). Characterizing cell subsets using marker enrichment modeling. Nat. Methods *14*, 275–278. https://doi.org/10.1038/nmeth.4149.

78. Schuyler, R.P., Jackson, C., Garcia-Perez, J.E., Baxter, R.M., Ogolla, S., Rochford, R., Ghosh, D., Rudra, P., and Hsieh, E.W.Y. (2019). Minimizing Batch Effects in Mass Cytometry Data. Front. Immunol. *10*. https://doi.org/10.3389/fimmu.2019.02367.

79. Loew, R., Heinz, N., Hampf, M., Bujard, H., and Gossen, M. (2010). Improved Tet-responsive promoters with minimized background expression. BMC Biotechnol. *10*, 81. https://doi.org/10.1186/1472-6750-10-81.

80. Shi, J., Wang, E., Milazzo, J.P., Wang, Z., Kinney, J.B., and Vakoc, C.R. (2015). Discovery of cancer drug targets by CRISPR-Cas9 screening of protein domains. Nat. Biotechnol. *33*, 661–667. https://doi.org/10.1038/nbt.3235.

CellPress
OPEN ACCESS

STAR★METHODS

KEY RESOURCES TABLE

REAGENT or RESOURCE	SOURCE	IDENTIFIER
Antibodies		
CD3-BV421(SK7)	BD Biosciences	Cat. No.563798; RRID:AB_2744383
CD3-BV605 (SK7)	BD Biosciences	Cat. No.563219; RRID:AB_2714001
CD4-BV421(RPA-T4)	BD Biosciences	Cat. No.562842; RRID:AB_2737832
CD4-BV605 (RPA-T4)	BD Biosciences	Cat. No.562658; RRID:AB_2744420
CD8-BV605 (RPA-T8)	BioLegend	Cat. No.301040; RRID:AB_2563185
CD8-PE-Cy7 (RPA-T8)	Thermo Fisher Scientific	Cat. No. 25-0088-42; RRID:AB_1659702
CD11b-PE (ICRF44)	BD Biosciences	Cat. No.555388; RRID:AB_395789
CD14-APC-Cy7 (MφP9)	BD Biosciences	Cat. No.557831; RRID:AB_396889
CD16-FITC (eBioCB16)	Thermo Fisher Scientific	11-0168-42; RRID:AB_10805747
CD19-BV421(HIB19)	BD Biosciences	Cat. No.562440; RRID:AB_11153299
CD19-PE (HIB19)	Thermo Fisher Scientific	Cat. No.12-0199-42; RRID:AB_1834376
CD20-APC	Thermo Fisher Scientific	Cat. No.17-0209-42; RRID:AB_10670628
CD33-BV421 (WM53)	BD Biosciences	Cat. No.562854; RRID:AB_2737405
CD34-PE-Cy7 (581)	BD Biosciences	Cat. No.560710; RRID:AB_1727470
CD34-APC (4H11)	Thermo Fisher Scientific	Cat. No.17-0349-42; RRID: AB_2016672
CD37-Af647 (M-B371)	BD Biosciences	Cat. No.561562; RRID:AB_10895803
CD37-Af647 (HH1)	Santa Cruz Biotechnology	Cat. No.sc-18881 AF647
Unconjugated anti-CD37 (HH1)	Santa-Cruz Biotechnology	Cat. No.sc-18881
CD38-APC-R7 (HIT2)	BD Biosciences	Cat. No.564979; RRID:AB_2744373
CD45-FITC (HI30)	BD Biosciences	Cat. No.555482; RRID:AB_395874
CD45RA-BV510 (HI100)	BD Biosciences	Cat. No.563031; RRID:AB_2722499
CD45RA- BB515 (HI100)	BD Biosciences	Cat. No.564552; RRID:AB_2738841
CD56-APC (NCAM16.2)	Thermo Fisher Scientific	Cat. No.17-0566-42; RRID:AB_2573148
CD69-PE-Cy5 (FN50)	BD Biosciences	Cat. No.555532; RRID: AB_395917
CD107a-PE-Cy5 (H4A3)	BD Biosciences	Cat. No.555802; RRID: AB_396136
CD107 APC (H4A3)	BD Biosciences	Cat. No.560664; RRID:AB_1727417

(*Continued on next page*)

Continued

REAGENT or RESOURCE	SOURCE	IDENTIFIER
CCR7 (CD197)-FITC (150503)	BD Biosciences	Cat. No.561271; RRID:AB_10561679
CD90-BV605 (5E10)	BD Biosciences	Cat. No.747750; RRID:AB_2872219
CD123-PE (7G3)	BD Biosciences	Cat. No.554529; RRID:AB_395457
CD123- PerCP-Cy^{TM}5.5 (7G3)	BD Biosciences	Cat. No.558714; RRID:AB_1645547
Anti-Human HLA-DR (L243) - 116Cd	BioLegend	Cat. No. 307651
Anti-Human Lineage Cocktail 2 (lin 2) *(CD3, CD14, CD19, CD20, CD56)*	BD Biosciences	Cat. No.643397
Murine IgG1 Isotype – Af647 (MOPC-21)	BioLegend	Cat. No.400136; RRID:AB_2832978
Mouse IgG1 Isotype –FITC (MOPC-21)	BD Biosciences	Cat. No.554679; RRID: AB_395505
Biotin-SP (long spacer) AffiniPure^{TM} F(ab')$_2$ Fragment Goat Anti-Mouse IgG, F(ab')$_2$ fragment specific	Jackson Immuno Research Laboratories	Cat. No.115-066-072; RRID: AB_2338583
Streptavidin-PE	BD Biosciences	Cat. No.554061; RRID:AB_10053328
Anti-HA tag antibody (mAb clone 2–2.2.14)	Invitrogen	Cat. No.26183; RRID:AB_10978021
Anti-CD37 antibody (mAb clone E4K2M)	Cell Signaling Technology	Cat. No.46894;
Goat anti-rabbit IgG antibody conjugated to horseradish peroxidase	Invitrogen	Cat. No.31460; RRID:AB_228341
anti-CD3 (OKT3), Biotin	Thermo Fisher Scientific	Cat. No.13-0037-82; RRID:AB_1234955
anti-CD28 (CD28.6), Biotin	Thermo Fisher Scientific	Cat. No.13-0289-82; RRID:AB_466415
Anti Biotin (1D4-C5) - 143ND	Standard BioTools	Cat. No.3143008B
Anti-Human CD3 (UCHT1) – 170Er	Standard BioTools	Cat. No.3170001B
Anti-Human CD3 (UCHT1) – 111Cd	BioLegend	Cat. No.300443
Anti-Human CD4 (RPA-T4) –145ND	BioLegend	Cat. No.300541
Anti-Human CD7 (CD7-687) –114Cd	BioLegend	Cat. No.343102
Anti-Human CD8 (HIT8a) – 139La	BioLegend	Cat. No.300902
Anti-Human CD11b (ICRF44) - 209Bi	Standard BioTools	Cat. No.3209003B
Anti-Human CD11c (L243) – 174Yb	BioLegend	Cat. No.337221
Anti-Human CD14 (M5E2) –160Gd	Fluidigm	Cat. No.3160001B
Anti-Human CD16 (3G8) - 148ND	Fluidigm	Cat. No. 3148004B
Anti-Human CD19 (HIB19) - 165Ho	Standard BioTools	Cat. No.3165025B
Anti-Human CD19 (HIB19) – 141Pr	BioLegend	Cat. No. B318939
Anti-Human CD25 (2A3) - 169Tm	Standard BioTools	Cat. No.3169003B
Anti-Human CD27 (L128) - 162Dy	Standard BioTools	Cat. No.3162009B
Anti-Human CD28 (CD28.2) - 160Gd	Standard BioTools	Cat. No.3160003B
Anti-Human CD33 (WM53) - 158Gd	Standard BioTools	Cat. No.3158001B
Anti-Human CD34 (4H11) – 145ND	eBioscience	Cat. No.15236917
Anti-Human CD34 (581) - 113Cd	BioLegend	Cat. No. 343531
Anti-Human CD38 (HIT2) - 144ND	Standard BioTools	Cat. No.3144014B
Anti-Human CD38 (HIT2) –144ND	Standard BioTools	Cat. No.3144014C
Anti-Human CD44(BJ18) –166Er	Fluidigm	Cat. No.2103505-29
Anti-Human/Mouse CD44 (IM7) - 171Yb	Standard BioTools	Cat. No.3171003B

(*Continued on next page*)

Continued

REAGENT or RESOURCE	SOURCE	IDENTIFIER
Anti-Human CD45 (HI30) - 89Y	Standard BioTools	Cat. No.3089003B
Anti-Mouse CD45 (30-F11) - 147Sm	Standard BioTools	Cat. No.3147003B
Anti-Human CD45RA (Hi100)	Fluidigm	Cat. No.3143006B
Anti-Human CD45RO (UCHL1) - 149Sm	Standard BioTools	Cat. No.3149001B
Anti-Human CD45RA-BV510 (HI100)	BD Biosciences	Cat. No.563031
Anti-Human CD45RA-FITC (HI100)	BD Biosciences	Cat. No.561882
Anti-Human CD45RA (HI100) - 153Eu	Standard BioTools	Cat. No.3153001B
Anti-Human CD56-APC (NCAM16.2)	BD Biosciences	Cat. No.341025
Anti-Human CD56 (B159) –155Gd	Standard BioTools	Cat. No.3155008B
Anti-Human CD57 (HNK-1)	BioLegend	Cat. No.359602
Anti-Human CD64 (10,1) – 146ND	Standard BioTools	Cat. No.3146006C
Anti-Human CD66b (8OH3) –152Sm	Fluidigm	Cat. No.3152011B
Anti-Human CD73(AD2) –172Yb	Abcam	Cat. No.ab130451
Anti-Human CD90 (5E10) –159Tb	Fluidigm	Cat. No.3159007C
Anti-Human CD95/Fas (DX2) - 152Sm	Standard BioTools	Cat. No.3152017B
Anti-Human CD105 (43A3) 163Dy	Fluidigm	Cat. No.3163005C
Anti-Human CD117/cKit (YB5B8) –168Er	Invitrogen	Cat. No. 14-1179-82
Anti-Human CD123 (6H6) – 112Cd	BioLegend	Cat. No 306027
Anti-Human CD123 (6H6) - 151Eu	Standard BioTools	Cat. No.3151001B
Anti-Human CD127 (A019D5) - 168Er	Standard BioTools	Cat. No.3168017B
Anti-Human CD134/OX40 (ACT35) - 142ND	Standard BioTools	Cat. No.3142018B
Anti-Human CD137/4-1BB (4B4-1) - 173Yb	Standard BioTools	Cat. No.3173015B
Anti-Human CD152/CTLA-4 (14D3) - 161Dy	Standard BioTools	Cat. No.3161004B
Anti-Human CD184/CXCR4 (12G5) - 175Lu	Standard BioTools	Cat. No.3175001B
Anti-Human CD185/CXCR5 (RF8B2) - 164Dy	Standard BioTools	Cat. No.3164029B
Anti-Human CD196/CCR6 (G034E3) - 141Pr	Standard BioTools	Cat. No.3141003A
Anti-Human CD197/CCR7 (G043H7) - 167Er	Standard BioTools	Cat. No.3167009A
Anti-Human CD223/LAG-3 (11C3C65) - 150ND	Standard BioTools	Cat. No.3150030B
Anti- Human/Mouse/Rat CD278/ICOS (C398.4A) - 148ND	Standard BioTools	Cat. No.3148019B
Anti-Human CD279/PD-1 (EH12.2H7) - 155Gd	Standard BioTools	Cat. No.3155009B
Anti-Human CD300e (233810) –173Yb	R&D	Cat. No.MAB2705
Anti-Human CD366/TIM-3 (F38-2E2) - 154Sm	Standard BioTools	Cat. No.3154010B
Anti-Human FLT3 (S18)-161Dy	BioLegend	Cat. No. 313302
Anti-Human Caspase 3(Cleaved) (D3E9)	Fluidigm	Cat. No. 3142004C
Anti-Human CSF1R (9-4D2-1E4) –170Er	BioLegend	Cat. No. 347302
AntiHuman Cyclin B1 (GNS-1)-164Dy	Fluidigm	Cat. No. 3165011B
Anti-GFP (FM2-64G)	BioLegend	Cat. No.338002
Anti-Human Histone H3 (D1H2) - 176Yb	Standard BioTools	Cat. No.3176016A
Anti-Human HLA-DR (L243) - 174Yb	Standard BioTools	Cat. No.3174001B
Anti-Human Ki-67 (B56) - 172Yb	Standard BioTools	Cat. No.3172024B
Anti-Human NRAS Q61 mut (EPR20278) – 154ND	Abcam	Cat. No.ab242415

(*Continued on next page*)

Continued

REAGENT or RESOURCE	SOURCE	IDENTIFIER
Anti-Human pERK ½ [T202/Y204] (D13.14.4) –167Er	Fluidigm	Cat. No.3167005C
Anti-Human pRB(S807/811)(J112-906) – 150ND	Fluidigm	Cat. No.3150013A
Anti-Human pStat5 (Y694)/47) –147 Nd	Fluidigm	Cat. No.3150005A
Anti-Human RUNX1, RUNX2 and RUNX3 (EPR3099) 153Eu	Abcam	Cat. No.ab220117
Anti-Human TIGIT (MBSA43) - 159Tb	Standard BioTools	Cat. No.3159038B
**Bacterial and virus strains**		
NEB® 5-alpha Competent E. coli (High Efficiency) | DH5α	New England Biolab	C2987H
**Biological samples**		
Human peripheral blood mononuclear cells (PBMCs)	Healthy donors	(REK vest 2012/2247)
Peripheral blood samples	AML patients	(REK VEST 2015/1759)
AML biobank	AML patients	(REK 2022/48847), REK VEST (REK III nr. 060.02 and 059.02)) and NDPA 02/118-5
Bone marrow samples	AML patients	(2015/1012/REK sør-øst D)
Bone marrow with matched-blood samples	Healthy donors at bone marrow donation	N/A
AML patient-derived BMMCs	AML patients	N/A
Patient-derived xenografts (PDX)	AML patients	(FOTS ID 29646)
**Chemicals, peptides, and recombinant proteins**		
Fetal bovine serum (FBS)	Gibco	Cat. No.10500-064
Human serum	PAN Biotech	Cat. No.P40-2702HI
Human serum albumin	Octa pharma	N/A
Recombinant human IL-2 (Proleukin)	Clinigen	N/A
Gentamycin	Gibco	Cat. No.15750-037
BD Fc Block	BD Biosciences	Cat. No.564220
Deoxyribonuclease (DNAse) I from bovine pancreas	Sigma Aldrich	Cat. No.DN25
RIPA buffer	Thermo Fisher Scientific	Cat. No.89900
β-mercaptoethanol	Sigma Aldrich	Cat. No.M3148
Retronectin	Takara Bio.	Cat. No.T100B
Dasatinib	LC Laboratories	Cat. No.D-3307
Neuraminidase	Roche	Cat. No. 11585886001
Puromycin	Gibco	Cat. No.A1113802
Accutase	Thermo Fisher Scientific	Cat. No.A1110501
GolgiStop	BD Biosciences	Cat. No.554724
GolgiPlug	BD Biosciences	Cat. No.555029
D-Luciferin potassium salt	Revvity	Cat. No.122799
CFSE	Thermo Fisher Scientific	Cat. No.C34554
Cell Trace Violet	Thermo Fisher Scientific	Cat. No.C34557
Propidium Iodide	Thermo Fisher Scientific	Cat. No.R37169
Count Bright^{TM} Absolute Counting Beads	Thermo Fisher Scientific	Cat. No.C36950
Stable-Lyse V2 buffer	Smart Tube, Inc.	STBLYSE2-250
Stable-Store V2 buffer	Smart Tube, Inc.	STBLSTORE2-1000
FcR blocking reagent, human	Miltenyi Biotec	Cat.No.130-059-901

(*Continued on next page*)

CellPress
OPEN ACCESS

Continued

REAGENT or RESOURCE	SOURCE	IDENTIFIER
CD16/CD32 Monoclonal Antibody (93) (FcR block)	eBioscience	Cat.No.15288387
MaxPar Cell Acquisition Solution Plus	Standard Bio-Tools	Cat.No. 201244
MaxPar phosphate-buffered saline (PBS)	Standard BioTools	Cat.No. 201058
MaxPar Cell Staining Buffer (CSB)	Standard BioTools	Cat.No. 201068
Paraformaldehyde (PFA)	Alfa Aesar	Cat.No. 43368
Dimethyl sulfoxide (DMSO)	Sigma Aldrich	Cat.No.D5879
Heparin-Natrium-5000 - Ratiopharm®	Ratiopharmn GmbH	N68743.08
Methanol	Sigma Aldrich	Cat.No. 32213-M
Phytohemagglutinin	Sigma Aldrich	Cat.No. 61764
Cell-ID^{TM} Intercalator-Ir - 500 μM	Standard BioTools	Cat.No. 201192B
EQ^{TM} Six Element Calibration Beads	Standard BioTools	Cat.No. 201245
Maxpar MCP9 Antibody Labeling Kit, 111Cd–4 Rxn	Standard BioTools	Cat.No. 201111A
Maxpar MCP9 Antibody Labeling Kit, 113Cd–4 Rxn	Standard BioTools	Cat.No. 201113A
Maxpar MCP9 Antibody Labeling Kit, 166Cd–4 Rxn	Standard BioTools	Cat.No. 201116A
Maxpar® X8 Antibody Labeling Kit, 145Nd—4 Rxn	Standard BioTools	Cat.No. 201145A
Maxpar® X8 Antibody Labeling Kit, 146Nd—4 Rxn	Standard BioTools	Cat.No. 201146A
Maxpar® X8 Antibody Labeling Kit, 156Gd—4 Rxn	Standard BioTools	Cat.No. 201156A
Maxpar® X8 Antibody Labeling Kit, 156Gd—4 Rxn	Standard BioTools	Cat.No. 201156A
**Critical commercial assays**		
QIFIKIT®, Series of coated beads, Flow Cytometry, 10 calibrations	Agilent DGG Norge AS	Cat. No.K007811-8
Cell-ID 20-Plex Palladium Barcoding Kit	Standard BioTools	Cat. No.201060
Bio-Plex Pro Human Cytokine 17-plex assay	Bio-rad	Cat. No. M5000031YV
Pan Monocyte Isolation Kit human	Miltenyi Biotec	Cat. No. 130-096-537
**Deposited data**		
Raw data "CD37 a safe Chimeric Antigen Receptor target to treat acute myeloid leukemia"	Mendeley Data	https://doi.org/10.17632/rdkg26mfjw.1
**Experimental models: Cell lines**		
HEK-P (Phoenix-AMPHO)	LCG Genomics GMBH	ATCC-CRL-3213
BL-41	Leibniz Institute DSMZ-German Collection	ACC 160
GRANTA-519	J. Myklebust (Oslo University Hospital)	PubMed: 30979721
Daudi	J. Myklebust (Oslo University Hospital)	PubMed: 30979721
K-562	ATCC	CCL-243
MOLM-13	Leibniz Institute DSMZ-German Collection	ACC 554
SKM-1	Leibniz Institute DSMZ-German Collection	ACC 547
HL-60	Leibniz Institute DSMZ-German Collection	ACC 3
U-937	Sigma-Aldrich Norway AS	85011440-1VL
MV4-11	ATCC	CRL-9591

(*Continued on next page*)

Continued

REAGENT or RESOURCE	SOURCE	IDENTIFIER
HEL	Leibniz Institute DSMZ-German Collection	ACC 11
Jurkat76 (J76)	M. Heemskerk (Leiden University Medical Center, The Netherlands)	PubMed: 12869497
Experimental models: Organisms/strains		
NOD-*Prkdc*^{scid} -*IL2rg*^{Tm1}/Rj (NXG) mice	The Jackson Laboratory	SCANBUR AS
Software and algorithms		
GraphPad Software 8.0.2	GraphPad software	https://www.graphpad.com
FlowJo 10.7.1	FlowJo, LLC	https://www.flowjo.com/
BioRender	BioRender	https://www.biorender.com/
Python 3		N/A
Cytobank	Beckman Coulter	https://premium.cytobank.org/
R 4.2.0	R Core Team	https://www.r-project.org/
RStudio 2022.07.1 + 554	Posit	https://posit.co/products/open-source/rstudio/
CyTOF XT mass cytometer software 8.1.0 + 18524	Standard BioTools	N/A
IVIS®-200 imaging system	Perkin Elmer	https://www.perkinelmer.com/
MATLAB R2013a	MathWorks	https://www.mathworks.com/products/matlab.html
MATLAB application - single-cell debarcoder	Zunder et al.[67]	https://doi.org/10.1038/nprot.2015.020
R package - cytoBatchNorm 0.0.0.9001	GitHub/i-cyto	https://github.com/i-cyto/cytoBatchNorm
R package - CATALYST 1.22.0	GitHub/CATALYST	https://github.com/HelenaLC/CATALYST
R package - diffcyt 1.18.0	GitHub/lmweber[68]	https://github.com/lmweber/diffcyt
R package - flowCore 2.10.0	Bioconductor	https://www.bioconductor.org/packages/release/bioc/html/flowCore.html
R package - FlowSOM 2.6.0	Van Gassen S et al.[69]	https://bioconductor.org/packages/release/bioc/html/FlowSOM.html
R package - Premessa 0.3.4	GitHub/Parker Institute for Cancer Immunotherapy	https://github.com/ParkerICI/premessa

RESOURCE AVAILABILITY

Lead contact

Requests for further information and reagents should be directed to and will be fulfilled by the Lead Contact, Sébastien Wälchli (sebastw@rr-research.no).

Materials availability

Materials created in this study will be available for the scientific community by contacting the corresponding author and completion of a material transfer agreement.

Data and code availability

- Raw data have been deposited at Mendeley repository and are publicly available as of the date of publication. DOIs are listed in the key resources table.
- This paper does not report original code.
- Any additional information required to reanalyze the data reported in this work paper is available from the lead contact upon request.

EXPERIMENTAL MODEL AND STUDY PARTICIPANT DETAILS

Cell lines

The human cell lines HEK-P, BL-41, GRANTA-519, Daudi, K-562, MOLM-13, SKM-1, HL-60, U-937, MV4-11, and HEL were obtained from DSMZ and ATCC. The Jurkat76 was a kind gift from M. Heemskerk (Leiden University Medical Center, The Netherlands). All cell

lines were routinely tested by PCR for the presence of mycoplasma (Minerva Biolabs). Cells were maintained in RPMI-1640 (PAA Laboratories) supplemented with 10% fetal bovin serum (FBS; Gibco) and 50 μg/mL gentamycin (Gibco) (complete RPMI medium) in a humidified atmosphere at 37°C, 5% CO_2.

Human samples

All patients' samples were collected using a written informed consent. The written informed consent of biobanked material to be used for *in vitro* and *in vivo* research in Oslo and Bergen is based on the legislations and in accordance with the Declaration of Helsinki. The study was conducted after approval from the Regional Committees for Medical and Health Research Ethics (https://www.forskningsetikk.no/en/about-us/our-committees-and-commission/rek/). BM samples were collected from twenty-five consenting AML patients (2015/1012/REK sør-øst D). Two BM with matched-blood samples were obtained from healthy donors at BM donation. Buffy coats were purchased from the hospital. Healthy PBMCs and healthy/AML patient-derived BMMCs were isolated by density gradient and cryopreserved in liquid nitrogen. Peripheral blood samples were collected from 59 informed and consenting AML patients (REK VEST 2015/1759) and healthy peripheral blood samples were collected from 5 healthy consenting donors according (REK vest 2012/2247). The PBMCs from PB samples in this cohort (n = 64) were isolated by density gradient centrifugation and cryopreserved before mass cytometric assessment.

Mouse xenograft studies

The study design was approved by the Norwegian Food Safety Authority (FOTS ID 29646). NOD-Prkdc^{scid} -IL2rg^{Tm1}/Rj (NOD xenograft gamma, NXG) mice were bred in-house and maintained in pathogen-free conditions under an approved institutional animal care protocol. Pilot studies were conducted for each xenograft to evaluate the time-to-engraftment, tumor growth and the onset of clinical signs to human endpoints. Six-to 10-week-old NXG mice were injected intravenously (i.v) with either 5x10^5 U-937 GFP-Luc$^+$, 5x10^3 MOLM-13 GFP-Luc$^+$ or 1x10^6 primary AML PDX GFP-Luc$^+$ cells at 3-, 7- or 7/14-day prior T cell infusion, respectively. Mice were injected intraperitoneally with 200 μL of 20 mg/mL Xenolight D-Luciferin potassium salt to confirm engraftment using *in vivo* imaging system (IVIS spectrum, PerkinElmer). Next, mice were allocated to each treatment group, such that each group had a similar representation of engraftment levels. At the indicated time point, mice were then infused with effector T cells adjusted to the same percentage of CAR-expressing cells with Mock T cells. IVIS analysis was repeated weekly, and the condition of the mice was assessed at least twice weekly.

METHOD DETAILS

Gene expression analysis

The transcriptomic datasets were downloaded from Bloodspot,[66] the GDC data portal and the European Genome-Phenome Archive (EGA).[70] From Bloodspot, curated and normalized AML microarrays were acquired from 2 datasets. First set was the MILE study[45] (n = 254; GSE13159) and the second set was a pool of different studies (n = 2074; GSE13159, GSE15434, GSE61804, GSE14468 and TCGA). Human healthy hematopoietic cells microarrays were from GSE42519 (n = 29). From the GDC data portal, we used RNA-seq data from TCGA (release July 27, 2022), project Acute Myeloid Leukemia (LAML; n = 150). To this end, RNA-seq raw data and the corresponding patient clinical data were downloaded using TCGAbiolinks.[70] Analysis was performed using R (https://www.r-project.org/) using Bioconductor (https://www.bioconductor.org) packages. Harmonized database (https://portal.gdc.cancer.gov/) which is mapped to the reference genome GRCh38 (hg38) was used. Raw count normalization, low-count gene prefiltering, experiment design and differential expression analysis were performed using DESeq2.[71] In this analysis, the clinical factor of prognosis was used as a specific coefficient in the design experiment. For FDR correction, adjusted p values lower than 0.05 were selected. 'Pheatmap' and 'EnhancedVolcano' packages were used to visualize the differential expression analyses. For the European Genome-Phenome Archive (EGA), data were obtained from EGAD00001004187, DAC: EGAC00001000956 datasets. This dataset contains 100 sequences cryopreserved bone marrow and peripheral blood samples from patients with AML with 10–90% blasts were selected from the biobank of the Department of Hematology of Leiden University Medical Center (LUMC). For the CD37 transcript in total 88 samples were compared, where of 75 samples were AML bone marrows and 13 normal bone marrows. Counts were quantified from fastq files using Kallisto software (https://pachterlab.github.io/kallisto/about) v0.46.1 and imported into R using tximport package version 1.26.1. The data was normalized with TMM-Normalization in edgeR package, version 3.40.2, and later log2 transformed.

Single cell transcriptomic analysis

All preprocessing and analysis steps of scRNA-seq data were run in Python 3 using Scanpy[71] v.1.4.6 to 1.9.1 and anndata[72] v.0.7.1 to 0.8.0 unless otherwise stated. All scRNA-seq figures were plotted using matplotlib and seaborn. We obtained raw, annotated count data of healthy bone marrow cells from van Galen et al.[43]. from Gene Expression Omnibus (GSE116256). Here, we excluded individual AML916, as it had a mixed AML phenotype expressing markers of stem cells, myeloid, T and B lineages. Barcodes were filtered for each sample for high-quality cells based on the total distributions of unique molecular identifier counts and genes, excluding cells with a fraction of mitochondria-encoded genes over 20%. Barcodes that could not be confidently assigned to either healthy or tumor cells were discarded. Genes detected in less than 20 cells were excluded from further analyses. The resulting count matrix was used

for normalization. Unique molecular identifier counts of each cell were normalized using the SCRAN algorithm as implemented in the R-based package.[73,74] The top 4,000 variable genes were identified based on normalized dispersion, as described previously,[75] using Scanpy's pp.highly_variable_genes. Principal-component analysis dimension reduction was performed by computing 15 principal components on highly variable genes using Scanpy's pp.pca. Next, a neighborhood graph was computed on the first 50 harmony-adjusted principal components using Scanpy's pp. neighbors with 15 neighbors. For two-dimensional visualization, embedding the neighborhood graph via UMAP[76] was done by running Scanpy's tl.umap with an effective minimum distance between embedded points of 0.5.

Mass cytometry

Cryopreserved PBMCs were fixed with 2% PFA and barcoded using Cell-ID 20-Plex Palladium Barcoding Kit according to the manufacturer's protocol. The samples were distributed in four barcode pools where each barcode contained up to 20 samples. All antibodies used in this study were either purchased pre-conjugated from Standard Bio-Toolsor were conjugated in-house using X8 MaxPar conjugation kits according to the manufacturer's protocol. Aliquots of 1.5x10^6 – 3.0 x 10^6 cells were blocked using an anti-human FcR blocking reagent (130-059-901, Miltenyi Biotec) and stained with antibody panel (Table S2) mastermixes in a staining volume of 100 μL per 3.0 x 10^6 cells for 30 min at room temperature on shaker. The antibody dilutions used are a result of previous titration experiments and peer recommendation. The cells were permeabilized for 10 min on ice using pure methanol (−20°C, 100%), treated with heparin (100 IU/mL, 20 min) and subsequently stained with intracellular antibodies (30 min, room temperature on shaker). To ensure the identification of cells, DNA was labelled with iridium-191/193 by incubation in 0.1 nM Ir-nucleic acid intercalator (Standard Biotools, San Francisco, CA, USA) diluted in MaxPar PBS containing 4% PFA (Alfa Aesar, 16% PFA, methanol-free) overnight at 4°C. The samples were washed, strained and pelleted prior to acquisition on the XT mass cytometer (Standard Bio-Tools). The Automatic sampling carousel on the XT mass cytometer resuspends the samples in MaxPar Cell Acquisition Solution Plus supplemented with a 1:10 dilution of the EQ Six Element calibration beads (Standard Bio-Tools). The acquisition rate was kept below 300 cells per second to reduce the chances of clogging and doublet acquisition. The MATLAB barcode de-convolution tool was used for de-barcoding samples. Sample preprocessing was performed with Premessa. Data analysis was conducted in Cytobank.org and in the statistical programming tool RStudio. We performed dimensionality reduction using the tSNE-CUDA algorithm and unsupervised self-organizing clustering using FlowSOM in Cytobank.[69] The FlowSOM included 2 286 000 cells distributed into 196 clusters and 10 meta clusters using 23 phenotypic markers; CD45, CD3, CD34, CD123, CD7, HLA-DR, CD8a, CD19, CD45RA, CD38, CD4, CD64, CD16, CD56, CD33, CD90, CD14, CD45RO, CD44, CD25, CD300e, CD11c and CD11b (Table S2). Marker enrichment modeling (MEM) heatmaps were constructed in RStudio from FlowSOM-identified meta clusters. The MEM R script produces a heatmap of MEM values with a summary of feature enrichment as the population (row) names. The + or −value provided along with the marker name is converted to a −10 to +10 scale and rounded to the nearest integer. The MEM-created population labels aids in identification and correct naming of rare phenotypic clusters in complex sample datasets. The patients' relative meta cluster abundances were depicted by stacked bar plots using RStudio.[77]

To study T cells in mice, CAR T cells were fixed using Stable-Lyse V2 and Stable-Store V2 (Smart Tube Inc., USA) according to the manufacturer's protocol, and cryopreserved at −80°C in 10% V/V FBS in DMSO. At endpoint, the spleen and bone marrow cells from femurs were harvested from the euthanized experimental mice. Single-cell suspensions from murine spleen were obtained by mechanical maceration using glass microscopy slides in a Petri dish containing a small amount of cell culture medium. The bone marrow cells were flushed out using cell culture medium delivered by syringe. Suspension materials were filtered through 40 μm cell strainers, fixed and cryopreserved in the same manner as describe above. All antibodies were either sourced in their metal-conjugated form directly from Standard Bio-Tools Inc. or conjugated in-house using X8 MaxPar conjugation kits (Standard Bio-Tools Inc., USA) according to the manufacturer's protocol.

Samples were slowly thawed at 4°C, washed, and barcoded using Cell-ID 20-Plex Palladium Barcoding Kits (Standard BioTools Inc., USA) into five batches of up to 20 palladium-barcoded samples. Each barcoded sample contained up to 4.0*10^6 cells. All samples from the same barcode batch were pooled together to enable uniform and consistent antibody staining. Every batch contained aliquots of two types of anchor samples to be used for the elimination of technical variability due to differences in sample staining conditions by means of batch correction. One type of anchor sample consisted of a mix of CD37-targeting CAR T cells, GFP-positive AML cells, phytohemagglutinin-stimulated healthy human donor PBMCs, and unstimulated healthy donor PBMCs. The composition of the mix would enable the detection of a "positive" signal on all markers present in the panel, which would, in turn, enable batch correction to be performed on each. The second type of anchor consisted solely of healthy human donor PBMCs and was meant to serve as a control for the results of batch correction. The functionality of the anchor samples and of the antibody panel was mutually validated.

Prior to surface antibody staining, human and murine Fc receptors were blocked using human (cat. no. 130-059-901, Miltenyi Biotec, Germany) and murine (cat.no. 15288387, eBioscience, USA) FcR blocking reagents and incubated with a biotinylated goat anti-murine-IgG antibody (cat. no. 115-066-072, Jackson ImmunoResearch Laboratories Inc., UK), which served as a primary antibody for marking the murine part of the chimeric antigen receptor of the anti-CD37 CAR T cells. After several washes, the cells were surface-stained on a shaker for 30 min at room temperature with a mix of the appropriate surface-antigen-targeting antibodies from the panel (Table S4) at a concentration of 3 x 10^6 cells per 100 μL of staining volume. Following several washes, the cells were permeabilized overnight at −20°C in 100% methanol. The following day, cells were washed, filtered through a 35 μm mesh, and stained with

intracellular-antigen-targeting antibodies from the panel (Table S4) under the same conditions as when stained with the surface-antigen-targeting antibodies. Excess antibody was washed away, and the cells' DNA was labeled with iridium by incubation in Cell-ID Intercalator-Ir (Standard Bio-Tools Inc., USA) diluted to 250 nM in MaxPar Cell Staining Buffer (Standard Bio-Tools Inc., USA) containing 2.9% PFA for 10 min at room temperature. The purpose of this DNA labeling was to aid in the detection of cellular events during data acquisition. Stained cells were washed and cryopreserved at -80°C in 10% V/V FBS in DMSO. On the day of data acquisition, cells were thawed, filtered through a 35 μm mesh, counted on a Countess II automated cell counter (Thermo Fisher Scientific Inc., USA), and distributed into 5 mL polypropylene tubes in such a way that a concentration of 8×10^5 cells per mL would be achieved upon resuspension by the mass cytometer in order to keep the acquisition rate below 300 events per second. This event rate was deemed to present an optimal balance of data acquisition speed and clog avoidance. For acquisition, cells were centrifuged into pellets.

Phenotypic data was acquired on the CyTOF XT Mass Cytometer (Standard Bio-Tools Inc., USA). Cell pellets were resuspended by the mass cytometer using MaxPar Cell Acquisition Solution Plus (Standard Bio-Tools Inc., USA) containing a 1:10 dilution of EQ Six Element Calibration Beads (Standard Bio-Tools Inc., USA). Signal decline over time was corrected internally by the CyTOF XT mass cytometer software using the six-element bead data. Sample data was debarcoded using the MatLab-based debarcoding tool developed by Eli Zunder.[67] Certain samples occupied multiple barcode slots due to the large number of cells they contained. All parts of their data were concatenated back together into one file using the Premessa R-package. Files containing data on over 52 million events were uploaded to Cytobank.org for cleanup by manual gating of event length, EQ bead and doublet exclusion, and Gaussian gating. Human cells were manually gated out according to expression of human and murine CD45. Cleaned up files containing data from approximately eight million human cells were downloaded from Cytobank.org and subjected to batch correction on the basis of the anchor samples in each barcode batch using a graphical-user-interface-based implementation (cytoBatchNorm - unpublished as of yet) of the CytofBatchAdjust batch correction algorithm.[78]

The diffcyt R package[68] was mainly used for data analysis. Its internal implementations of the flowCore, CATALYST and FlowSOM[69] packages were used for the clustering, visualization and quality control of the data. CAR T cells were separated out of the human cell parent population by clustering on the expression of the hCD45, CD3, CD123 and CD45RA markers into 64 clusters, and manual metaclustering into AML and CAR T cellT-cell metaclusters. In the same manner CAR T cellT-cell subsets were inferred from the results of clustering of the CAR T cellT-cell data into 100 clusters on the expression of the CD4, CD8, CD45RA, CD45RO, CD27, CD95, CD184, CD185, CD196 and CD197 markers, and manual metaclustering. Each step of clustering and metaclustering was thoroughly checked using heatmaps, UMAPs, biaxial plots. The metacluster assignment of each individual cluster was verified on Cytobank.org. To get deeper insight into the data of the CAR T cells, the CAR T cells were metaclustered into several nested sets of metaclusters of different granularities. The exploration of the data to find significant differences in abundances or cell state marker signal expression in CAR T cellT-cell metaclusters between different sets of samples was done on Cytobank.org using the website's built-in data plotting and exploration tools. All *p*p-values were inferred by paired or unpaired student's t-tests and one-way ANOVAs. *p*P-values were corrected for multiple hypothesis testing using the Benjamini-Hochberg method and Tukey's range test.

DNA constructs

The HH1 antibody-based CD37CAR and the fmc63-based CD19CAR designs were previously described.[32] The humanized mouse anti-human CD33 gemtuzumab ozogamicin sequence from https://go.drugbank.com/drugs/DB00056 and was designed as the other scFv: [leader sequence-light chain-$(G_4S)_4$-heavy chain] fused to the CD8 hinge and transmembrane domains (amino acid 128–210, UniProt P01732) followed by the 4-1BB intracellular costimulation domain (amino acid 208–255, UniProt P07011) and the CD3ζ signaling unit (amino acid 52–164, UniProt P20963) (see Figure S6). CD33CAR was designed as above followed by a T2A ribosome skipping sequence and a truncated sequence of CD34 (CD34t; UniProt P28906, Figure S4) downstream of the CD3ζ signaling unit. Codon optimized sequences were purchased (Eurofins, Erlangen Germany), cloned into the Gateway system pENTR (Thermo Fisher Scientific) and further subcloned into the retroviral vector pMP71. The GFP-firefly luciferase (GFP-Luc) fusion protein coding sequence (a kind gift from Rainer Löw)[79] was incorporated into pMP71 and used to stably transduce target cell lines as reported in.[32] The full-length CD37 isoform-1 (UniProt P11049-1), isoform-2 (UniProt P11049-2) and isoform-3 (UniProt P11049-3) were ordered as codon optimized DNA (Eurofins) and cloned into pMP71.

Immunophenotyping by flow cytometry

The following anti-human antibodies were purchased from BD Biosciences unless specified: CD3-BV421 and BV605 (SK7), CD4-BV421 and BV605 (RPA-T4), CD8-BV605 and PE-Cy7 (RPA-T8 from BioLegend and Thermo Fisher Scientific), CD11b-PE (ICRF44), CD14-APC-Cy7 (MφP9), CD19-BV421 (HIB19) and PE (HIB19 from Thermo Fisher Scientific), CD33-BV421 (WM53), CD34-PE-Cy7 and APC (581 and 4H11 from Thermo Fisher Scientific), CD37-Af647 (M-B371 and HH1 from Santa Cruz Biotechnologiy), CD38-APC-R7 (HIT2), CD45-FITC (HI30), CD45RA-BV510 and BB515 (HI100), CD56-APC (NCAM16.2 from Thermo Fisher Scientific), CD69-PE-Cy5 (FN50), CD107a-PE-Cy5 and APC (H4A3), CCR7-FITC (150503), CD90-BV605 (5E10), CD123-PE and PerCP-Cy5.5 (7G3), and Lineage cocktail 2 (CD3, CD14, CD19, CD20, CD56)-FITC (SK7, MφP9, Sj25G1, L27 and NCAM16.2). $CD19^{-}$and CD37CAR expression were detected by biotinylated goat anti-mouse Fab antibody (Jackson ImmunoResearch) followed by streptavidin-PE (BD Biosciences). CD33CAR expression was detected using CD34-APC (4H11). Flow cytometry was performed on BD FACSCanto or LSR II instruments (BD Biosciences) and analyzed with the FlowJo software

(TreeStar). To study the glycosylation of CD37, AML cell lines have been incubated 30 min, at 37C, with 0.1 U/mL of Neuraminidase in PBS neuraminidase before the staining. For the staining of AML cells, PBMCs and BMMCs, Fcγ receptor blocking was performed using BD Fc Block (BD Biosciences) prior to any relevant staining. Isotype controls were used to set the threshold of background fluorescence for each cell population of interest. Propidium Iodide (PI) was used to discriminate dead cells (Thermo Fisher Scientific). CD37 antigen density was evaluated using the QIFIKIT following the manufacturer's instructions (Dako). A special order of unconjugated anti-CD37 (HH1, Santa-Cruz) antibody and the corresponding isotype were used for the detection in that assay.

Retroviral transduction and expansion of human T cells

Human PBMCs were isolated from healthy donors' buffy coat as described above. Retroviral supernatants were produced as previously described.[32] T cells from PBMCs were first activated before transduction. Briefly, PBMCs were resuspended in X-VIVO 15 medium (Lonza) supplemented with 5% human serum (PAN Biotech) and 100 U/mL recombinant human IL-2 (Clinigen) (complete T cell medium) at $0.5x10^6$ cells/mL. Two milliliters of cells were then transferred per well of 24-well plate, previously coated with 1 μg/mL anti-CD3 and anti-CD28 (OKT3 and CD28.6, Thermo Fisher Scientific) and incubated at 37°C for 48 h. Activated T cells were transferred to a non-tissue culture-treated 24-well plate previously coated with 50 μg/mL retronectin (Takara Bio. Inc.). Retroviral supernatant was added, and the cells were spun down at 750g, 32°C for 1 h. T cells were transduced a second time the next day following the same procedure. On day 3, medium was renewed with complete T cell medium. On day 4, the transduction efficiency was evaluated by flow cytometry and cultures were scaled-up. Thereafter, cells were refreshed every 2 days with complete T cell medium by dilution to $1x10^6$ cells/mL for 7 days. Some experiments were performed with T cells maintained in media supplemented with 30 nM of dasatinib from day 2 to day 11 before being washed and switched back to media without dasatinib. On specific days of the expansion, viability and cell concentration was evaluated using a Countess II Automated Cell Counter (Thermo Fisher Scientific). Expanded T cells were frozen in batches and transferred to liquid nitrogen.

Isoform detection assay

HEK-P cells were seeded at $1.2x10^6$ cells/well of a 6-well plate and co-transfected 24 h later with a vector encoding a single CD37 isoform and a vector encoding GFP. The cells were stained 48 h later using 2 different clones of commercial anti-CD37 antibody (HH1 and M-B371). An aliquot of cells was lysed in RIPA buffer to confirm protein expression in Western blot. Lysates were run in SDS-PAGE with β-mercaptoethanol and transferred onto PVDF membranes (Bio-Rad). Western-blot of CD37 isoforms were detected using an anti-HA tag antibody (Invitrogen, mAb clone 2–2.2.14, 1:5000) or an anti-CD37 antibody (Cell Signaling Technology, mAb clone E4K2M, 1:1000) overnight at 4°C. The primary antibody was detected using a goat anti-rabbit IgG antibody conjugated to horseradish peroxidase (Invitrogen, 31460, 1:2000).

Reporter assay

Jurkat76 cells were first stably transduced with pSIRV-NFAT-eGFP which was a gift from Peter Steinberger (Addgene plasmid # 118031; http://n2t.net/addgene:118031; RRID:Addgene_118031),[45] and a single cell clone was selected by limiting dilution ($J76^{NFAT-GFP}$). The cells were next transduced with different CAR constructs as described above ($CAR\text{-}J76^{NFAT-GFP}$). Target cells were labelled with Cell Trace Violet (Thermo Fisher Scientific) according to the manufacturer's recommendations to distinguish them from effector cells. $CAR\text{-}J76^{NFAT-GFP}$ were cocultured with target cells for 24 h at an effector-to-target (E:T) ratio of 1:2 in complete RPMI medium. The percentage of CAR-expressing population was adjusted with untransduced $J76^{NFAT-GFP}$ cells for each $CAR\text{-}J76^{NFAT-GFP}$. After incubation, the GFP signal was measured by flow cytometry.

Generation of CD37 knock-out U-937cells

U-937 cells were first retrovirally transduced with MSCV_Cas9_puro which was a gift from Christopher Vakoc (Addgene plasmid # 65655; http://n2t.net/addgene:65655; RRID:Addgene_65655)[80] and selected for 48 h with 0.1 μg/mL puromycin (Thermo Fisher Scientific). The cells were then electroporated with a sgRNA targeting at least the 3 main CD37 splice variants (CRISPR1015113_SGM, Invitrogen) using a BTX 830 Square Wave Electroporation System (BTX Technologies). The electroporation was performed with $3x10^6$ U-937-$Cas9^+$ cells and 7.5ug of CD37 sgRNA, in a 1-mm gap cuvette, at 125 V for 2 ms. Immediately after electroporation, cells were transferred to complete RPMI medium and further expanded for 3 days. CD37 knock-out cells ($U\text{-}937^{CD37KO}$) were finally stained with anti-CD37 (HH1) and sorted on the negative population.

Degranulation analysis

Effector T cells and target cells were resuspended in complete RPMI medium and plated at an E:T ratio of 1:2 in a 96-well plate in duplicates. Then, anti-CD107a (H4A3) antibody, GolgiStop and GolgiPlug (all from BD Biosciences) were added, and the plate was incubated at 37°C, 5% CO_2 for 6 h. After incubation, T cells were stained with anti-CD3 (SK7) antibody (BD Biosciences), and flow cytometry was performed.

Cytokine quantification

Effector T cells and target cells were resuspended in complete RPMI medium and plated at an E:T ratio of 1:2 in a 96-well plate in duplicates. For AML primary cells, cells were seeded in plain X-VIVO 15 medium w/o phenol red (Lonza) instead. After 24 h, the cells

were spun down, and the supernatant transferred into another plate and kept at −80°C. Upon thawing, supernatants were diluted 1:3 in plain X-VIVO 15 w/o phenol red supplemented with 2.5% human serum albumin (Octa pharma). Cytokines were quantified using the Bio-Plex Pro Human Cytokine 17-plex assay on a Luminex 200 system (Bio-rad).

Bioluminescence (BLI)-based cytotoxicity assay

The killing assay was performed as reported.[32] Briefly, luciferase-expressing target cells (GFP-Luc$^+$) were mixed with 75 μg/mL Xenolight D-Luciferin potassium salt (PerkinElmer) and seeded in 96-well white plates in triplicates. For some assay, target cells have been treated in with 0.1 U/mL of Neuraminidase in PBS, for 30 min at 37°C. Then the cells were washed twice before adding D-Luciferin potassium salt. Effector T cells were added at indicated E:T ratios and incubated at 37°C, 5% CO_2. BLI was measured with a luminometer (PerkinElmer) as relative light units (RLU). Triplicate wells were averaged, and lysis percentage was calculated using the following equation: % specific killing = 100 x (spontaneous cell death RLU – sample RLU)/(spontaneous death RLU – maximal killing RLU).

Cytometry-based cytotoxicity assay

Healthy PBMCs and AML BMMCs used as target cells were first labelled with Cell Trace Violet (Thermo Fisher Scientific) following the manufacturer's instructions. Effector T cells and target cells were resuspended in plain X-VIVO 15 medium and plated at an E:T ratio of 2:1 (5:1 against AML BMMCs) in a 96-well plate, in duplicates. After 24 h of incubation, cells were collected, and the plate was treated with Accutase (Thermo Fisher Scientific) for 3 min at 37°C to release adherent cells. PBMCs were subsequently stained for lineage markers while anti-CD3, -CD4, -CD34, -CD38 and -CD45 antibodies were used for AML BMMCs. Counting beads (Thermo Fisher Scientific) were added to quantify the absolute count of cells. PI was added extemporary before flow acquisition to gate out dead cells. For monocyte cytoxicity assays, monocytes were magnetically isolated from healthy PBMCs using the Pan Monocyte Isolation Kit human (Miltenyi Biotec) according to the manufacturer's instructions. The sorted cells were then stained for lineage markers with anti-CD3, -CD11b, -CD14, -CD16, and CD20 antibodies to confirm the population. Then, monocytes were labeled with CellTrace CFSE (Thermo Fisher Scientific) following the manufacturer's instructions, before being co-cultured with Mock and CD19$^-$, CD37 T cells at 37C, for 12 to 15h. Effector T cells and target monocyte, from the same healthy donor, were resuspended in regular X-VIVO 15 medium and plated at a 2:1 E:T ratio in ultra-low attachment 96-well plates. After incubation, cells were harvested and labeled with anti-CD33, -CD3, -CD11b, -CD14, -CD20 antibodies. Counting beads were added to quantify the absolute count of cells. PI was added extemporary before flow acquisition to gate out dead cells.

Colony-forming unit (CFU) assay

BMMCs were thawed the day of the assay and an aliquot of cells was left untreated for 6 h at 37°C, 5% CO_2. Effector T cells were co-cultured with autologous BMMCs for 6 h in complete RPMI medium at an E:T ratio of 10:1. After incubation, cells were washed and adjusted to 2.5x10^5 alive BM cells/mL in a final volume of 300 μL. For this purpose, the aliquot of untreated BMMCs was used to estimate the true viability count upon thawing (no effectors). The solution was then homogenized with 4 mL of complete MethoCult medium (STEMCELL), and 1.1 mL was transferred per well of 6-w plate (in triplicate). After 10 days, Colony-forming unit-erythroid (CFU-E), Burst-forming unit-erythroid (BFU-E) and colony-forming unit-granulocyte, macrophage (CFU-GM) colonies were counted under a microscope using a background grid.

QUANTIFICATION AND STATISTICAL ANALYSIS

All experimental data are represented as individual values with the mean or the mean ± SD. Comparisons between two groups were assessed by two-tailed paired Student's t-test. Statistical differences among three or more groups were evaluated with One-way ANOVA corrected with post-hoc tests. two-way ANOVA corrected with post-hoc tests was used to assess differences between two variables among three or more groups. Survival studies were assessed by Kaplan–Meier curves and the log rank (Mantel-Cox) test. Data were analyzed with Prism 9 software (GraphPad Software). p values lower than 0.05 were considered statistically significant and written as follow: * = $p < 0.05$, ** = $p < 0.01$, *** = $p < 0.001$, **** = $p < 0.0001$.

Cell Reports Medicine, Volume *5*

Supplemental information

CD37 is a safe chimeric antigen receptor target to treat acute myeloid leukemia

Benjamin Caulier, Sandy Joaquina, Pascal Gelebart, Tara Helén Dowling, Fatemeh Kaveh, Moritz Thomas, Luka Tandaric, Patrik Wernhoff, Niveditha Umesh Katyayini, Cara Wogsland, May Eriksen Gjerstad, Yngvar Fløisand, Gunnar Kvalheim, Carsten Marr, Sebastian Kobold, Jorrit M. Enserink, Bjørn Tore Gjertsen, Emmet McCormack, Else Marit Inderberg, and Sébastien Wälchli

Figure S1. Staining with anti-CD37 antibodies

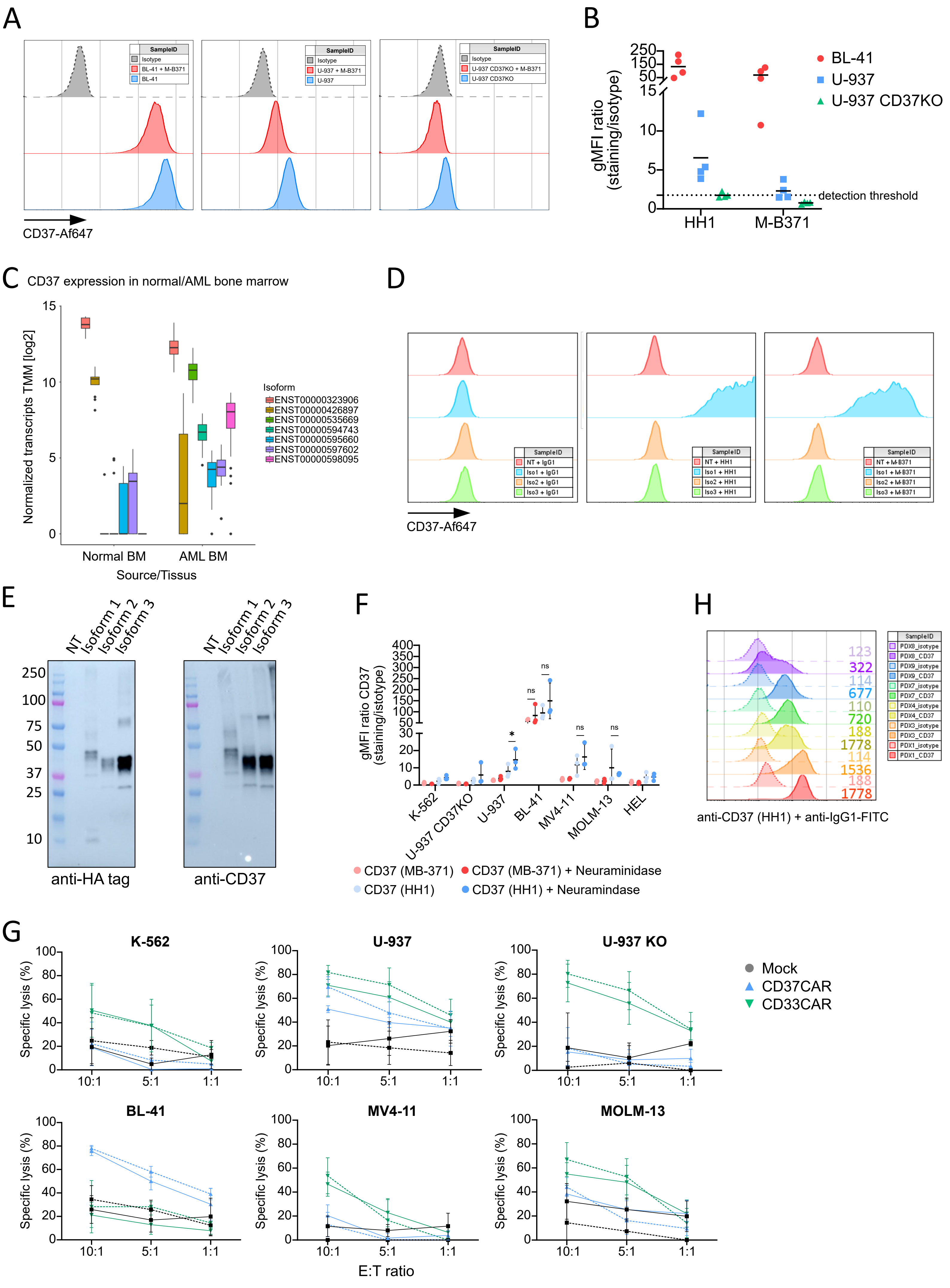

Figure S1. Staining with anti-CD37 antibodies. Related to Figure 1. (A) Representative detection of CD37 on B cell lymphoma (BL-41) and AML cells (U-937 WT and KO for CD37) using 2 different clones of commercial anti-CD37 antibody (M-B371 [red histograms] and HH1 [blue histograms], both murine monoclonal IgG1 used at 1 µg per 1x106 cells). A murine IgG1 isotype was used to set the negative threshold and the percentage of positive cells is displayed. (B) Relative quantification of CD37 staining in (A). The geometric Mean Fluorescence Intensity (gMFI) of CD37 staining was divided by the background staining of an isotype control for each cell line (n = 4 experiments). Bars represente the mean. (C) Comparison of CD37 isoform expression between normal bone marrows vs AML bone marrows from the European Genome-Phenome Archive (EGA)68. Normalized AML datasets are from EGAD00001004187, DAC: EGAC00001000956, 75 samples of AML bone marrows and 13 normal bone marrows were analysis. Differences in transcript expression were tested with Kruskal-Wallis test in R and found significant difference for all transcripts. ENST00000323906 p-value = 0.0002991, ENST00000426897 p-value = 1.484e-08, ENST00000598095 p-value = 1.446e-08, ENST00000535669 p-value = 9.464e-09, ENST00000594743 p-value = 9.608e-09, ENST00000595660 p-value = 1.797e-06, ENST00000597602 p-value = 2.794e-05. (D) We tested if the discrepancy in CD37 detection on AML cells between the M-B371 and HH1 antibody clones was due to the specific recognition of one of the three main CD37 isoforms. We observed that both antibodies solely reacted against the isoform-1 of CD37. Representative detection of CD37 isoforms (isoforms 1, 2 and 3) transiently expressed in HEK cells. Cells were co-transfected with a vector encoding a single CD37 isoform and a vector encoding GFP. GFP-positive, Propidium Iodide (PI)-negative HEK cells were stained 48 hours post-transfection for CD37 using 2 different clones of commercial anti-CD37 antibody (HH1 and M-B371). Inserts represent other channel overlays. NT = non-transfected. (n = 1 representative experiment over 2). (E) Western-blot analysis of lysates from (D). CD37 isoforms were detected using an anti-HA tag antibody or an anti-CD37 antibody. The primary antibody was detected using a goat anti-rabbit IgG antibody conjugated to horseradish peroxydase. NT = non-transfected. (F) Relative quantification of CD37 staining on different AML cell lines (U-937, U-937CD37KO, MV4-11, MOLM-13, HEL) treated or not with Neuraminidase. BL-41 (CD37high) and K-562 (CD37null) cell lines have been used as control. The geometric Mean Fluorescence Intensity (gMFI) of CD37 staining was divided by the background staining of an isotype control for each cell line (n = 3 experiments, mean ± SD). AML cell lines treated with neuraminidase showed a trend towards increased detection of the HH1 epitope at the membrane, not a decrease. HH1 does not appear to depend on sialylation. Two-way ANOVA followed by Tukey's multiple comparisons tests. (G) Specific cytotoxicity of T cells incubated 8 hours with target cells (K-562, BL-41, U-937, U-937 CD37KO, MV4-11 and MOLM-13) treated or not with Neuraminidase. E:T ratios indicated. (n = 3 donors, mean ± SD). No significant difference was observed in the killing of AML cell lines either treated or not with neuramindase. Two-way ANOVA followed by Tukey's multiple comparisons tests. No significant difference was observed. (H) Flow cytometry-based, surface CD37 protein quantification of AML-PDX cells (n = 6).
The murine IgG1 anti-CD37 mAb clone HH1 was used for the detection. A murine IgG1 isotype was used to set the background. The numbers indicate the Mean Fluorescence Intensity (MFI).

Figure S2. Detection of CD37 in a small cohort of patients

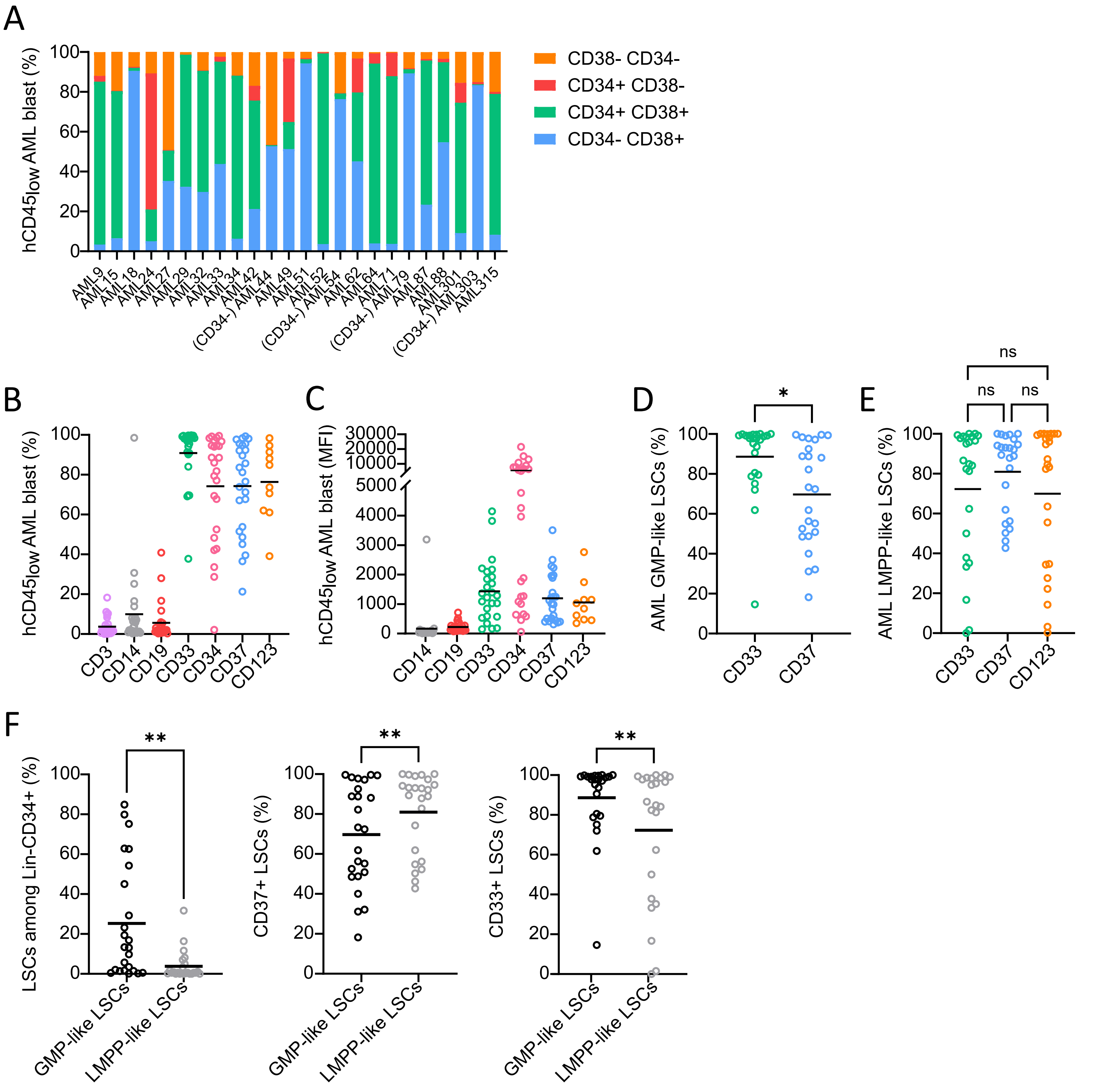

Figure S2. Detection of CD37 in a small cohort of patients. Related to Figure 1. (A) Percentage of CD34/CD38 double-positive cells among the hCD45low blast population of AML BM samples (n=25). Percentage (B) and geometric median fluorescent intensity (C) of CD3, CD14, CD19, CD33, CD34, CD37 (mAb clone HH1) and CD123 surface proteins among the hCD45low blast population of primary AML BM samples (n = 25, mean). A murine IgG isotype was used to set the background for each antibody. (C) Percentage of CD14, CD19, CD33, CD37 and CD123 surface proteins on hCD45low CD34+ AML blast population from primary BM samples (n = 25, mean). One-way ANOVA followed by Dunnett's multiple comparison tests. (D) Percentage of CD33 and CD37 on the granulocyte-macrophage progenitors (GMP)-like LSC population of primary AML BM samples (n = 24, mean). The anti-CD37 mAb clone HH1 was used for detection. A murine IgG1 isotype was used to set the background. GMP-like LSCs were defined as Lin- CD34+ CD38+ CD123+ CD45RA+. Paired t-test (two-tailed) was used for statistical analysis.
(E) Percentage of CD33, CD37 and CD123 among the lymphoid-primed multipotential progenitors (LMPP)-like LSC population of primary AML BM samples (n = 24, mean). The anti-CD37 mAb clone HH1 was used for detection. A murine IgG1 isotype was used to set the background. LMPP-like LSCs were defined as Lin-CD34+ CD38- CD90- CD45RA+. One-way ANOVA followed by Tukey's multiple comparison tests.
(F) (Left) Percentage of granulocyte-macrophage progenitors (GMP)- and lymphoid-primed multipotential progenitors (LMPP)-like leukemic stem cells (LSCs) in primary AML BM samples (n = 25). GMP-like LSCs were defined as Lin- CD34+ CD38+ CD123+ CD45RA+ and LMPP-like LSCs as Lin- CD34+ CD38- CD90- CD45RA+. t-test (paired) was used for statistical analysis. Percentage of CD37 (center) and CD33 (right) on GMP- and LMPP-like LSCs in primary AML BM samples (n = 25). t-test (paired) was used for statistical analysis. Bars represent the mean.

Figure S3. Clustering of AML samples in a large cohort

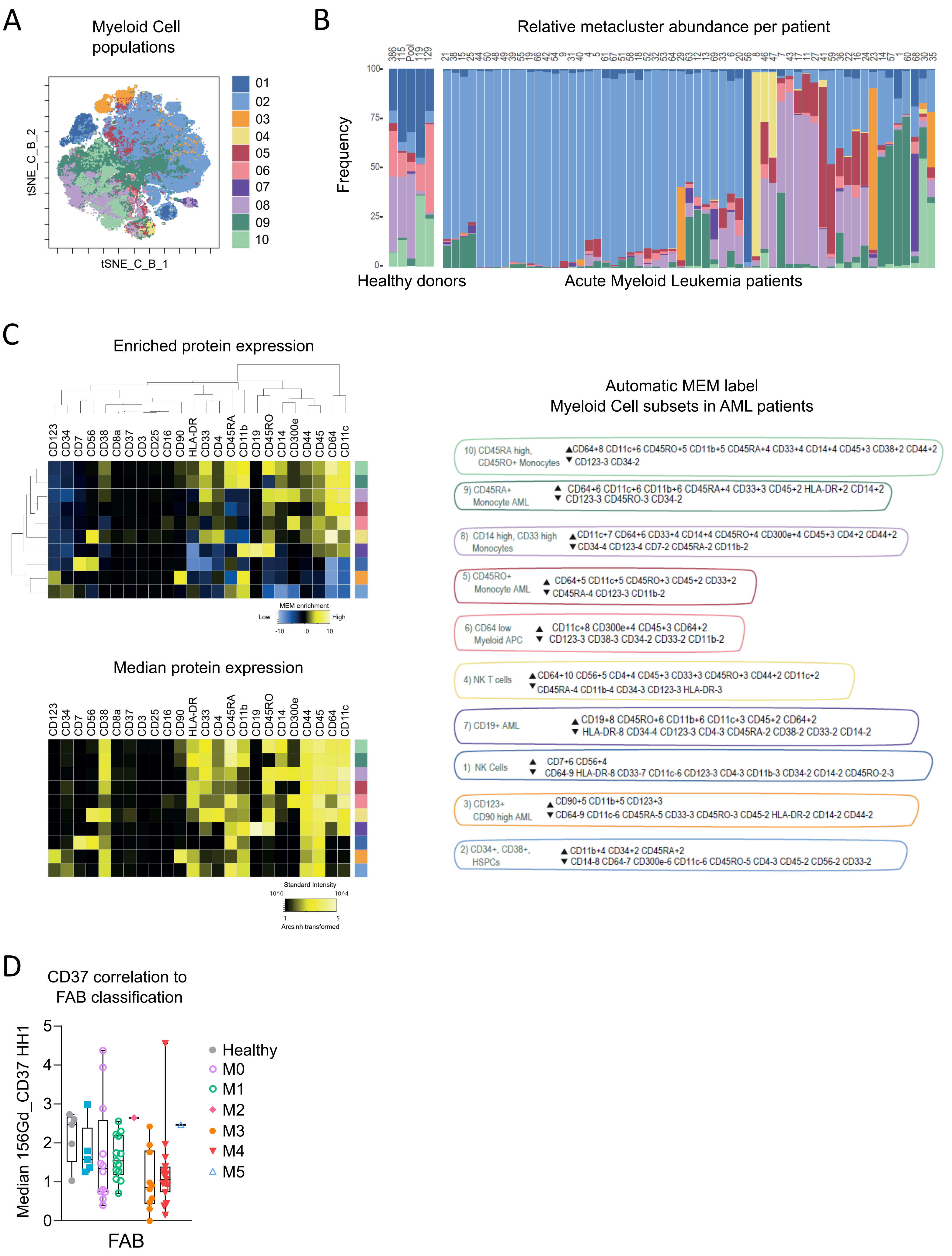

Figure S3. Clustering of AML samples in a large cohort. Related to Figure 1. (A) A tSNE-Cuda of myeloid cells from a concatenated AML sample of 2 286 000 cells (n=59 AML patients) was coloured by metaclusters highlighting position and size of meta cluster. (B) Relative meta cluster abundance as percent of total population in healthy donors to the left (n= 5) and AML patients (n= 59) ordered according to similar meta cluster distribution. (C) Marker expression modelling (MEM) characterizing cell subsets of FlowSOM unsupervised clusters. The MEM labels were computationally assigned using the 9 other metaclusters as reference points. The heatmaps depict the protein enrichment values used to generate the MEM labels and the median protein expression was given for each myeloid cell subset. (D) The distribution of patients in the French American British morphological stratification system did not correlate to CD37 expression. We did not find a significant increase of CD37 expression in FAB M5 as reported in other studies.

Figure S4. Metacluster analysis

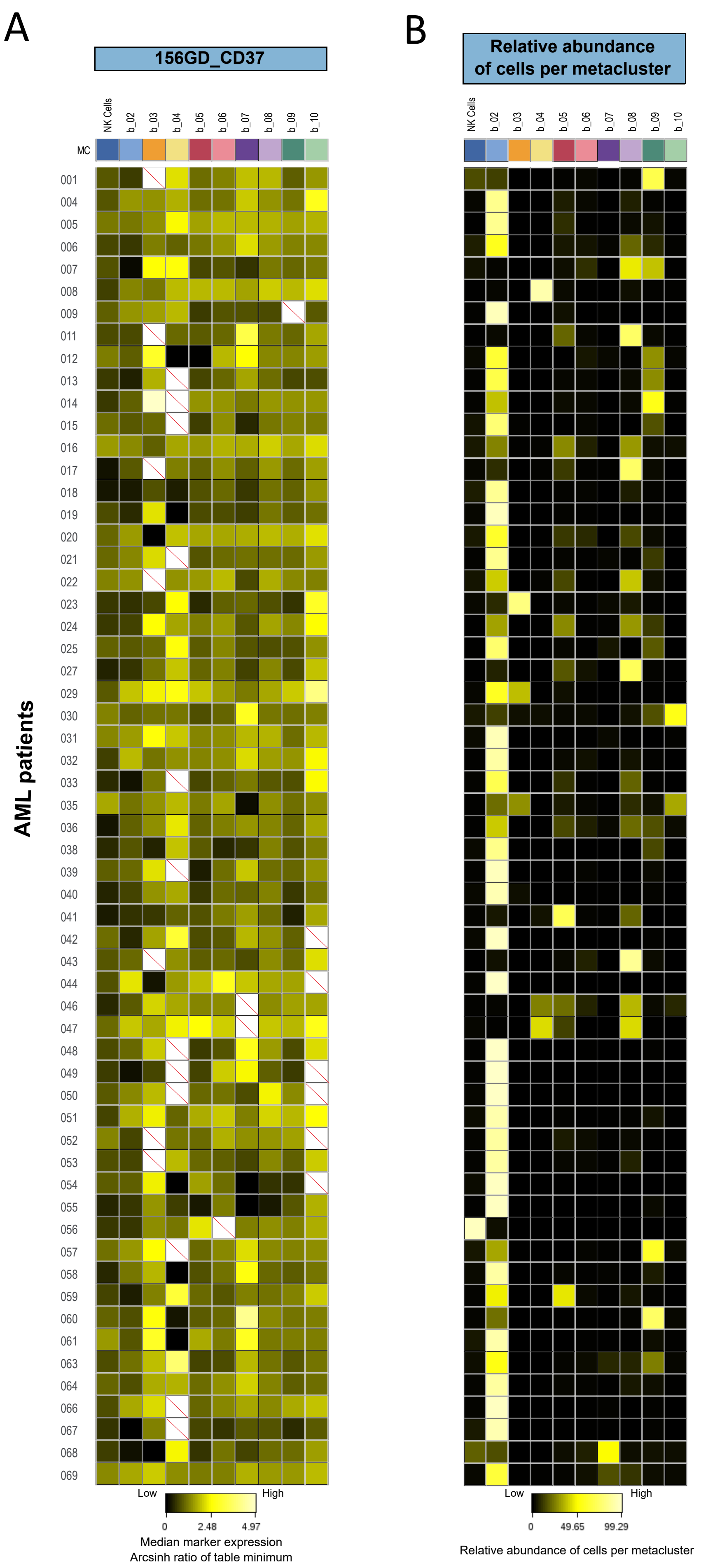

Figure S4. Metacluster analysis. Related to Figure 1. (A) Heatmap of CD37 expression according to meta cluster in each patient shown as median marker expression of arcsinh ratio of tables minimum (n=59). The highest expression levels in patients were observed in MC 3, 4, 7 and 10. (B) Heatmap showing relative meta cluster abundance as percent of total population (n=59). Most patients have the highest frequency of their cells in meta cluster 2.

Figure S5. CD37 mRNA analysis

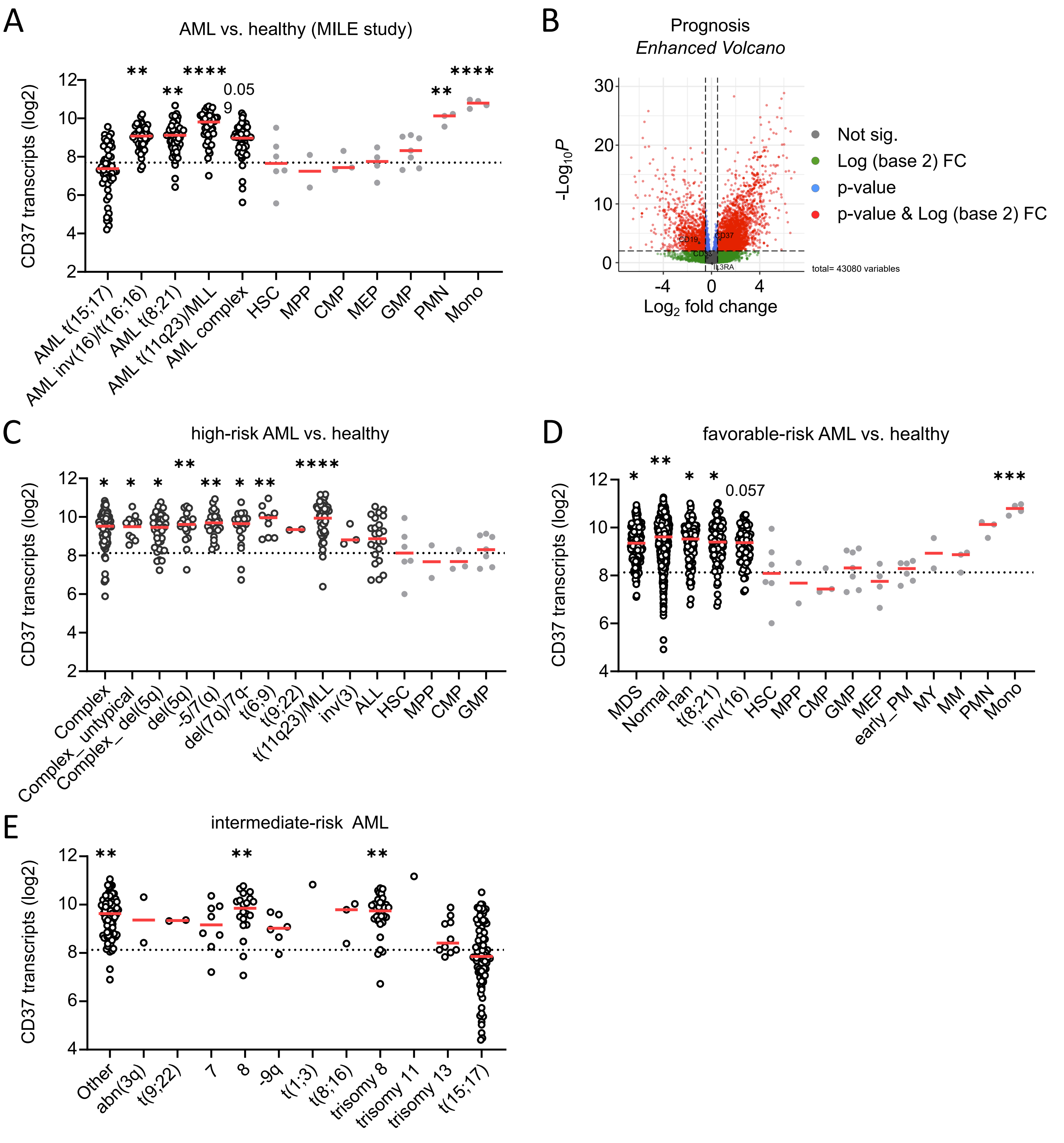

Figure S5. CD37 mRNA analysis. Related to Figure 1. (A) CD37 gene expression analysis of high-risk AML downloaded from BloodSpot67 (n = 242). Curated and normalized AML microarray datasets (black empty circles) are from GSE13159, GSE15434, GSE61804, GSE14468, TCGA-LAML and human healthy hematopoietic cells (filled grey circles) are from GSE42519 (n = 18). The dotted line sets the averaged CD37 expression on HSC. ALL = acute lymphoid leukaemia, HSC = hematopoietic stem cells, MPP = multipotential progenitors, CMP = common myeloid progenitors and GMP = granulocyte-macrophage progenitors. AML abbreviations are defined in Supplementary Table 3. One-way ANOVA tests followed by Dunnett’s multiple comparisons versus healthy HSCs. Non-significant statistical tests are not displayed.
(B) Volcano-plot showing the expression of significantly changed mRNAs with FDR < 0.05 and log2FC (fold change) ≥ 0.5. The red-marked dots represent up-regulated genes, the green ones represent down-regulated genes, and the grey ones show no significance. CD37 gene expression analysis of (C) (high-risk), (D) (favourable-risk) and (E) (intermediate-risk) AML downloaded from BloodSpot67 (n = 2074). Curated and normalized AML microarray datasets (black empty circles) are from GSE13159, GSE15434, GSE61804, GSE14468, TCGA-LAML and human healthy hematopoietic cells (filled grey circles) are from GSE42519. The dotted line sets the averaged CD37 expression on HSC. AML and healthy cell abbreviations are defined in Supplementary Table 3. One-way ANOVA tests followed by Dunnett’s multiple comparisons versus healthy HSCs. Non-significant statistical tests are not displayed.

Figure S6. Single cell transcriptomic data

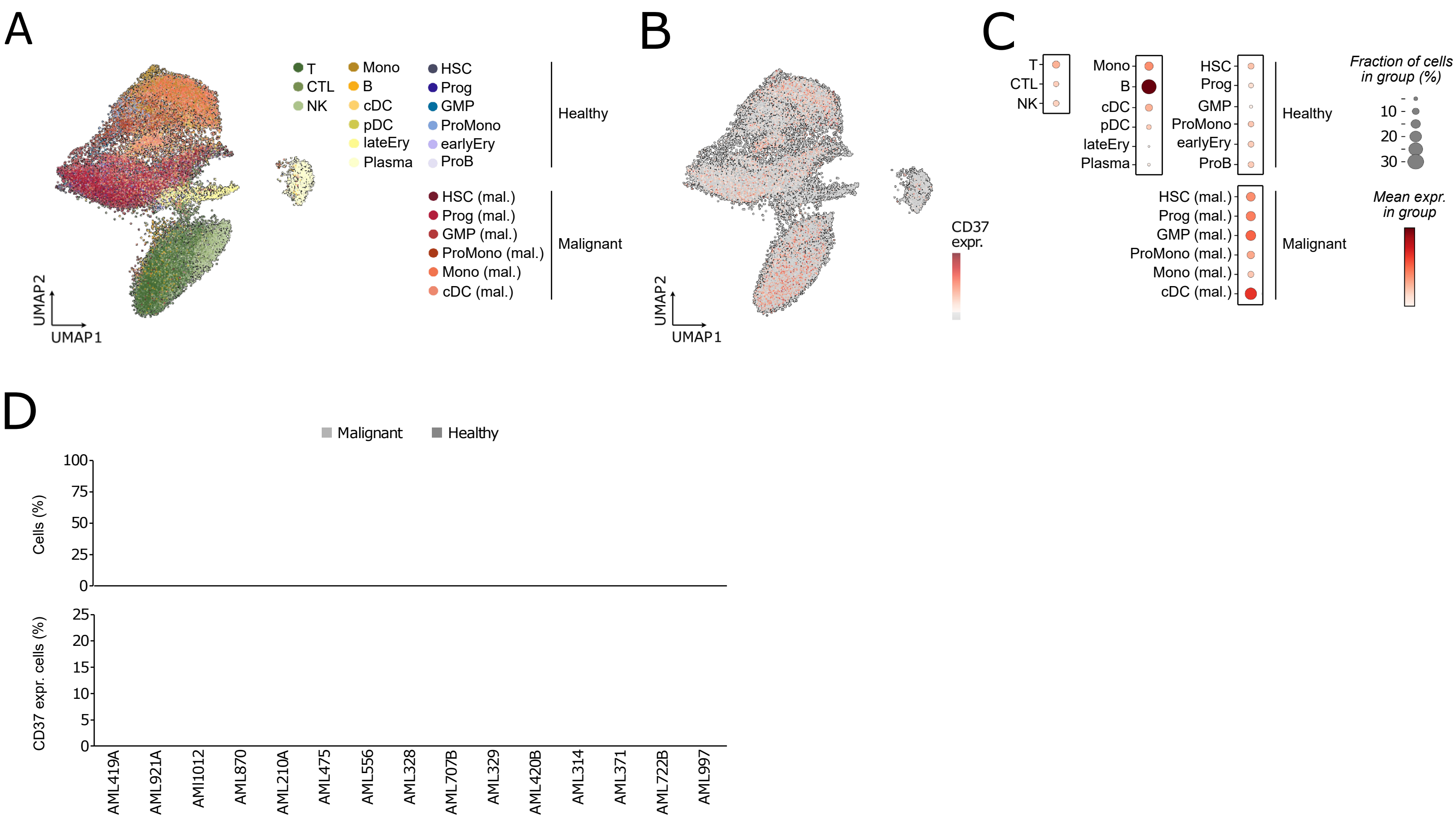

Figure S6. Single cell transcriptomic data. Related to Figure 1. (A) UMAP showing 28,404 healthy and malignant cells from 15 individuals suffering from AML with different mutations1. Colours highlight the 15 different healthy and 6 different malignant cell types. (B) Expression of CD37 in healthy and malignant cells. Normalized gene expression values were log transformed and visualized in a UMAP embedding. (C) Dotplot of CD37 expression on healthy and malignant cell types. Dot size indicates the fraction of cells expressing CD37; colour intensity shows mean normalized gene expression per cell type. (D) Top: amount of malignant and normal cells per AML patient. Bottom: Percentage of malignant and normal cells expressing CD37 for each AML patient.

Figure S7. Constructs

CD19CAR

METDTLLLWVLLLWVPGSTGDIQMTQTTSSLSASLGDRVTISCRASQDISKYLNWYQQKPDGTVKLLIYHTSRLHSGV
PSRFSGSGSGTDYSLTISNLEQEDIATYFCQQGNTLPYTFGGGTKLEITKAGGGGSGGGGSGGGGSGGGGSEVKLQES
GPGLVAPSQSLSVTCTVSGVSLPDYGVSWIRQPPRKGLEWLGVIWGSETTYYNSALKSRLTIIKDNSKSQVFLKMNSL
QTDDTAIYYCAKHYYYGGSYAMDYWGQGTSVTVSSDFVPVFLPAKPTTTPAPRPPTPAPTIASQPLSLRPEACRPAAG
GAVHTRGLDFACDIYIWAPLAGTCGVLLLSLVITLYCNHRNRFSVVKRGRKKLLYIFKQPFMRPVQTTQEEDGCSCRF
PEEEEGGCELRVKFSRSADAPAYQQGQNQLYNELNLGRREEYDVLDKRRGRDPEMGGKPRRKNPQEGLYNELQKDKMA
EAYSEIGMKGERRRGKGHDGLYQGLSTATKDTYDALHMQALPPR

CD37CAR

METDTLLLWVLLLWVPGSTGDIVMTQSHKLLSTSVGDRVSITCKASQDVSTAVDWYQQKPGQSPKLLINWASTRHTGV
PDRFTGSGSGTDYTLTISSMQAEDLALYYCRQHYSTPFTFGSGTKLEIKGGGGSGGGGSGGGGSGGGGSEIQLQQSGP
ELVKPGASVKVSCKASGYSFTDYNMYWVKQSHGKSLEWIGYIDPYNGDTTYNQKFKGKATLTVDKSSSTAFIHLNSLT
SEDSAVYYCARSPYGHYAMDYWGQGTSVTVSSDFVPVFLPAKPTTTPAPRPPTPAPTIASQPLSLRPEACRPAAGGAV
HTRGLDFACDIYIWAPLAGTCGVLLLSLVITLYCNHRNRFSVVKRGRKKLLYIFKQPFMRPVQTTQEEDGCSCRFPEE
EEGGCELRVKFSRSADAPAYQQGQNQLYNELNLGRREEYDVLDKRRGRDPEMGGKPRRKNPQEGLYNELQKDKMAEAY
SEIGMKGERRRGKGHDGLYQGLSTATKDTYDALHMQALPPR

CD33CAR

METDTLLLWVLLLWVPGSTGDIQLTQSPSTLSASVGDRVTITCRASESLDNYGIRFLTWFQQKPGKAPKLLMYAASNQ
GSGVPSRFSGSGSGTEFTLTISSLQPDDFATYYCQQTKEVPWSFGQGTKVEVKRTVAGGGGSGGGGSGGGGSGGGGSE
VQLVQSGAEVKKPGSSVKVSCKASGYTITDSNIHWVRQAPGQSLEWIGYIYPYNGGTDYNQKFKNRATLTVDNPTNTA
YMELSSLRSEDTAFYYCVNGNPWLAYWGQGTLVTVSSFVPVFLPAKPTTTPAPRPPTPAPTIASQPLSLRPEACRPAA
GGAVHTRGLDFACDIYIWAPLAGTCGVLLLSLVITLYCNHRNRFSVVKRGRKKLLYIFKQPFMRPVQTTQEEDGCSCR
FPEEEEGGCELRVKFSRSADAPAYQQGQNQLYNELNLGRREEYDVLDKRRGRDPEMGGKPRRKNPQEGLYNELQKDKM
AEAYSEIGMKGERRRGKGHDGLYQGLSTATKDTYDALHMQALPPREFGSGEGRGSLLTCGDVEENPGPPRGWTALCLL
SLLPSGFMSLDNNGTATPELPTQGTFSNVSTNVSYQETTTPSTLGSTSLHPVSQHGNEATTNITETTVKFTSTSVITS
VYGNTNSSVQSQTSVISTVFTTPANVSTPETTLKPSLSPGNVSDLSTTSTSLATSPTKPYTSSSPILSDIKAEIKCSG
IREVKLTQGICLEQNKTSSCAEFKKDRGEGLARVLCGEEQADADAGAQVCSLLLAQSEVRPQCLLLVLANRTEISSKL
QLMKKHQSDLKKLGILDFTEQDVASHQSYSQKTLIALVTSGALLAVLGITGYFLMNRRSWSPTGERLELEP

Single chain variable fragment (V_L-V_H) CD8-hinge-TM 4-1BB CD3z 2A-CD34t

Figure S7. Constructs. Related to Figure 2. Protein sequence of CD19-, CD37- and CD33CAR used in this study.

Figure S8. CD37CAR activity

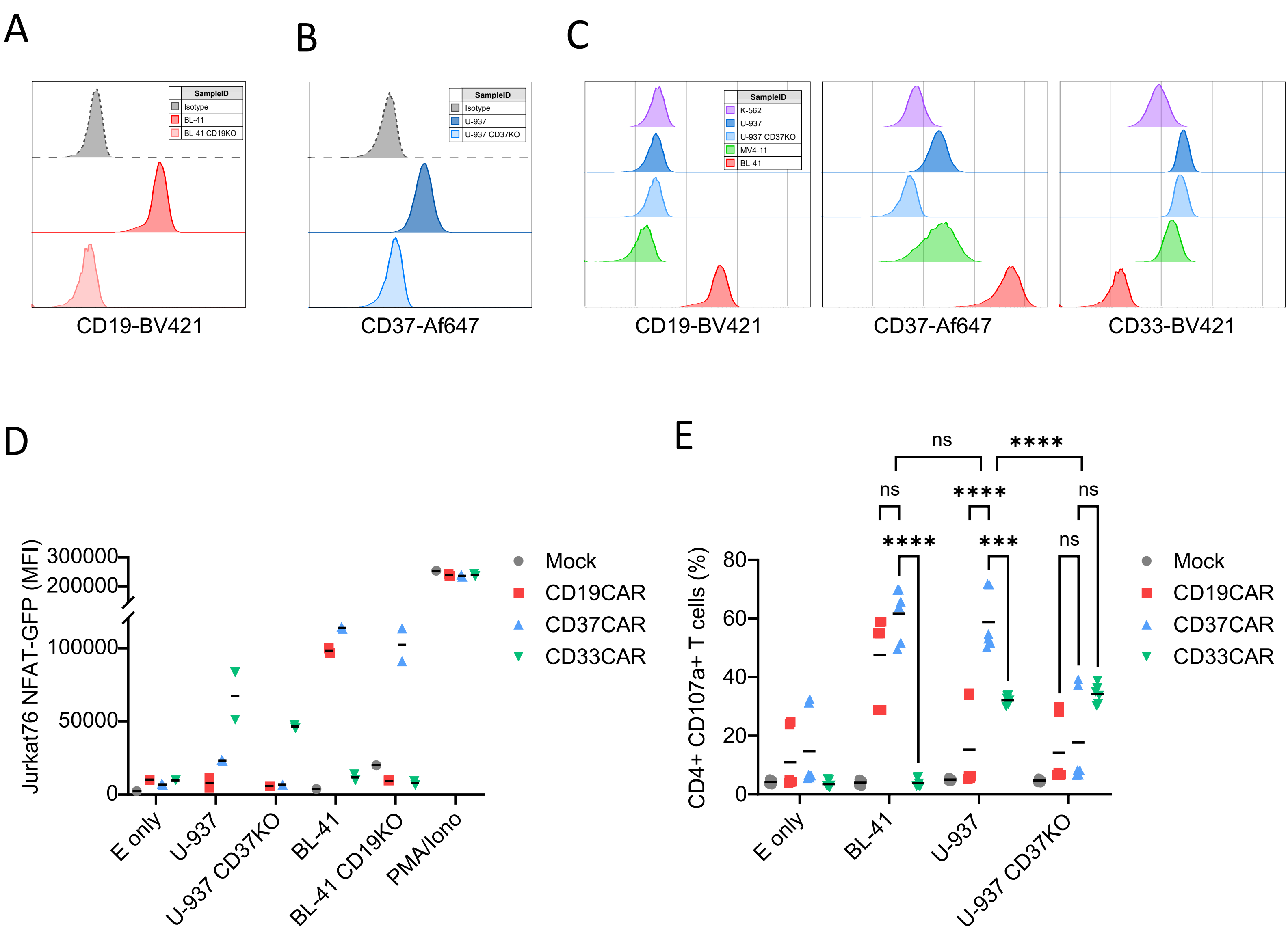

Figure S8. CD37CAR activity. Related to Figure 2. (A) Representative detection of CD19 on BL-41 and BL-41 CD19 knock-out (KO). The dotted line represents the corresponding isotype control. (B) Representative detection of CD37 on U-937 and U-937 CD37KO. The anti-CD37 mAb clone HH1 was used for detection. The dotted line represents the corresponding isotype control. (C) Comparative detection of CD19, CD37 and CD33 on K-562, U-937, U-937 CD37KO, MOLM-13, MV4-11, and BL-41. The anti-CD37 mAb clone HH1 was used for detection. Representative detection of geometric median fluorescent intensity of GFP (D) (NFAT-GFP activation signal) of Jurkat76 cells transduced with Mock, CD19-, CD37- or CD33CAR and co-cultured for 24 hours with the indicated cell lines or left alone (E only). E:T = 1:2 (2 independent experiments, mean). Percentage (E) of CD4+ CD107a+ T cells upon 6 hours of co-culture with the indicated cell lines or left alone (E only). E:T = 1:2 (n = 3 donors in duplicates, mean). Two-way ANOVA followed by Tukey's multiple comparison tests. Comparisons versus CD37CAR are displayed.

Figure S9. Cytokine release

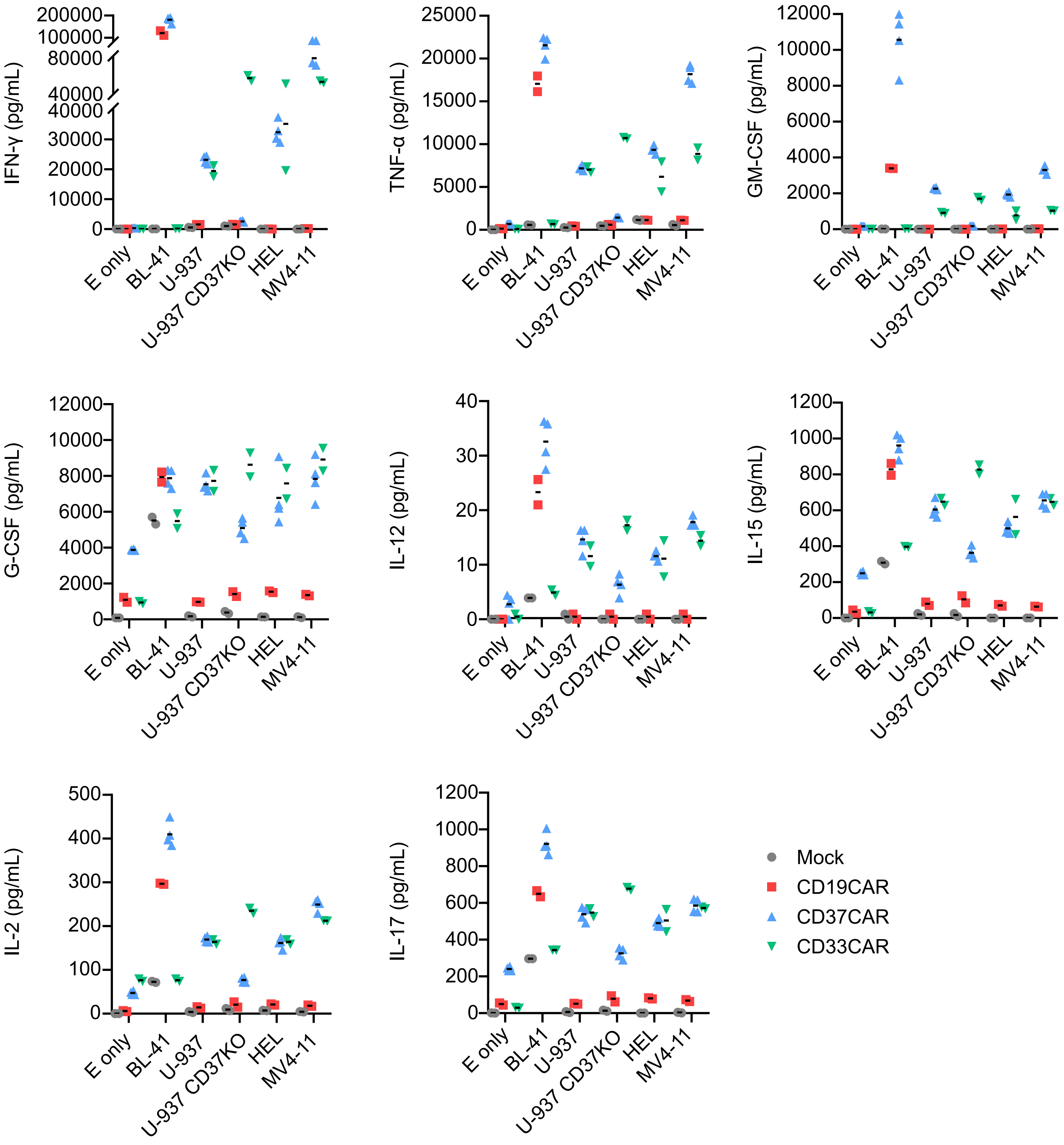

Figure S9. Cytokine release. Related to Figure 2. Secretion (pg/mL) of IFN-γ, TNF-α, GM-CSF, G-CSF, IL-2, IL-12, IL-15, and IL-17 in the supernatant of T cell co-culture with the indicated cell lines or left alone (E only) after 24 hours. E:T = 1:2 (n = 2 donors except CD37CAR n = 4, mean).

Figure S10. Weight of mice

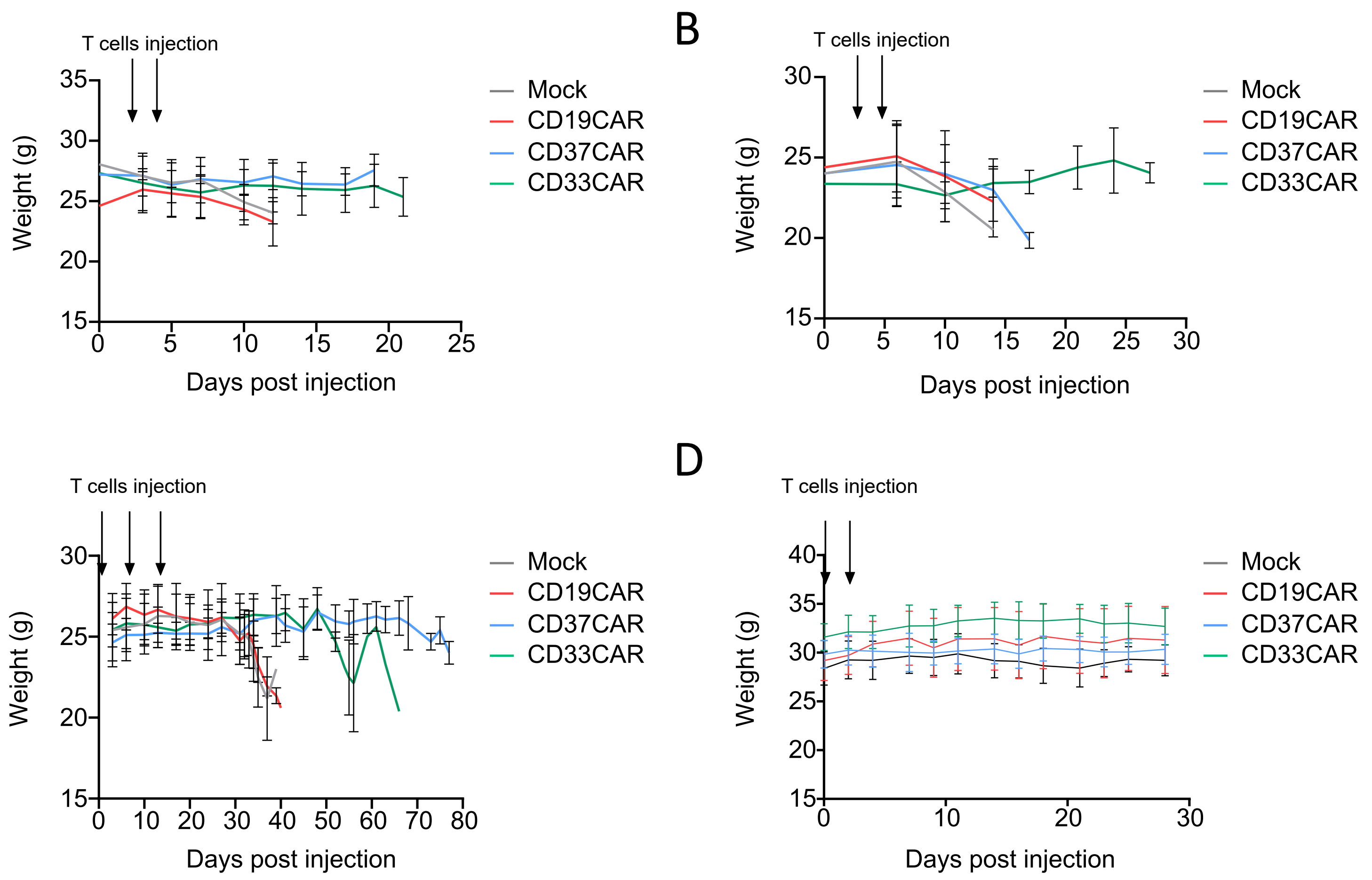

Figure S10. Weight of mice. Related to Figures 4 and 5. (A) Representation of the weight monitoring of the U-937 mice model (Figure 4A-D). (n = 5, mean ± SD). (B) Representation of the weight monitoring of the MOLM-13 mice model (Figure 4E-H). (n = 5, mean ± SD).
(C) Representation of the weight monitoring of the AML-PDX mice model (Figure 5). (n = 7, mean ± SD). (D) Representation of the weight monitoring of the AML-PDX mice model (Figure S11 C-G). (n = 8, mean ± SD).

Figure S11. In vivo models

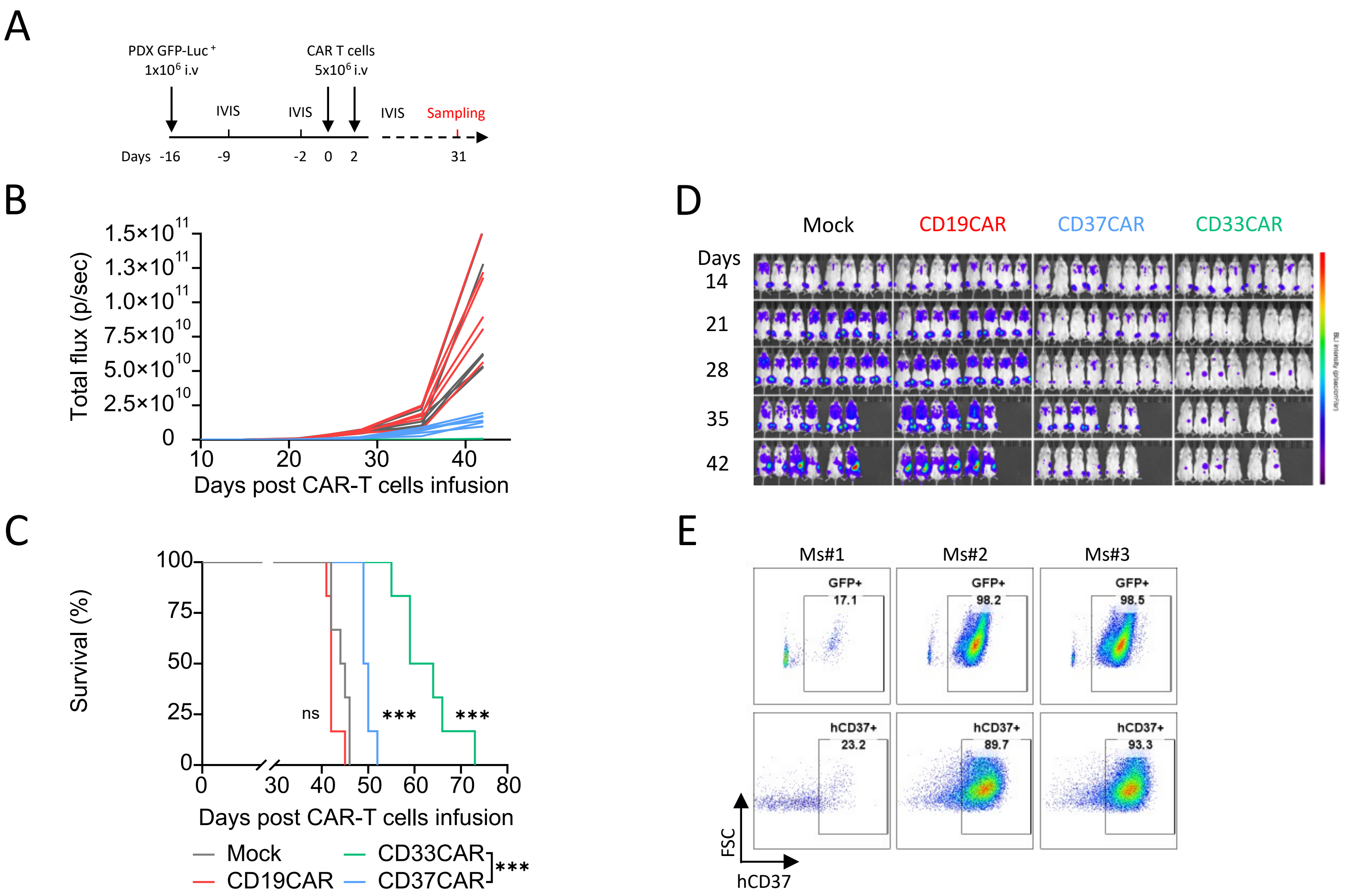

Figure S11. In vivo model. Related to Figure 5. (A) Schematic representation of the 1st PDX in vivo experiment. 1x106 AML-PDX GFP-Luc+ cells were inoculated intravenously (i.v) in NSG mice 16 days prior T cell injection. IVIS was performed 2 days before T cell injection to confirm tumour establishment and randomize the mice. 5x106 Mock, CD19-, CD37- or CD33CAR T cells were injected i.v on day 0 and 2. The % of CAR -expressing population was adjusted between the groups to 60% using Mock cells. Tumor growth was tracked weekly using IVIS. 3 mice were sampled per group on day 31 to analyze recurrent AML cells. (B) Bioluminescence kinetics of the AML-PDX GFP-Luc+ cells growth in NSG mice treated with CAR T cells (n = 8 mice per group). (C) Kaplan-Meier survival curves of NSG mice-bearing AML-PDX GFP-Luc+ cells and treated with CAR T cells (n = 8 mice per group). Comparisons of survival curves were determined by log-rank test.(D) Representative bioluminescence images of (B) and (C). (E) Detection of CD37 (mAb clone HH1) on AML-PDX GFP-Luc+ cells sampled from the bone marrow on day 31 (n = 3 mice of CD37CAR group).

Figure S12. CD37CAR T cells treatment with dasatinib

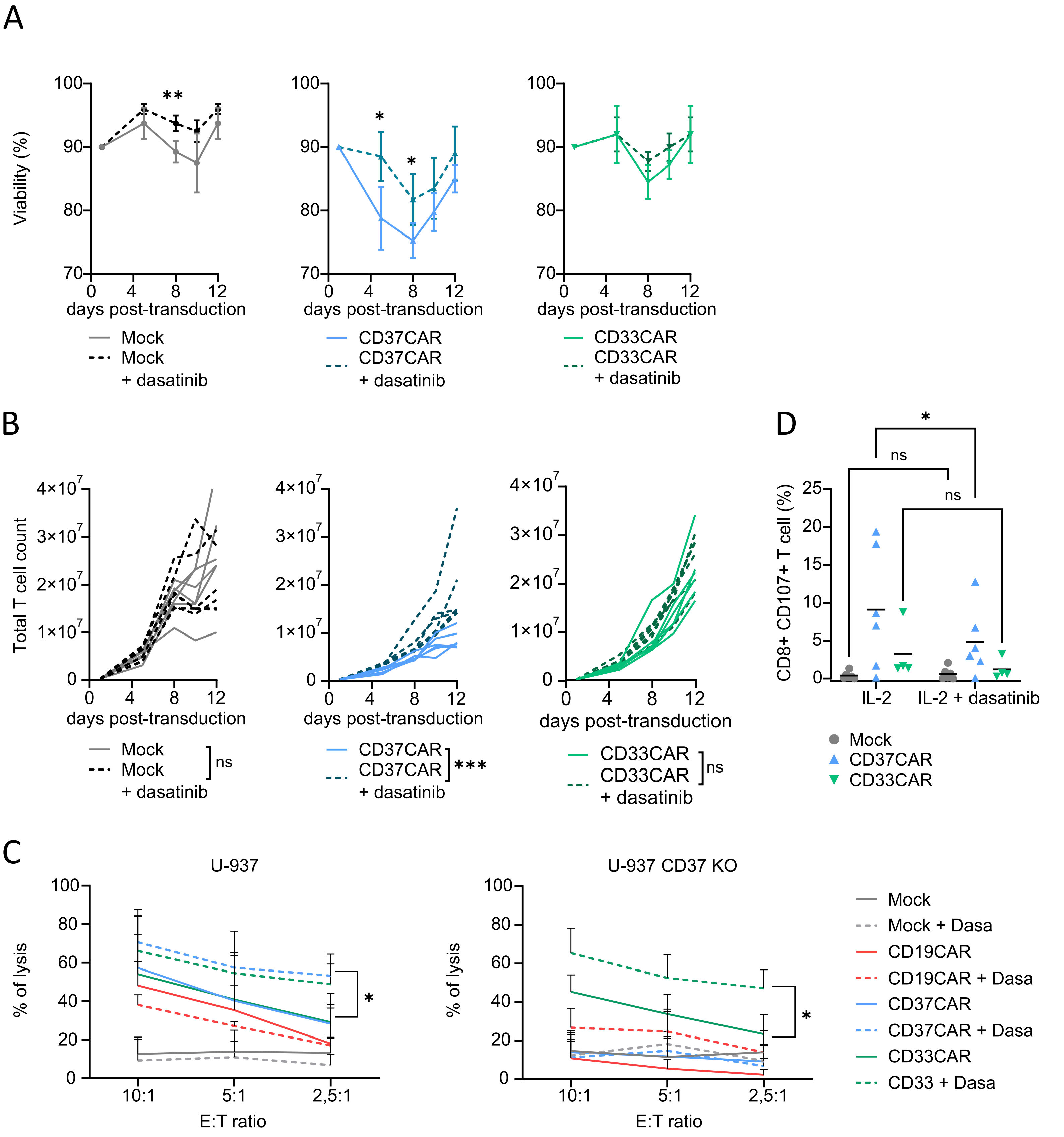

Figure S12. CD37CAR T cells treatment with dasatinib. Related to Figure 5. Viability (A) and expansion in total T cell count (B) of T cell donors-bearing the CAR constructs with or without dasatinib for 12 days post-transduction. (A) Paired t-test was used for statistical analysis (n = 4 donors, mean ± SD). (B) Two-way ANOVA followed by Tukey's multiple comparisons tests (n = 6 donors). Stats are displayed for day 12, as result of the expansion. (C) Specific cytotoxicity of CAR constructs (manufactured either with or without dasatinib for 12 days post-transduction) incubated 6 hours with U-937 and U-937 CD37KO. E:T ratios indicated (n = 6 donors,mean ± SD). Two-tailed paired Student's t-test.
(D) Percentage of CD8+ CD107a+ T cells at 10 days of expansion with or without dasatinib (n = 6 donors, mean). Two-way ANOVA followed by Sifak's multiple comparisons tests.

Figure S13. The resurgence of AML cells in the PDX animal is not due to antigen escape

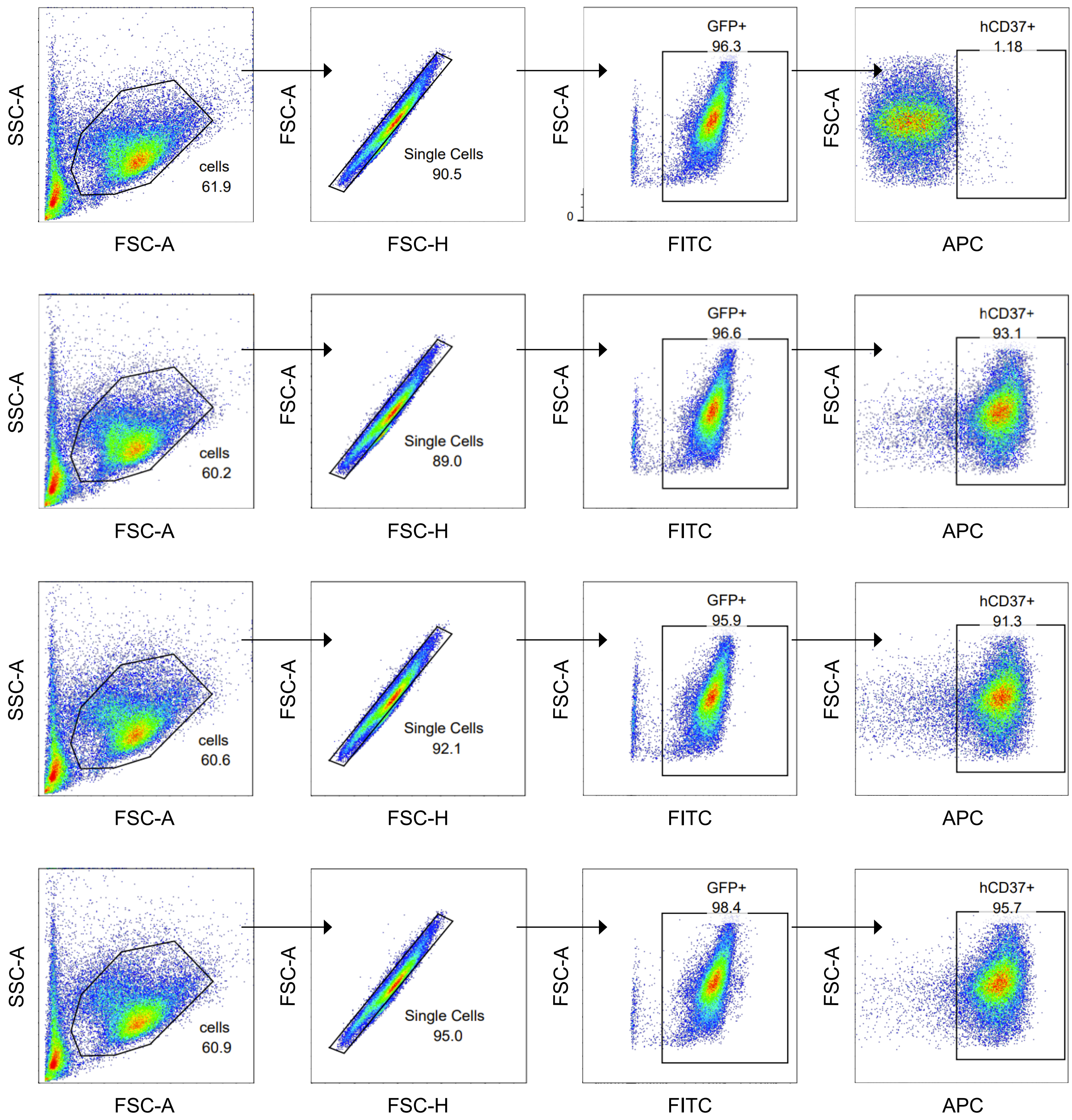

Figure S13. The resurgence of AML cells in the PDX animal is not due to antigen escape. Related to Figure 5. Human AM PDX cells were recovered from mouse spleen and analysed for CD37 expression. First raw: Gating strategy and Isotype control staining of isolated human cells from mouse spleen. Second raw: Gating strategy and CD37 staining of AML PDX cells from mouse 1. Third raw: Gating strategy and CD37 staining of AML PDX cells from mouse 2. Fourth raw: Gating strategy and CD37 staining of AML PDX cells from mouse 3.

Supplementary Table 1. AML patient cohort. Related to Figure 1.

Patient nr	Age	WBC at diagnosis	Diagnosis	De novo/sAML	FAB	ELN 2017 risk	Treatment	CR/nonCR	TX-status	5-year survival (months)	CD37 myeloid cells
**1**	35-60		AML	De novo	M5	I	STD	CR	Allo	>107	Low
**4**	35-60	57.8	AML	De novo	M5	I	STD	CR		6	Low
**5**	>60		MDS		M1	A	no treatment		Allo		Low
**6**	<35		AML	De novo	M4	F	STD	CR	Allo	>84	Low
**7**	35-60		AML	De novo	M4	I	unknown status	CR	Allo		Low
**8**	>60	102	CMML		M4	A	no treatment		Auto		High
**9**	>60		AML	De novo	M0	A	no treatment		Auto		Low
**11**	35-60	37.4	AML	De novo	M5	I	STD	CR	Allo	>144	Low
**12**	35-60		AML	De novo	M2	I	STD	nonCR	Allo	>144	Low
**13**	35-60		AML	De novo	M5	F	STD	CR	Allo	27	Low
**14**	35-60		AML	De novo	M5	I	STD	nonCR		>118	Low
**15**	35-60		AML	De novo	M0	I	STD	CR	Allo	73	High
**16**	>60	101	AML	De novo	M5	A	STD	CR	Allo	>103	Low
**17**	>60		AML	De novo	M5	I	STD	CR	Auto	>92	Low
**18**	35-60		AML	De novo	M1	F	STD	CR		>98	Low
**19**	35-60		AML	De novo	M1	I	unknown status			26	Low
**20**	35-60		AML	De novo	M5	I	STD	CR		7	High
**21**	35-60		AML	De novo	M2	I	STD	CR	Allo	27	Low
**22**	>60		AML	De novo	nt	A	no treatment				High
**23**	>60	29.8	AML	De novo	M1	I	STD	nonCR	Allo	22	Low
**24**	>60	214	AML	De novo	M5	I	STD			1	Low
**25**	35-60	241	AML	De novo	M2	I	STD	CR		>82	Low
**27**	35-60		AML	De novo	M4	F	STD	CR	Auto	>66	Low
**29**	>60		AML	De novo	M1	A	no treatment				High
**30**	>60	22.3	AML	De novo	M2	A	unknown status		Allo		High
**31**	>60	191	AML	De novo	nt	I	no treatment				High
**32**	>60	67	AML	De novo	M1	A	STD	CR	Allo	8	High
**33**	35-60	65.4	AML	De novo	M4	F	STD	CR		14	Low
**35**	>60	12.7	CMML		M5	A	no treatment		Allo	6	High
**36**	>60	82.3	AML	De novo	M5	I	STD	CR		1	Low
**38**	35-60	78	AML	tAML	M3	F	APL specific treatment	CR			High
**39**	>60	12.6	AML	De novo	M4	I	STD	CR		6	High
**40**	>60		AML	De novo	M1	A	no treatment		Allo		Low
**41**	>60	105	MDS		M4	I	no		NA		Low

							treatment				
**42**	35-60	46	AML	De novo	M1	F	unknown status				Low
**43**	>60		CMML		M5	I	no treatment				Low
**43**	>60	101	AML	Residiv	M2	I	no treatment				High
**46**	<35	111	AML	De novo	M5	I	STD	CR		>102	Low
**47**	>60	44.7	AML	De novo	M5	A	STD	CR		16	High
**48**	35-60		CMML		M5	A	no treatment				Low
**49**	35-60	96.9	AML	De novo	M2	I	STD	nonCR		29	Low
**50**	>60	65.3	AML	De novo	M1	I	STD	CR		5	High
**51**	>60	92	MF		M1	I	no treatment				High
**52**	>60	23	AML	De novo	M0	I	no treatment				Low
**53**	35-60		AML	De novo	M2	I	STD	CR		24	Low
**54**	>60		AML	De novo	M0	A	no treatment				Low
**55**	35-60	28.2	AML	De novo	M1	F	STD	CR		>80	Low
**56**	>60	nt	MDS		M2	I	unknown status			1	Low
**57**	35-60	34.5	AML	De novo	M4	I	STD	CR		>80	High
**58**	>60	47.5	AML	De novo	M4	F	unknown status				Low
**59**	>60	22.6	AML	De novo	M4	I	STD	CR		14	Low
**60**	>60	37.2	AML	De novo	M5	F	STD	CR		8	Low
**61**	>60	23.1	AML	De novo	M2	I	STD	nonCR		15	High
**63**	35-60	17.3	AML	De novo	M2	A	STD				Low
**64**	>60	71.4	AML	De novo	M2	F	no treatment				Low
**66**	>60	145	AML	De novo	M2	I	no treatment	CR			High
**67**	35-60	72.4	AML	De novo	M2	I	STD	CR		>75	Low
**68**	>60	5.6	AML	De novo	M1	I	STD	CR			Low
**69**	>60	nt	MF		M2	nt	no treatment				High

Supplementary Table 2. Mass Cytometry antibody panel for 59 AML patients and 5 healthy donors. Related to the STAR Methods, mass cytometry section.

Plus 16 bleed	Tag	Antibody	Clone	Used for Clustering	Comments
105	89 Y	CD45	HI30	x	Human Leukocytes
127	111 Cd	CD3	UCHT1	x	T cells
128	112 Cd	CD34	581	x	Hematopoietic stemcells and progenitors
129	113 Cd	CD123	6H6	x	AML blasts, basophils
130	114 Cd	CD7	2A3	x	HSC, NK cells and T cells, myeloblasts
132	116 Cd	HLA-DR	L243	x	AML blasts
155	139 La	CD8a	RPA-T8	x	T cell marker
157	141 Pr	CD19	HIB19	x	Activated B cells
158	142 Nd	Caspase 3 (Cleaved)	D3E9		Dying cells
159	143 Nd	CD45RA	Hi100	x	Naive T cells
160	144 Nd	CD38	HIT2	x	Basophils high, HSC low
161	145 Nd	CD4	RPA-T4	x	T cell marker, T helper cells, Monocytes and macrophage
162	146 Nd	CD64	10,1	x	Monocytes
163	147 Nd	pStat5 [Y694]	47		NRAS downstream
164	148 Nd	CD16	3G8	x	Granulocytes and NK cells
166	150 Nd	pRB (S807/811)	J112-906		phosphorylated retinoblasoma protein allowing cell cycle progression
168	152 Sm	CD66b	8OH3		Granulocytes
169	153 Eu	RUNX	EPR3099		AML biomarker
170	154 Sm	NRAS	EPR20278		AML biomarker
171	155 Gd	CD56	B159	x	NK cells and T lymphocyte
172	156 Gd	CD37	HH1		Potential AML biomarker, very positive in B cells.
174	158 Gd	CD33	WM53	x	Myeloid lineage,
175	159 Tb	CD90	5E10	x	HSC and MSC
176	160 Gd	CD14	M5E2	x	Monocytes
177	161 Dy	FLT3	S-18		Hematopoietic precursor marker, Common AML biomarker
178	162 Tm	Ki67	B56		Proliferation marker
179	163 Dy	CD105	43A3		MSC marker
180	164 Dy	Cyclin B1	GNS-1		G2/mitotic specific
181	165 Ho	CD45RO	UCHL1	x	Leukocyte common antigen, T cell activation
182	166 Er	CD44	x	x	Monocytes, T cells and ALML
183	167 Er	pErk 1/2 [T202/Y204]	D13.14.4		MAPK/ERK pathway, downstream of RAS
184	168 Er	cKit	YB5.B8		HSC and some AML
185	169 Tm	CD25	2A3	x	Activated T cells, Activated B cells, Myeloid precursor cells
186	170 ER	CSF1R (intracellular C-term)	9-4D2-1E4		Macrophages
188	172 Yb	CD73	EPR6115		MSC, fibroblast
189	173 Yb	CD300e	233810	x	Myeloid cells, activating receptor
190	174 Yb	CD11c	L243	x	Monocytes, macrophages and B cells, myeloblast
192	176 Yb	CD95/FAS	DX-2		Fatty acid synthesis, AML biomarker
225	209 Bi	CD11b	ICRF44	x	Neutrophils, NK cells and macrophage

Supplementary Table 3. AML and healthy cell description. Related to Figure 1.

Short	Abbreviation
HSC	Hematopoietic stem cell
MPP	Multipotential progenitors
CMP	Common myeloid progenitor cell
GMP	Granulocyte monocyte progenitors
MEP	Megakaryocyte-erythroid progenitor cell
early_PM	Early Promyelocyte
MM	Metamyelocytes
MY	Myelocyte
Mono	Monocytes
PMN	Polymorphonuclear cells
Normal	AML with Normal karyotype
Complex	AML with Complex karyotype
inv(16)	AML with inv(16)
t(15;17)	AML with t(15;17)
t(8;21)	AML with t(8;21)
t(11q23)/MLL	AML with t(11q23)/MLL
MDS	MDS
nan	AML with no karyotype information
Trisomy 8	AML with Trisomy 8
del(5q)	AML with del(5q)
del(7q)/7q-	AML with del(7q)/7q-
t(9;11)	AML with t(9;11)
Other	AML with Other abnormalities
7	AML with +7
Complex_ del(5q)	AML with Complex del(5q)
Complex_ untypical	AML with Complex untypical karyotype
ALL	ALL
inv(3)	AML with inv(3)
trisomy 11	AML with trisomy 11
trisomy 13	AML with trisomy 13
t(6;9)	AML with t(6;9)
t(8;16)	AML with t(8;16)
del(9q)	AML with del(9q)
t(1;3)	AML with t(1;3)
-5/7(q)	AML with -5/7(q)
-9q	AML with -9q
8	AML with +8
t(9;22)	AML with t(9;22)
abn(3q)	AML with abn(3q)

Supplementary Table 4. CyTof antibody panel used for the phenotypic characterization of the CAR T cell population. Related to Figure 5.

Tag	Antibody Target	Clone	Target significance	Concentration
89Y	hCD45	HI30	human leukocytes	200
111Cd	CD4	RPA-T4	CART T helper cells	2 µg/mL
113Cd	CD8	HIT8a	CART Cytotoxic T cells	0.25 µg/mL
116Cd	CD57	HNK-1	T cell differentiation, senescence	0.5 µg/mL
141Pr	CD196	G034E3	CART Homing/polarization	100
142Nd	OX40	ACT35	CART Activation	100
143Nd	Biotin	1D4C5	CD19 and CD37 CART cells	200
144Nd	CD38	HIT2	AML	100
145Nd	trCD34	4H11	CD33 CART cells	2 µg/mL
146Nd	GFP	FM264G	Luc+ PDX1 AML cells	6 µg/mL
147Sm	mCD45	30F11	mouse leukocytes	1600
148Nd	ICOS	C398.4A	CART Activation	100
149Sm	CD45RO	UCHL1	CART Effector/Memory	400
150Nd	LAG-3	11C3C65	CART Exhaustion	100
151Eu	CD123	6H6	AML	100
152Sm	Fas	DX2	CART Effector/Memory	200
153Eu	CD45RA	HI100	CART Effector/Memory	400
154Sm	TIM-3	F38-2E2	CART Exhaustion	100
155Gd	PD-1	EH12.2H7	CART Exhaustion	100
156Gd	CD37	HH1	AML (clone HH1)	10 µg/mL
158Gd	CD33	WM53	AML	100
159Tb	TIGIT	MBSA43	CART Exhaustion	100
160Gd	CD28	CD28.2	CART Activation	200
161Dy	CTLA-4	14D3	CART Exhaustion	200
162Dy	CD27	L128	CART Effector/Memory	400
164Dy	CD185	RF8B2	CART Homing/polarization	100
165Ho	CD19	HIB19	negative control	1000
167Er	CD197	G043H7	CART Effector/Memory	100
168Er	CD127	A019D5	CART Homing/polarization	80
169Tm	CD25	2A3	CART Activation	500
170Er	CD3	UCHT1	CART all T cells	400
171Yb	CD44	IM7	almost all cells	1600
172Yb	Ki-67	B56	CART proliferation	200
173Yb	4-1BB	4B4-1	CART Activation	200
174Yb	HLA-DR	L243	CART Activation	200
175Lu	CD184	12G5	CART Homing/polarization	100
176Yb	Histone 3	D1H2	all cells	100
209Bi	CD11b	ICRF44	AML	150
